# A molecular design approach towards elastic and multifunctional polymer electronics

**DOI:** 10.1038/s41467-021-25719-9

**Published:** 2021-09-29

**Authors:** Yu Zheng, Zhiao Yu, Song Zhang, Xian Kong, Wesley Michaels, Weichen Wang, Gan Chen, Deyu Liu, Jian-Cheng Lai, Nathaniel Prine, Weimin Zhang, Shayla Nikzad, Christopher B. Cooper, Donglai Zhong, Jaewan Mun, Zhitao Zhang, Jiheong Kang, Jeffrey B.-H. Tok, Iain McCulloch, Jian Qin, Xiaodan Gu, Zhenan Bao

**Affiliations:** 1 Department of Chemical Engineering, Stanford University, Stanford, CA USA; 2 Department of Chemistry, Stanford University, Stanford, CA USA; 3 School of Polymer Science and Engineering, The University of Southern Mississippi, Hattiesbury, MS USA; 4 Department of Materials Science and Engineering, Stanford University, Stanford, CA USA; 5 King Abdullah University of Science and Technology (KAUST), Kaust Solar Center (KSC), Thuwal, Saudi Arabia; 6 Department of Chemistry, Chemistry Research Laboratory, University of Oxford, Oxford, UK; 7 Department of Materials Science and Engineering, Korea Advanced Institute of Science and Technology (KAIST), Daejeon, Republic of Korea

**Keywords:** Electronic materials, Electronic devices, Polymers

## Abstract

Next-generation wearable electronics require enhanced mechanical robustness and device complexity. Besides previously reported softness and stretchability, desired merits for practical use include elasticity, solvent resistance, facile patternability and high charge carrier mobility. Here, we show a molecular design concept that simultaneously achieves all these targeted properties in both polymeric semiconductors and dielectrics, without compromising electrical performance. This is enabled by covalently-embedded in-situ rubber matrix (iRUM) formation through good mixing of iRUM precursors with polymer electronic materials, and finely-controlled composite film morphology built on azide crosslinking chemistry which leverages different reactivities with C–H and C=C bonds. The high covalent crosslinking density results in both superior elasticity and solvent resistance. When applied in stretchable transistors, the iRUM-semiconductor film retained its mobility after stretching to 100% strain, and exhibited record-high mobility retention of 1 cm^2^ V^−1^ s^−1^ after 1000 stretching-releasing cycles at 50% strain. The cycling life was stably extended to 5000 cycles, five times longer than all reported semiconductors. Furthermore, we fabricated elastic transistors via consecutively photo-patterning of the dielectric and semiconducting layers, demonstrating the potential of solution-processed multilayer device manufacturing. The iRUM represents a molecule-level design approach towards robust skin-inspired electronics.

## Abstract (web summary)

Next-generation skin-inspired electronics require enhanced mechanical robustness and device complexity including elasticity, solvent resistance, and facile patternability. Here, the authors show a molecular design concept that simultaneously achieves all these requirements by covalently linking an in-situ formed rubber matrix with polymer electronic materials.

## Introduction

Skin-like electronics have garnered considerable interest over the past decade for their potential applications in robotics, prosthetics, health monitoring, and medical implants^1^. Currently, stretchable electronics are made by either applying geometric designs on rigid inorganic-based devices using buckled substrates^2,3^ or developing stretchable organic electronic materials through molecular-level design^4,5^. Nevertheless, softness and stretchability reported before are still far from the requirements for realistic consumer electronics^6^. Besides, high electrical performance combined with elasticity, solvent resistance, and facile patternability are desired merits from both daily-use and practical manufacturing perspectives; however, each property usually requires a particular molecular design and has not been possible concurrently.

Polymer electronic materials have been identified as promising candidates due to their potential mechanical flexibility, structural tunability, solution processability, and cost-effectiveness^7,8^. Albeit theoretically promising, the key components, i.e., semiconductors and dielectrics, still face challenges. First, many previously reported polymer electronic materials are either brittle or viscoelastic, which causes unreliable hysteretic performance when subjected to harsh mechanical challenges, e.g. multiple, high-loading stretching-releasing cycles^9^, making these materials impossible for realistic long-term use. Second, for integrated circuits, sensor array, and display fabrication, the semiconductors and dielectrics need to be prepared through a simple yet universal solution-based deposition method, and should be compatible with multilayer device fabrication. However, solution-based processing is usually accompanied by poor solvent resistance. This is thus a ‘mixed blessing’ for polymer electronics, since it results in good solution processability but suffers from chemical dissolution in later processing steps, limiting the possibility of low-cost and scalable production^10^. Third, facile patternability is another critical merit for polymer electronic materials. Previous patterning methods include protection-etching that requires multiple steps involving sacrificial layer and orthogonal chemicals^11^, and inkjet printing that has difficulties in producing uniform devices^12^.

To overcome mechanical challenges, developing a stretchable and even elastic (reversible under cyclic loading-unloading) semiconductor without sacrificing electrical performance is a desired yet difficult objective^13^. Most of the conjugated polymers experience device failure under low strain (<10%) due to their semi-crystalline thin-film morphology^14^. Virtually all previously reported molecular design rules focused on enhancing the ultimate fracture strains of polymer semiconductors without loss of electronic functionalities, in which ‘reducing long-range crystalline order’ has been a general principle^15^. However, the semiconductor films in these cases suffered from permanent plastic deformation prior to crack formation. Upon removal of strain, the semiconductor film supported on an elastic dielectric tends to form wrinkles or buckles. Unfortunately, these processes invariably lead to interfacial delamination and degrade charge transport^16^. For practical applications, the semiconductor needs to function after multiple strain cycles, and thus elasticity/reversibility beyond simple stretchability while maintaining high charge transporting ability is needed. This requires high crosslinking density as well as chain flexibility in the active layer^17^, while balancing charge transport over multiple length scales.

To achieve chemical robustness as well as facile patternability, photo-patterning using crosslinking chemistry is a viable approach, which generates covalent crosslinking in polymer electronic materials^18,19^. However, introducing crosslinkers usually results in higher elastic modulus and lower fracture strain than the pristine semiconductors and dielectrics^18^. Limited examples of crosslinkers have been observed to increase the fracture strain of semiconductors; unfortunately, they all dramatically degraded the charge carrier mobility at a mere 5 wt.% addition due to the disruption of semiconductor aggregation. These crosslinked semiconductors still exhibited plastic deformation, i.e., irreversible stretchability, due to limited crosslinking density^20,21^. Another reported approach was blending a polymer semiconductor with a non-crosslinked elastomeric insulating polymer^22–25^. However, the high percentage of the insulating polymer in the blend rendered the film more susceptible to dissolution in organic solvents than neat semiconductor. In addition, most of the blended insulating polymers are still viscoelastic rather than elastic/reversible. Due to the absence of covalent crosslinking sites, the long-term mechanical stability remains uncertain. For dielectrics, some fluorinated elastomers have been reported to be organic solvent resistant, but they either showed double-layer capacitive effect^26^, or relied heavily on surface modification to achieve suitable wettability for semiconductor solution deposition^12^.

Overall, no general molecular design approach so far can simultaneously achieve elasticity, solvent resistance, and facile patternability without degrading electrical performance.

## Results

### Design principles of iRUM approach

Here, we report rationally designed single precursors for covalently embedded in-situ rubber matrix (iRUM) formation, which can undergo both self-crosslinking and crosslinking with the corresponding electronic material in finely controlled ratio, to achieve all targeted properties for both polymeric semiconductors (PSC) and dielectrics.

**Fig. 1:**
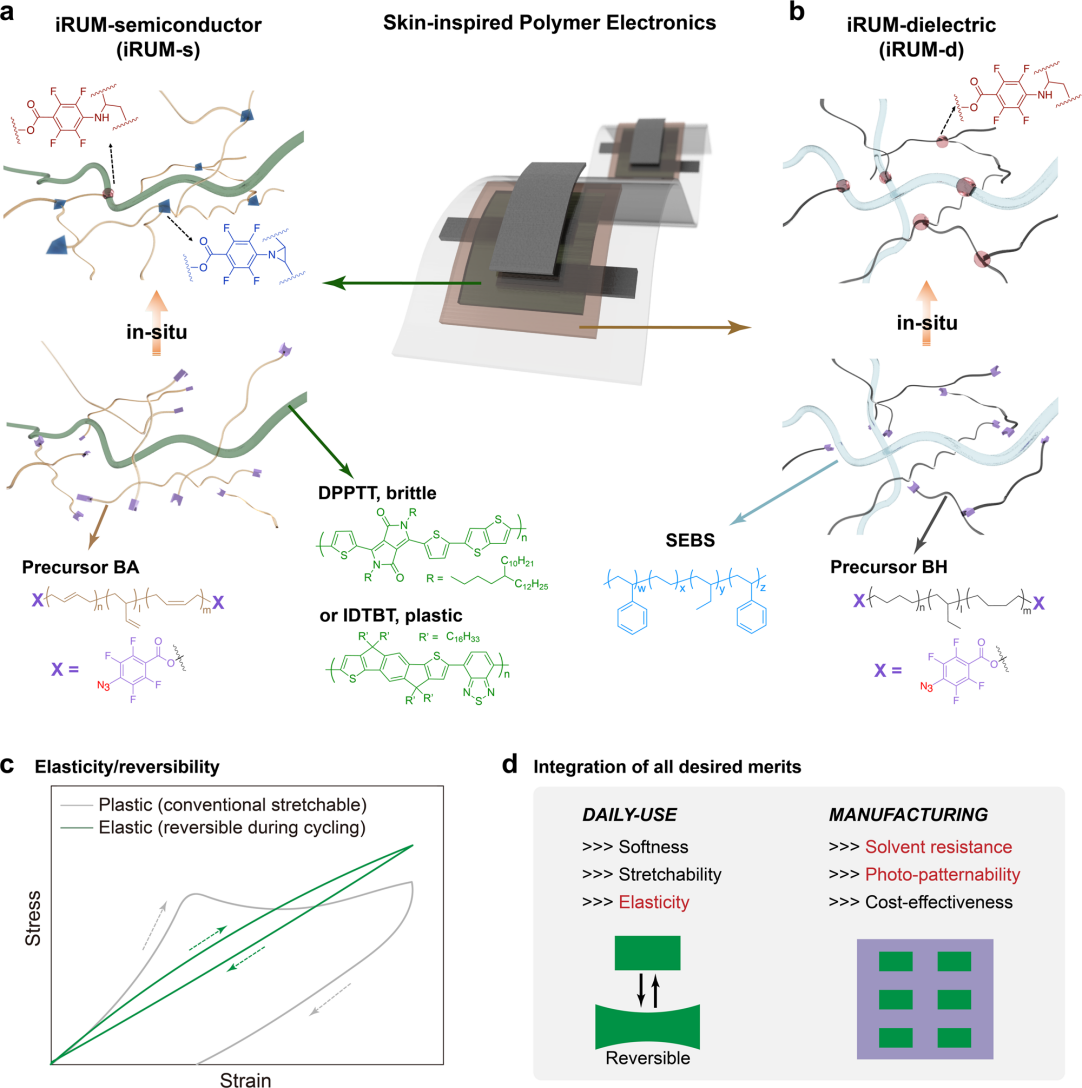
Schematic illustration of iRUM approach for both semiconductors and dielectrics. **a** For semiconductors, DPPTT network is covalently embedded into the in-situ formed elastic rubber matrix generated by iRUM precursor BA. In iRUM-s, the number of covalent crosslinking sites created through azide/C=C cycloaddition is much higher than that created through azide/C–H insertion. **b** For dielectrics, SEBS is uniformly crosslinked by iRUM precursor BH through azide/C–H insertion. The good miscibility between iRUM precursors and DPPTT/SEBS significantly improves the covalent crosslinking density in both semiconductor and dielectric layers. **c** The typical shape of cyclic stress-strain curves of plastic (conventional stretchable) PSC and elastic PSC demonstrated in this work. Conventional stretchable PSC undergoes plastic deformation, with residue strain left after one single loading-unloading cycle. When such a semiconductor film is supported on an elastic dielectric, cyclic strain results in wrinkling and buckling which may lead to interfacial delamination, and significantly degrade charge transport. **d** iRUM approach achieves the integration of all desired merits for skin-inspired polymer electronics from both daily use and manufacturing perspectives, without compromising electrical performance.

Briefly, for semiconductors, an iRUM precursor consisting of perfluorophenyl azide (PFPA) end-capped polybutadiene, BA (Fig. 1a and Supplementary Information Synthetic Methods), was designed to enable the following key features:

(i) The flexible backbone structure and compatible surface energy (30.4 mJ/m^2^) of BA enabled its good mixing (without forming micrometer-sized large separated domains) with the PSC (30–33 mJ/m^2^) in a high BA-to-PSC ratio, allowing for high crosslinking density.
(ii) BA was found to undergo self-crosslinking to generate a stretchable and elastic matrix through azide/C=C cycloaddition^27^ (Supplementary Fig. 1), resulting in a ‘semiconductor-in-rubber’ composite film.
(iii) The azide groups of BA also reacted with and crosslinked the alkyl side chains on polymer semiconductors through azide/C–H insertion^28^ (Supplementary Fig. 1). The created covalent bonding between BA and PSC is necessary for solvent resistance and photo-patternability.
(iv) A much higher proportion of BA underwent self-crosslinking described in (ii) rather than crosslinking with PSC in (iii), since the reactivity of cyclization is seven times faster than that of azide/C–H insertion^29,30^. Therefore, the PSC aggregation was hardly disrupted, resulting in maintained charge transport pathways.

With these features, the PSC network was covalently linked to the as-formed elastomeric matrix (Fig. 1a and Supplementary Fig. 1). Moreover, the hydroxyl-terminated polybutadiene is a widely utilized ingredient in the rubber industry for nearly a century and is available at low cost and large scale^31^.

For the dielectrics, an iRUM precursor consisting of PFPA end-capped hydrogenated-polybutadiene, BH (Fig. 1b and Supplementary Information Synthetic Methods), was designed to crosslink a widely used stretchable dielectric material, ﻿polystyrene-block-poly(ethylene-co-butylene)-block-polystyrene (SEBS). The high crosslinking density of the dielectrics is critical to realize its elasticity and provides superior solvent resistance which is necessary for direct depositing and photo-patterning of semiconductors on it.

Two important advances were achieved by iRUM and proved later in the paper:

(i) Different from conventional stretchable polymer semiconductors, the iRUM approach results in photo-patternable elastic semiconductors with high cyclic reversibility (Fig. 1c) without compromising charge carrier mobility.
(ii) The iRUM approach achieves all desirable merits (high electronic performance, softness, mechanical stability and ease of manufacturing) for polymer electronic materials (Fig. 1d).

### iRUM precursors and matrix formation

**Fig. 2:**
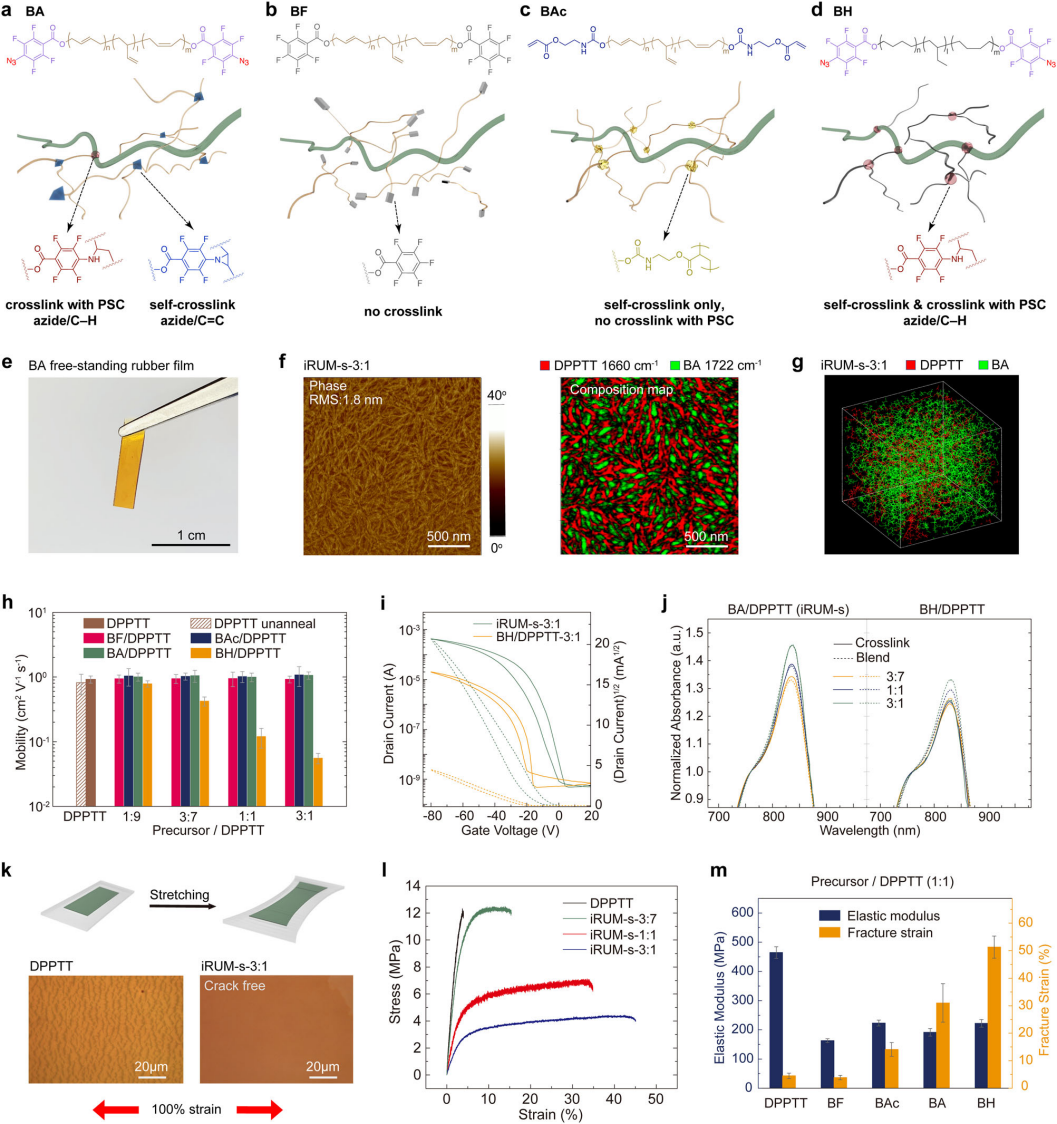
Systematic investigation of molecular design principles of iRUM semiconductors. The obtained semiconductor film is named ‘iRUM-s-x:y’, in which ‘s’ stands for semiconductor and ‘x:y’ is the BA-to-DPPTT weight ratio. Chemical structures of different polybutadiene-based precursors **a** BA, **b** BF, **c** BAc, **d** BH, and the generated semiconductor film after blending with DPPTT followed by thermal annealing at 150 ^o^C. **e** The free-standing elastic rubber film created by BA through azide/double bond cycloaddition. **f** AFM phase image and AFM-IR overlay image of iRUM-s-3:1 film. The surface roughness is obtained from the AFM height image. In the composition map, red color represents the DPPTT phase probed by IR laser at 1660 cm^−1^ and the green color represents the BA phase probed at 1722 cm^−1^ respectively. **g** Screen shot of MD simulated iRUM-s-3:1 film (simulation box length: ~5.8 nm), showing distributions of DPPTT and BA in a blend. **h** The extracted mobility of iRUM-s-x:y (BA/DPPTT-x:y) films, BF/DPPTT-x:y blend films, BAc/DPPTT-x:y and BH/DPPTT-x:y crosslinked films where x:y is the precursor-to-DPPTT weight ratio, characterized in bottom-gate top-contact transistors using SiO_2_ (300 nm)/Si as dielectric and gate electrode. **i** The representative transfer curves for iRUM-s-3:1 and BH/DPPTT-3:1 films. **j** UV–vis spectrum for iRUM-s and BH/DPPTT films prior and after thermal crosslinking. **k** Schematic illustration of the stretching of the semiconductor film on a supported PDMS substrate. Optical microscope images of a neat DPPTT film and an iRUM-s-3:1 film under 100% strain. **l** The representative stress-strain (engineering) curves for DPPTT and iRUM-s-x:y films obtained from pseudo-free-standing tensile tests^34,35^. **m** The extracted elastic modulus and fracture strain of iRUM-s (BA/DPPTT) films, BF/DPPTT blend films, BAc/DPPTT and BH/DPPTT crosslinked films obtained from pseudo-free-standing tensile tests (the precursor-to-DPPTT weight ratio is 1:1).

We first investigated the iRUM approach in semiconductors. In order to rationalize the molecular design principles of BA (Fig. 2a), we synthesized different polybutadiene-based precursors (Supplementary Information Synthetic Methods):

(i) polybutadiene-fluorine (BF): structurally similar to BA but cannot crosslink (Fig. 2b),
(ii) polybutadiene-acrylate (BAc): can only undergo self-crosslink to form an elastic rubber network, but no covalent crosslink with PSC (Fig. 2c), and
(iii) polybutadiene-hydrogenated-azide (BH): serves as a reference to compare the effect of reactivity difference between azide/C–H insertion and azide/C=C cycloaddition on the semiconductor electrical performance (Fig. 2d).

As described above, we observed that the iRUM precursor BA can react with itself (self-crosslink) to form an elastic rubber matrix. It showed no residue strain after repeated stretching-releasing cycles (Fig. 2e, Supplementary Fig. 2). Next, we chose a widely used high mobility donor-acceptor (D-A) conjugated polymers, poly-thieno[3,2-b]thiophene-diketopyrrolopyrrole (DPPTT) (M_n_: 62.7 kg/mol, PDI: 2.9) as a model semiconductor to investigate the feasibility of our approach. The obtained semiconductor film is named ‘iRUM-s-x:y’, in which ‘s’ stands for semiconductor and ‘x:y’ is the BA-to-DPPTT weight ratio. As observed from atomic force microscopy (AFM), iRUM-s-3:7, -1:1, and -3:1 all exhibited uniform morphology (Fig. 2f, Supplementary Fig. 3) and low surface roughness (1.8–2.4 nm), even though the weight of BA is up to three times that of DPPTT. In addition, the composition map of the film surface obtained through AFM paired with infrared spectroscopy (AFM-IR) confirmed the well-dispersed DPPTT network within the continuous BA-formed rubber matrix (Fig. 2f, Supplementary Fig. 4). Molecular dynamics (MD) simulations suggest a stronger association between BA and DPPTT side chains than that between BA and DPPTT backbone (Fig. 2g, Supplementary Figs. 5 and 6). We hypothesize that the bi-continuous network formed by BA and polymer semiconductor serves as the key premise in ensuring abundant crosslinking group in composite film and thus high covalent crosslinking density (Supplementary Table 1). Similarly, the blended or crosslinked DPPTT films with BF, BAc or BH all exhibited uniform mixing (Supplementary Figs. 7–12). We believe these morphologies originate from the surface energy match between the two components (conjugated polymer and polybutadiene-derived precursor), and the high flexibility of the polybutadiene backbone. This flexibility can prevent large-domain (micro-scale) phase separation driven by crystallization of the molecules, as has been observed in other systems^20^ (Supplementary Note 1).

### Maintained semiconductor electrical performance

The electrical performance of iRUM-s-x:y films was characterized in bottom-gate top-contact transistors with highly doped Si as a gate electrode, MoO_3_/Au as source and drain electrodes and octadecyltrimethoxysilane (OTS)-modified SiO_2_ as dielectrics. As shown by transfer curves and extracted charge carrier mobilities (Figs. 2h and 2i, Supplementary Fig. 13), this iRUM approach did not adversely affect the electrical performance of semiconductors, with all mobilities being maintained at ~1 cm^2^ V^−1^ s^−1^ despite the addition of different BA proportions. This mobility value is similar to that of the neat DPPTT. We hypothesize that this is due to the well-controlled ratio of C=C cycloaddition (i.e., reaction between azide and polybutadiene backbone) versus C–H insertion (i.e., reaction between azide and side chains on polymer semiconductor during crosslinking), as confirmed later. Since the charge carrier mobilities in BF/DPPTT blend films (Fig. 2b) and BAc/DPPTT crosslinked films (Fig. 2c) were also unaffected, it indicates that blending with polybutadiene precursors and the in-situ elastic rubber matrix formed inside the polymer semiconductor network will not disrupt the charge transport pathways (Fig. 2h, Supplementary Figs. 14 and 15). On the contrary, the mobility was observed to suffer a drastic decay in BH/DPPTT crosslinked films (0.12 cm^2^ V^−1^ s^−1^ for BH/DPPTT-1:1 and 0.06 cm^2^ V^−1^ s^−1^ for BH/DPPTT-3:1), as the BH concentration increases (Fig. 2h and i, Supplementary Fig. 16). To better understand this drastic difference, we performed multiple morphological characterizations. As revealed by the depth profiles of X-ray photoelectron spectroscopy (XPS), the semiconductor component in both iRUM-s-1:1 and BH/DPPTT-1:1 crosslinked film showed a similarly uniform distribution across film thickness (Supplementary Figs. 17–21). Therefore, the difference in electrical performance cannot be simply explained by the different vertical distribution of DPPTTs.

Based on the above observations, we hypothesize that the difference in reactivity may be contributing. BA may mostly have reacted with its double bonds to create a rubber matrix^29,30^, instead of reacting with the side chains of semiconductor that will disrupt chain packing and aggregation, thus able to maintain its charge transport pathway. The higher reactivity of azide/C=C cycloaddition than that of azide/C–H insertion is supported by thermogravimetric analysis (TGA) and attenuated total-reflectance Fourier transformation infrared spectroscopy (ATR-FTIR) (Supplementary Figs. 22 and 23). Different from BA, the all-single-bond backbone on BH is chemically indistinguishable with the long side chains on semiconductors, resulting in their equal reactivity with azides and potentially more disruption of the semiconductor aggregation^20^. This is supported by ultraviolet-visible absorption spectra (UV–vis) results. Specifically, the semiconductor aggregation in BAc/DPPTT and BA/DPPTT (iRUM-s) slightly increased in crosslinked films compared to their simply blended films without crosslinking; while on the contrary, a clear decrease in aggregation was observed in BH/DPPTT films after crosslinking (Fig. 2j, Supplementary Figs. 24 and 25). In addition, as indicated by grazing-incidence X-ray diffraction (GIXD), the edge-on coherence length slightly increased in iRUM-s-x:y films compared with the neat DPPTT film, while the mean coherence length decreased in BH/DPPTT crosslinked films (Supplementary Figs. 26–29, Tables 2–5, Note 2).

In summary, previous semiconductor/small molecule crosslinked systems fell short in simultaneously realizing high covalent crosslinking density (essential for elasticity) and maintaining decent charge carrier mobility^20,21,32^. While in our system, this challenging obstacle was overcome by leveraging the reactivity difference between azide/C–H insertion and azide/C=C cycloaddition, and selecting the appropriate rubber precursor structure to induce desired morphology for facilitated charge transport.

### Softness and stretchability

Next, the stretchability of iRUM-s-x:y was studied by applying various levels of strain on ﻿a polydimethylsiloxane (PDMS)-supported thin film^33^. For iRUM-s-1:1 and -3:1, they can be stretched up to 100% strain without showing any visible cracks. In contrast, neat DPPTT film showed dense and micrometer-scale cracks at 100% strain (Fig. 2k, Supplementary Fig. 30). Furthermore, pseudo-free-standing tensile tests were performed by floating the semiconductor film on water, which provided characterization of intrinsic thin-film mechanics^34,35^. From our obtained stress-strain curves, the iRUM-s-3:1 film exhibited nearly one-order-of-magnitude increase in fracture strain and four-time decrease in elastic modulus compared to neat film (Fig. 2l, Supplementary Fig. 31). This significant improvement in stretchability and softness originates from the increased proportion of the rubber matrix created by BA, which has a much lower elastic modulus (6.9 MPa) and higher fracture strain (145.9%) than that of pristine DPPTT (464.9 MPa and 4.5%, respectively) (Supplementary Figs. 2 and 31). When such an iRUM-s film undergoes mechanical deformation, the in-situ-formed crosslinked matrix provides stretchability and elasticity. Without crosslinking, the blend film of BF/DPPTT exhibited a much lower elastic modulus than neat DPPTT film, which was attributed to a plasticizing effect caused by the non-crosslinked polybutadiene^36^. However, its fracture strain is still as low as 3.9% and cracks still form at 50% strain despite reduced crack sizes. The other two types of semiconductor films with crosslinked matrices, BAc/DPPTT-1:1 and BH/DPPTT-1:1, also showed improved softness and stretchability than the neat film (Fig. 2m, Supplementary Figs. 32 and 33).

### Elastic and stretchable transistors

**Fig. 3:**
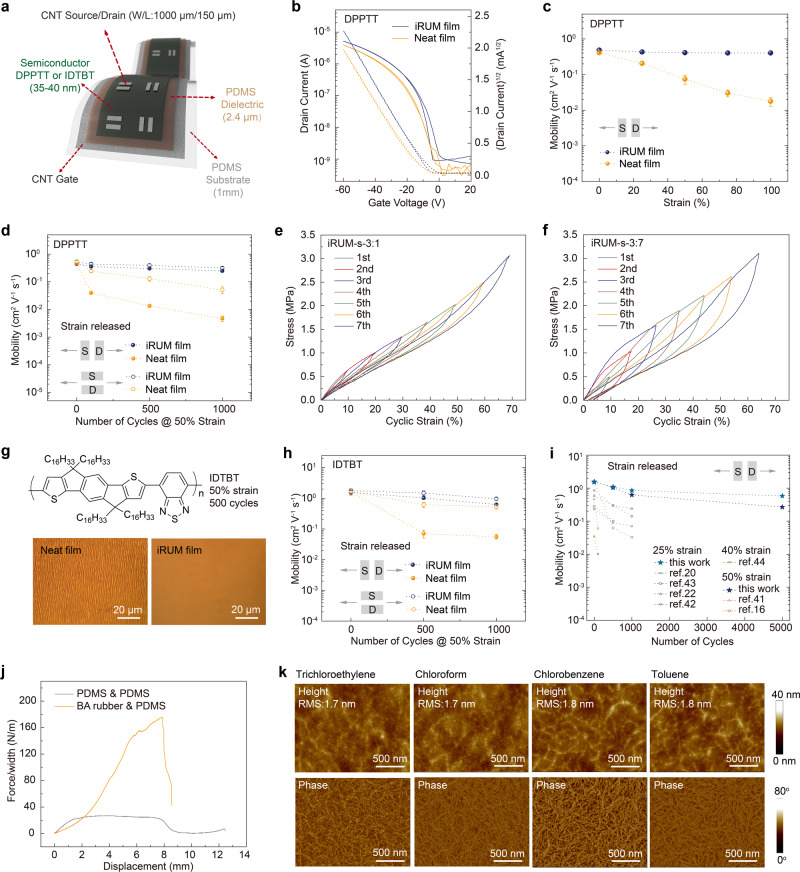
Characterization of the electrical performance of iRUM-s under mechanical deformation and solvent treatment. **a** Device structure of a fully stretchable transistor with a bottom-gate top-contact configuration, W/L = 1000 μm/150 μm. **b** Representative transfer curves of a stretchable transistor with neat DPPTT film or iRUM-s as the semiconductor. **c** Evolution in mobility at different strains during single stretching, with charge transport parallel to stretching direction. **d** Evolution in mobility after multiple stretching-releasing cycles at 50% strain under strain released state, with charge transport parallel and perpendicular to stretching direction. Stress-strain (engineering) curves with a cyclic strain range of 10–70% for iRUM-s-3:1 (**e**) and iRUM-s-3:7 (**f**) films, where an iRUM-s film (35 nm thick) was supported on a thin PDMS substrate (2.4 μm thick). **g** Optical microscope images of IDTBT and iRUM-IDTBT after 500 stretching-releasing cycles at 50% strain under strain released state. **h** Changes in mobility of a neat IDTBT film and an iRUM-IDTBT film after multiple stretching-releasing cycles at 50% strain under strain released state. **i** Comparison of the cyclic durability in this study with previously reported results in the literature^16,20,22,41–44^ (spin-coated semiconductor films using insulating polymer dielectrics under strain released state without applying other engineering techniques, which reflects the intrinsic properties of semiconductors). **j** Curves of the peeling force per width of PDMS sheet versus displacement for BA rubber and PDMS. The higher peeling force between BA rubber and PDMS indicates the formation of interfacial crosslinking. **k** AFM height and phase images of iRUM-s-3:1 after soaking in various organic solvents (trichloroethylene, chloroform, chlorobenzene, and toluene) for 30 s. The surface roughness is extracted from the AFM height image.

We proceeded to fabricate stretchable transistors to evaluate the electrical performance of iRUM-s under mechanical deformation, with carbon nanotube (CNT) as gate and source/drain electrodes and PDMS as dielectrics (Fig. 3a, Supplementary Fig. 34). As shown in a representative transfer curve, iRUM-s film showed ideal and comparative transistor performance as a neat film, with an average mobility of 0.5 cm^2^ V^−1^ s^−1^ (Fig. 3b). The decreased mobility compared to the rigid transistors originates from the higher contact resistance of CNT than Au electrode^22,37^. The mobility of iRUM-s was stably maintained despite the device being stretched up to 100% strain along the charge transport direction, while the neat film exhibited dramatic mobility degradation (Fig. 3c, Supplementary Figs. 35 and 36). As pointed out before, practical electronic devices need to reliably operate beyond a single stretching and maintain its functionalities under harsh cyclic loading. Therefore, we performed cyclic tests on the transistor at 50% strain (higher than typically applied strains needed for skin-inspired electronics, i.e., ~20–30%). The iRUM-s film showed remarkably stable charge carrier mobility (0.3 cm^2^ V^−1^ s^−1^) and on-current after 1000 stretching-releasing cycles under strain released state (Fig. 3d, Supplementary Figs. 37 and 38). This excellent cyclic durability was attributed to the robust elasticity of composite semiconductor film enabled by our iRUM approach.

To confirm our obtained elasticity improvement, we next performed mechanical tests for a bilayer specimen where an iRUM-s film (35 nm thick) was supported on a thin PDMS substrate (2.4 μm thick). This condition reflects the mechanical response of a semiconductor in a practical device configuration. A recent report demonstrated the feasibility of this film-laminated-on-thin-elastomer (FLOTE) approach to measure the viscoelastic behavior of a conjugated polymer thin film under large cyclic strains^38^. The viscoelasticity of conjugated polymer film was then extracted from the measurement on the bilayer structure and was found to significantly impact film stability after cycling. For our iRUM-s-3:1 film which was used in stretchable transistors, the stress-strain curves with increasing cyclic strains from 10 to 70% showed no residual strain upon strain removal (Fig. 3e). By contrast, the iRUM-s-3:7 film exhibited a ~4% residue strain due to lower rubber matrix content and thus plastic deformation (Fig. 3f), which is consistent with the observation of wrinkle formation after repeated stretching cycles observed via AFM (Supplementary Fig. 39). Furthermore, iRUM-s-3:1 film showed less stress relaxation and a much-reduced hysteresis (Supplementary Figs. 40 and 41), indicating the transition from viscoelasticity to entropy-driven elastic/reversible behavior^39^. When such a semiconductor film is deformed, the strain energy can be dissipated through conformational change of the in-situ formed rubber matrix, while the covalent crosslinking sites provide the film with ‘rebound force’ to return to its original state. The underlying mechanism was further cross-validated by the existence of residue strain and higher hysteresis as obtained from cyclic tests of BF/DPPTT-3:1 blend film and BAc/DPPTT-3:1 crosslinked film (Supplementary Figs. 42 and 43). To the best of our knowledge, this is the first direct demonstration of the elastic behavior of polymer semiconductor film under cyclic mechanical testing.

To examine the versatility of our iRUM approach, we applied it to another high-mobility D-A conjugated polymer, ﻿indacenodithiophene-co-benzothiadiazole (IDTBT)^40^ (M_n_: 104.5 kg/mol, PDI: 2.8). Different from semicrystalline DPPTT, IDTBT is known to exhibit a quasi-amorphous morphology and low energetic disorder^16^. We previously reported that IDTBT exhibited high stretchability (crack on-set strain >100% when supported on a PDMS substrate) but poor cyclic durability due to plastic deformation^16^, as confirmed by clear wrinkle formation and sharp mobility decrease (from >1 down to 0.07 cm^2^ V^−1^ s^−1^) after 500 stretching-releasing cycles at 50% strain (Fig. 3g and h). Currently, no approach has ever been reported to transform such a plastic semiconductor to an elastic one. After applying iRUM approach on IDTBT, the previously observed wrinkle formation for neat IDTBT after repeated stretching cycles was completely eliminated. Micrometer-sized large separated domains were not observed in the composite film, confirming the good mixing between BA and IDTBT (Supplementary Fig. 44). The average mobility was maintained at >1 cm^2^ V^−1^ s^−1^ even after 500 loading-unloading cycles at 50% strain under strain released state in fully stretchable transistors (Fig. 3g and h, Supplementary Fig. 45–49). This is already a record-high mobility retention compared to all other strategies reported in the literature thus far, and the applied cyclic strain level (50%) is two times higher than conventional strain level^16,20,22,41–44^ (Fig. 3i). The cycling life of our iRUM-s film can even be stably extended to 5000 cycles (four-hour continuous stretching-releasing), which is over five times longer than any previous attempt (Fig. 3i, Supplementary Fig. 50). As a comparison, we observed poor cyclic durability for non-crosslinked BF/IDTBT blend film, thus confirming again the elastic property of iRUM-IDTBT is a critical factor for the stable cycling performance rather than solely modulus match or interfacial crosslinking with dielectrics (Supplementary Figs. 51 and 52). Furthermore, we observed the stretchability of iRUM-IDTBT film was maintained while its softness was increased (reduced elastic modulus), as confirmed by pseudo free-standing tensile tests and crack on-set strain characterizations (Supplementary Figs. 53 and 54).

### Interfacial crosslinking

The accessible C=C double bonds of BA backbone located on the surfaces of iRUM-s film provide additional opportunities to engineer the semiconductor-dielectric interface. These double bonds are able to undergo various chemical reactions, e.g., hydrosilylation with silicon-hydride (Si﻿–H) and cycloaddition with azide. We reasoned that interfacial crosslinking can be created between BA rubber and PDMS through Si–H/vinyl reactions during the curing process, as evidenced by a much higher interfacial toughness obtained from 180^o^ peeling tests when compared to PDMS/PDMS interface^45^ (Fig. 3j). We observed that the PDMS portion fractured even before the breakage of BA/PDMS interface, while two pieces of PDMS can be readily separated (Supplementary Fig. 55, Supplementary Movie 1). Such a strong interfacial interaction ensures good adhesion and prevents delamination between semiconductor and dielectric, which is beneficial to stable operation of electronic devices under cyclic mechanical deformation over a long period of time.

### Solvent resistance and photo-patternability

**Fig. 4:**
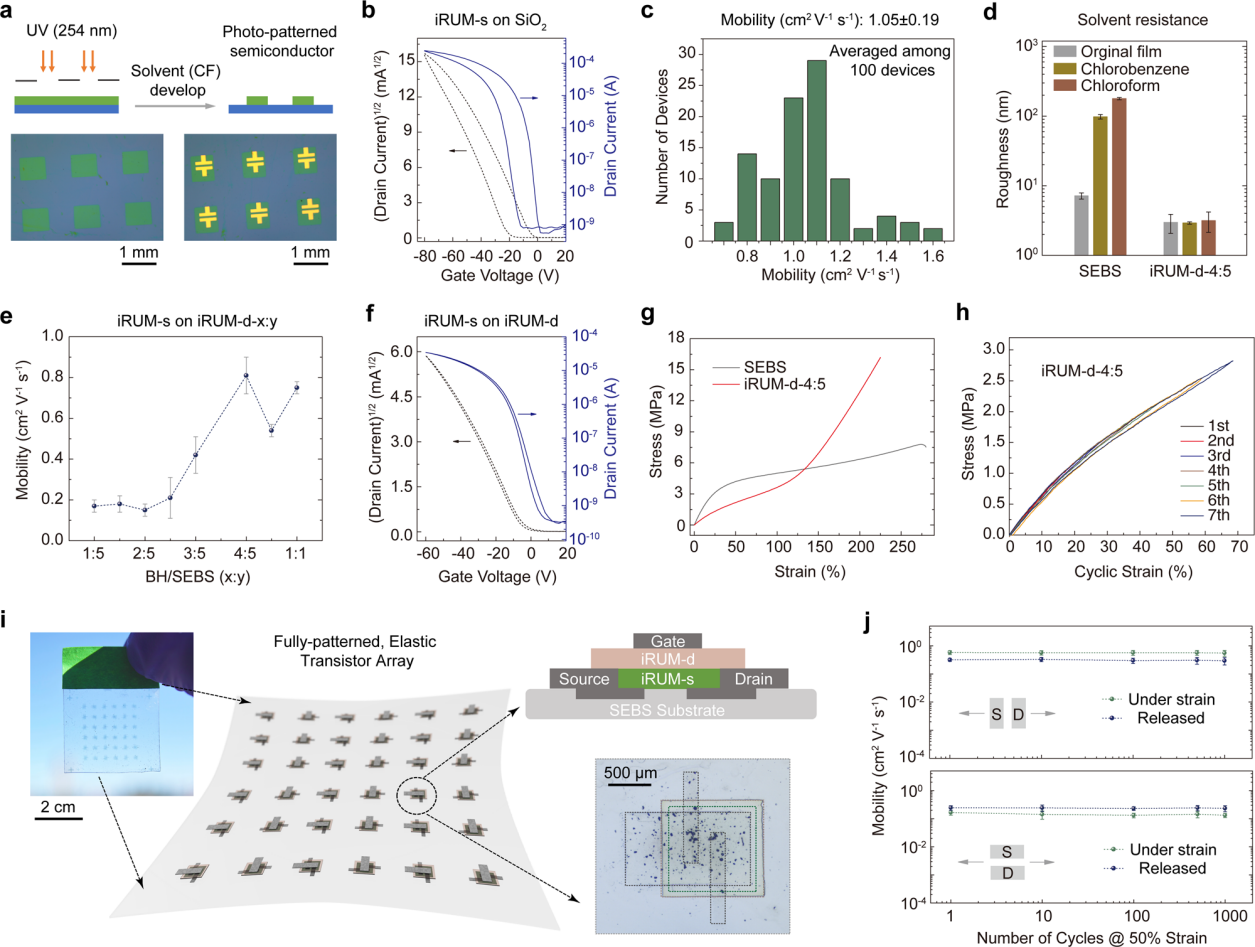
The photo-patternability of iRUM-s and iRUM-d, and the integrated patterned elastic transistors on a single substrate. **a** Schematic illustration of the photo-patterning process for iRUM-s, which involves selective UV exposure and chloroform development. Optical microscope images of photo-patterned iRUM-s film (thickness: 35 nm) before and after depositing MoO_3_/Au (2 nm/55 nm) electrodes. **b** A representative transfer curve of iRUM-s film that is photo-patterned on OTS-modified SiO_2_ (300 nm) as characterized in a bottom-gate top-contact transistor. **c** Mobility distribution of photo-patterned iRUM-s from 10×10 photo-patterned transistors on a single substrate, with bottom-gate top-contact configuration, highly doped Si as gate electrode, MoO_3_/Au as source/drain electrodes (*W* = 500 µm, *L* = 50 µm) and octadecyltrimethoxysilane (OTS)-modified SiO_2_ (300 nm) as dielectrics. **d** Surface roughness characterization of SEBS and iRUM-d-4:5 films by profilometer before- and after-solvent treatment. **e** The mobility of photo-patterned iRUM-s film on iRUM-d-x:y (1–1.5 μm thick), where x:y is the BH-to-SEBS weight ratio. **f** A representative transfer curve of a bottom-gate top-contact transistor with iRUM-s directly photo-patterned on iRUM-d-4:5 (1 μm thick). **g** The representative stress-strain curves for SEBS and iRUM-d-4:5 films obtained from pseudo-free-standing tensile tests. **h** Cyclic stress-strain curves (10–70% strains) for iRUM-d-4:5. **i** Schematic of fully patterned, stretchable, and elastic transistors on a single substrate. Two-dimensional diagram showing the side view of the transistor structure. **j** Changes in mobility for the patterned elastic transistors after multiple stretching-releasing cycles at 50% strain, with charge transport parallel and perpendicular to stretching direction.

The chemical crosslinking nature of the iRUM approach provides solvent resistance for polymer semiconductors, which is necessary for multilayer device fabrication. After treating a crosslinked iRUM-semiconductor film with various organic solvents, such as trichloroethylene, chloroform, chlorobenzene, and toluene which are commonly used to process polymer semiconductors, the surface remained smooth with low roughness (~1.7–1.8 nm) indicating little material being dissolved by solvent. In addition, the morphology was well-maintained as observed from both height and phase images by AFM (Fig. 3k). This observation was attributed to the covalent bonding between the in-situ formed BA rubber and polymer semiconductor network. On the other hand, BAc/DPPTT crosslinked film and BF/DPPTT blend film were found to be either partially or totally removed after organic solvent treatment. Therefore, the covalent bonding between BA and DPPTT created through C–H insertion is necessary for solvent resistance in addition to C=C cycloaddition of the BA matrix (Supplementary Fig. 56). Besides solvent resistance, we reasoned that since the crosslinking reaction of azide group can be activated by UV light, our iRUM approach can be used to photo-pattern polymer semiconductors. Through selective UV exposure (254 nm UV for 1 min), the photo-crosslinked regions of iRUM-s film will be immobilized, while the non-crosslinked regions can subsequently be ‘washed away’ using appropriate organic solvents (Fig. 4a). More importantly, no degradation was observed in the electrical performance of the iRUM-s film during photo-crosslinking and chloroform solvent development process. As a result, the photo-patterned semiconductor film exhibited equivalently high mobility compared with neat film, achieving an averaged mobility of 1.05 cm^2^ V^−1^ s^−1^ for 10×10 photo-patterned transistors on SiO_2_/Si substrate (Fig. 4b and c).

### Summary of iRUM-semiconductor

Our results underscore the following molecular design guidelines towards multifunctional PSC: (i) Uniform mixing of rubber matrix precursors with conjugated polymers is the premise of high covalent crosslinking density. (ii) Stretchability and even elasticity can be achieved through the as-formed crosslinked rubber matrix. (iii) Covalent crosslinking between precursor and PSC is necessary for solvent resistance and facile photo-patternability. (iv) The composite film morphology needs to be finely controlled through balancing the process of forming a rubber matrix and crosslinking PSC, in order to maintain charge transport. In addition to the polybutadiene backbone and crosslinkable azide end-group demonstrated in this study, we believe other rubber matrix precursors can potentially be adopted as long as they meet with the above requirements.

### iRUM-polymer dielectrics

Besides polymer semiconductors, we further applied our iRUM approach to a high-performance dielectric material, SEBS. Direct solution-based deposition of semiconductors on top of dielectrics is an industry-friendly process yet long-standing challenge in this field, so we test the iRUM strategy to address the above limitation. BH was chosen as the precursor to maximize the number of covalent crosslinking sites with SEBS (as elaborated above, BA tends to react more with itself while BH has relatively equal reactivity with both SEBS and itself) and to avoid current-voltage hysteresis that may arise from C=C as traps in the polybutadiene backbone^46,47^. The obtained dielectric is named ‘iRUM-d-x:y’, where ‘d’ stands for dielectric and ‘x:y’ is the BH-to-SEBS weight ratio. BH exhibited excellent mixing with SEBS, without large-domain phase separation occurring until BH/SEBS=1:1 (Supplementary Fig. 57). The iRUM-d film was measured to have a similar dielectric constant as SEBS (2.1–2.2) (Supplementary Fig. 58). To examine the solvent resistance of iRUM-d films, surface roughness characterization by profilometer before and after solvent treatment was conducted (Fig. 4d). iRUM-d-4:5 film remained smooth and uniform after depositing chlorobenzene or chloroform, with almost no change in roughness (~3 nm). In contrast, pristine SEBS film showed substantial swelling after solvent treatment, with roughness increased from 7 to 178 nm. iRUM-d films with lower crosslinking density showed less swelling but still increased surface roughness after solvent washing (Supplementary Fig. 59). Therefore, increasing the covalent crosslinking density in dielectrics resulted in its improved resistance against organic solvent attack.

Next, we fabricated bottom-gate top-contact transistors with iRUM-s directly deposited and photo-patterned on top of iRUM-d (highly doped Si as gate and MoO_3_/Au as source and drain electrodes). We observed that increasing the amount of BH in the crosslinked dielectric resulted in an increased charge carrier mobility of photo-patterned iRUM-s, with an averaged value of 0.8 cm^2^ V^−1^ s^−1^ when using iRUM-d-4:5, and the transistor exhibited ideal transfer characteristics with low hysteresis (Fig. 4e and f, Supplementary Fig. 60, Table 6). This condition was harsher than that encountered during the simple spin-coating or inkjet-printing process using semiconductor solution, as the materials need to survive multiple solvent washing steps. Notably, no further surface modifications on iRUM-d were needed to achieve the desired uniform iRUM-s film. In addition, despite the significantly increased crosslinking density of SEBS, the iRUM-d film showed negligible changes in fracture strain and, in fact, a 50% decrease in elastic modulus. As observed from stress-strain curves (Fig. 4g and h), iRUM-d (1.2 μm thick) exhibited strain-hardening behavior and reduced hysteresis from cyclic mechanical testing as compared to pristine SEBS (Supplementary Fig. 61), underscoring the iRUM-d superior elasticity.

### Fully patterned elastic transistors

Finally, iRUM-d can be successfully photo-patterned by UV (254 nm) exposure and solvent development (Supplementary Figs. 62–64). We have accordingly incorporated iRUM-s and iRUM-d into patterned elastic transistors on a single substrate, which serves as the building-block elements in functional circuits for signal processing and computation^48^, thus demonstrating the feasibility of integrating these newly developed materials and producing realistic skin-inspired electronics (Fig. 4i, Supplementary Fig. 65). Specifically, iRUM-d film was first photo-patterned on top of a water-soluble sacrificial layer ﻿poly(sodium-4-styrene sulfonate) (PSSNa)^49^. Next, iRUM-s was directly spin-coated and photo-patterned on the top of iRUM-d, greatly simplifying the conventional protection-etching process for patterning semiconductors (Supplementary Fig. 66)^11^. This takes not only the advantages of the photo-patternability of iRUM-s, but also the chemical robustness of iRUM-d, which makes it compatible with layer-by-layer solution deposition during device fabrication. After patterning the CNT source and drain electrodes, laminating substrate, releasing the device in water, and patterning CNT gate electrodes, we obtained an average charge carrier mobility of 0.4 cm^2^ V^−1^ s^−1^ and a high mobility retention after 1000 stretching-releasing cycles at 50% strain (Fig. 4j, Supplementary Figs. 67 and 68).

## Discussion

In summary, we have successfully demonstrated that covalently embedded iRUM formation is a simple and effective molecular design approach to simultaneously achieve mechanical robustness, facile patternability and high electrical performance for polymer electronic materials. The core idea is the good mixing combined with finely controlled composite film morphology built on azide chemistry, which takes advantage of its different reactivities with C–H and C=C bonds. Our iRUM approach results in an elastic, long-cycling semiconductor whose charge carrier mobility is comparable to that of amorphous Si and further increase is possible with other recently reported high mobility polymers. In addition, the developed materials are compatible with solution-processed multilayer device fabrication and can be incorporated into future complex integrated circuits. The iRUM strategy is also promising for mass production when considering the cost-effectiveness and scalability of precursors as well as the reduced cost of expensive active materials resulting from the high content of iRUM precursors (~50–75%). In addition to the transistors demonstrated in this work, the vertical uniformity of iRUM-semiconductor film opens up potential applications in vertical electronic devices such as organic light-emitting diodes and organic photovoltaics. The highly accessible and reactive double bonds in iRUM films further provide unique opportunities for pre- and/or post-modification and interfacial engineering through chemical functionalization. This work constitutes a milestone in molecular-level design for the transition from soft/stretchable to elastic and multifunctional skin-inspired electronics.

## Supplementary information

Supplementary Information

Description of Additional Supplementary Files

Supplementary Movie 1

## Data Availability

All data are available in the manuscript or Supplementary Information.
