# Supplementary material for: A molecular design approach towards elastic and multifunctional polymer electronics
Source: Nat Commun. 2021 Sep 29;12:5701. doi: 10.1038/s41467-021-25719-9 (PMC8481247; doi:10.1038/s41467-021-25719-9)
Supplement: Supplementary file 1 — Supplementary Information [file 41467_2021_25719_MOESM1_ESM.pdf]

Supplementary Information

A molecular design approach towards elastic and multifunctional polymer electronics

Yu Zheng[1,2]†, Zhiao Yu[1,2]†, Song Zhang[3], Xian Kong[1], Wesley Michaels[1], Weichen Wang[1,4], Gan Chen[1,4], Deyu Liu[1], Jian-Cheng Lai[1], Nathaniel Prine[3], Weimin Zhang[5,6], Shayla Nikzad[1], Christopher B. Cooper[1], Donglai Zhong[1], Jaewan Mun[1], Zhitao Zhang[1], Jiheong Kang[1,7], Jeffrey B.-H. Tok[1], Iain McCulloch[5,6], Jian Qin[1], Xiaodan Gu[3], Zhenan Bao[1]*

[1]Department of Chemical Engineering, Stanford University, Stanford, California, USA.
[2]Department of Chemistry, Stanford University, Stanford, California, USA.
[3]School of Polymer Science and Engineering, The University of Southern Mississippi, Hattiesbury, Mississippi, USA.
[4]Department of Materials Science and Engineering, Stanford University, Stanford, California, USA.
[5]King Abdullah University of Science and Technology (KAUST), Kaust Solar Center (KSC), Thuwal, Saudi Arabia.
[6]Department of Chemistry, Chemistry Research Laboratory, University of Oxford, Oxford, UK.
[7]Department of Materials Science and Engineering, Korea Advanced Institute of Science and Technology (KAIST), Daejeon, Republic of Korea.
† Yu Zheng and Zhiao Yu contributed equally to this work.
* Corresponding author, e-mail: zbao@stanford.edu

Content:

1. Supplementary Methods
2. Supplementary Figures 1-68, Tables 1-6, Notes 1-2, and Movie 1
3. Supplementary References

1. Supplementary Methods:

Materials

Polybutadiene (hydroxyl terminated, M_n ~1,200, M_w ~2,400), 2,3,4,5,6-pentafluorobenzoic acid, thionyl chloride and poly(sodium 4-styrenesulfonate) solution (average M_w ~70000, 30 wt. % in H_2O) were purchased from Sigma-Aldrich. 4-Azido-2,3,5,6-tetrafluorobenzoic acid and 2-isocyanatoethyl acrylate were purchased from TCI. Palladium (10% on carbon, Type 487, dry) was purchased from Alfa Aesar. Dow Corning Sylgard 184 silicone elastomer clear kit was used to prepare PDMS with different base/crosslinker mix ratio for different measurement. P2 and P3 SWNT were purchased from Carbon Solutions and used as received for fabrication of stretchable OFET. SEBS H1052 with volume fraction of poly(ethylene-co-butylene) = 80%, was provided by Asahi Kasei company and used as dielectrics. Poly-thieno[3,2-b]thiophene-diketopyrrolopyrrole (DPPTT)[1] and indacenodithiophene-co-benzothiadiazole (IDTBT)[2] were synthesized via previous reports. Other general reagents and solvents were purchased from Sigma-Aldrich or Fisher Scientific. All chemicals were used without further purification.

General Measurement and Characterization

Microwave polymerization was conducted using a Biotage Microwave reactor. Number average molecular weight (M_n), weight average molecular weight (M_w), and dispersity (Đ) were measured by high-temperature gel permeation chromatography (HT-GPC), using a Tosoh high-temperature EcoSEC equipped with a single TSKgel GPC column, calibrated with monodisperse polystyrene standards. The solvent was 1,2,4-trichlorobenzene with a flow rate of 1 mL/min under 180 °C. Thermal gravimetric analysis (TGA) experiments were performed on a TA instrument Mettler Toledo AG-TGA/SDTA851e under N_2, with heating ramp rate of 20°C/min from room temperature to 600°C. UV-Vis absorption spectrum was recorded on an Agilent Cary 6000i UV-Vis-NIR. Attenuated total-reflectance Fourier transformation infrared spectroscopy (ATR-FTIR) was performed on a Nicolet iS50 FT-IR Spectrometer with a diamond ATR crystal and DTGS detector. Nuclei magnetic resonance (NMR) spectra for newly synthesized molecules were recorded on a Varian Mercury console spectrometer (400 MHz,) at 293 K, Chemical shifts are given in parts per million (ppm) with respect to tetramethysilane as an internal standard and $CDCl_3$ as a solvent. Optical microscope images were obtained with a Leica DM4000 M LED microscope. The film surface morphology was obtained with a Nanoscope 3D controller atomic force micrograph (AFM, Digital Instruments) operated in the tapping mode at room temperature. Grazing-incidence X-ray diffraction (GIXD) was performed at the Stanford Synchrotron Radiation Lightsource on beamline 11-3 using a Rayonix 2-D area detector, with the incidence angle 0.12°. Samples were measured in a helium chamber and the data analysis was performed in WxDiff. Linecuts were derived for the meridian by integrating the intensity between $q_{xy} = -0.005$ and 0.005 Å^{-1} as a function of approximate q_z. Pseudo free-standing tensile test was performed through a motorized linear stage equipped with a digital encoder (Micronix Inc.) coupled with a high-resolution load cell (KYOWA Inc.) following the procedures

described below. All of the transistors were measured using a Keithley 4200 semiconductor parameter analyzer (Keithley Instruments Inc, Cleveland, OH, USA) under ambient atmosphere at room temperature.

AFM-IR: Atomic force microscopy paired with infrared spectroscopy (AFM-IR) is a surface characterization technique capable of probing both nanoscale topography and local chemical composition. Like traditional scanning probe microscopy, AFM-IR rasters a nanoscale probe across the surface either in contact or tapping mode. As the probe tracks along the material surface, a pulsed, tunable infrared laser excites the scan area and causes rapid thermal expansion within the material. This thermal expansion is detected by the AFM probe which is then translated into local chemical topography. Local composition can also be acquired by collecting a broadband infrared spectrum on the surface of the material where the AFM probe is positioned over a user-specified location and the infrared laser sweeps through a range of infrared frequencies. Samples are measured using a nanoIR3 AFM-IR from Anasys Instruments (Santa Barbara, CA) coupled to a MIRcat-QT™ quantum cascade, mid-infrared laser by Daylight Solutions (frequency range of 917-1700 cm^{-1} and 1900-2230 cm^{-1} and repetition rate of 1,470 kHz). AFM-IR data are collected in tapping mode using a gold-coated, AFM probe (spring constant (k): 40 N/m and resonant frequency (f_o): 300 kHz). The pulsed, mid-IR laser is tuned to frequencies unique to each component as determined by FTIR characterization. Acquired images are flattened using Analysis Studio software.

Molecular Dynamics Simulations

Molecular dynamics (MD) simulations were conducted to probe the mixing behavior of BA and DPPTT. MD simulations were conducted with the Gromacs 2018 program[3]. The force field is based on Optimized Potentials for Liquid Simulations all atom (OPLS-AA[4])[5]. Each BA chain has n, l, m = 9, 3, 10 with a unique random sequence of different monomers. The length of DPPTT is 5. Initially, polymer chains were randomly inserted into a large cubic box with a side length of 11 nm. Then the simulation box was compressed at a high pressure of 100 bar to reach a density close to the melt. After that, the system was simulated for 20 ns at a temperature of 430 K and a pressure of 1 bar. The snapshot was taken at the end of 20 ns simulation.

Thin-film Preparation and Characterization

(1) iRUM-s-x:y film preparation:

5 mg/mL DPPTT (or 10 mg/mL IDTBT) and 10 mg/mL BA stock solutions were first prepared with anhydrous chlorobenzene. Then, the BA solution and the DPPTT (or IDTBT) solution were mixed with a specific volume ratio to obtain BA/DPPTT (or IDTBT) blend with a designed weight ratio (x:y). (eg, 150 μL BA solution mixed with 100μL DPPTT solution to give a blend solution with BA/DPPTT-3:1). The blend solution was heated and stir under nitrogen at 85 °C for 15 min. The solutions were subsequently spin-coated on OTS (octadecyltrimethoxysilane)-modified substrates at 1500 revolutions per minute (r.p.m.) (3000 r.p.m. for IDTBT) for 1 minute and annealed at 150 °C for 40 min in nitrogen atmosphere. Film thickness is around 30-40 nm.

(2) Preparation of crosslinked BA rubber film:

Newly synthesized BA was drop-casted on OTS-modified substrate, and put in vacuum heater at RT for 5 days, 50 ^{o}C for 1 d, 60 ^{o}C for 1d, 80 ^{o}C for 2 d. The crosslinking process needs to be slow, otherwise bubbles will form and act as mechanical defects. The mechanical properties of as-prepared BA rubber film were tested by Instron. Film dimension: length 1.7mm, width 2.7mm, thickness 0.8mm.

(3) BAc/DPPTT-x:y crosslinked film preparation:

5 mg/mL DPPTT, 10 mg/mL BAc, and 25 mg/mL Azobisisobutyronitrile (AIBN) initiator stock solutions were first prepared with anhydrous chlorobenzene. Then, the BAc solution and the DPPTT solution were mixed with a specific volume ratio to obtain BAc/DPPTT blend with a designed weight ratio (x:y), followed by adding 5w% AIBN. (eg, 150 µL BAc solution mixed with 100 µL DPPTT solution and 3µL AIBN to give a blend solution with BAc/DPPTT-3:1). The blend solution was heated and stir under nitrogen at 85 °C for 15 min. The solutions were subsequently spin-coated on OTS-modified substrates at 1500 r.p.m. for 1 minute and annealed at 150 °C for 40 min in nitrogen atmosphere. Film thickness is around 30-40 nm.

(4) BH/DPPTT-x:y crosslinked film preparation:

5 mg/mL DPPTT and 10 mg/mL BH stock solutions were first prepared with anhydrous chlorobenzene. Then, the BH solution and the DPPTT solution were mixed with a specific volume ratio to obtain BH/DPPTT blend with a designed weight ratio. The blend solution was heated and stir under nitrogen at 85 °C for 15 min. The solutions were subsequently spin-coated on OTS-modified substrates at 1500 r.p.m. for 1 minute and annealed at 150 °C for 1.5 h in nitrogen atmosphere. Film thickness is around 30-40 nm.

(5) BF/DPPTT-x:y blend film preparation:

5 mg/mL DPPTT and 10 mg/mL BF stock solutions were first prepared with anhydrous chlorobenzene. Then, the BF solution and the DPPTT solution were mixed with a specific volume ratio to obtain BF/DPPTT blend with a designed weight ratio. The blend solution was heated and stir under nitrogen at 85 °C for 15 min. The solutions were subsequently spin-coated on OTS-modified substrates at 1500 r.p.m. for 1 minute. Film thickness is around 30-40 nm.

(6) Photo-pattern iRUM-s-x:y film:

The BA/DPPTT blend solution with a designed weight ratio was prepared following the procedures mentioned above. The solution was subsequently spin-coated on OTS-modified substrates at 1500 r.p.m. for 1 minute. The obtained film was photo-crosslinked in glovebox under UV light (254 nm, power 6W) for 1min (Dose: 270 mJ/cm^2) using shadow mask, and then developed in $CHCl_3$ in fume hood for 30 s. The patterned semiconductor film was post-annealed at 150 °C for 30 min in glovebox, in order to fully crosslink and get rid of solvent.

(7) iRUM-d-x:y film preparation:

90 mg/mL SEBS and 100 mg/mL BH stock solutions were first prepared with anhydrous toluene. Then, the filtered solutions were mixed with a specific volume ratio to obtain BH/SEBS blend with a designed weight ratio (x:y). The

blend solution was heated and stir under nitrogen at 85 °C for 15 min. The solution was subsequently spin-coated on highly doped Si substrates at 1000 r.p.m. for 1 minute and annealed at 200 °C for 1.5 h in nitrogen atmosphere. Film thickness is around 1-1.5 µm.

(8) Photo-pattern iRUM-d-x:y film:

The BH/SEBS blend solution with a designed weight ratio was prepared following the procedures mentioned above. The solution was subsequently spin-coated on substrates at 1000 r.p.m. for 1 minute. The obtained film was photo-crosslinked by exposure under deep ultraviolet light (wavelength 254 nm, using a Spectrum 100 Precision UV Spot Curing System from American Ultraviolet) for 30min with a dose of 126 J/cm^2 at ambient condition using shadow mask. After this, dodecane was used to dissolve the unexposed areas of SEBS/BH blend, with the photo-exposed areas preserved. The patterned dielectric film was post-annealed at 200 °C for 1 h in glovebox, in order to fully crosslink and get rid of solvent.

(9) Pseudo free-standing tensile tests (Film-on-water):

The Si substrate is cleaned with deionized water and isopropanol, followed by 1min O_2 plasma treatment. Then poly(sodium 4-styrenesulfonate), PSSNa solution (3 wt.% in deionized water) is spin-coated onto Si substrate at 4000 r.p.m. for 1 min, and annealing at 85 ºC for 15 min (thickness: ~30 nm). The semiconductor (or dielectrics) film is directly prepared on top of the water-soluble PSSNa layer. The polymer films were first patterned into dog-bone shape according to previous reports[6,7], followed by slowly dipping into deionized water to release and float the semiconductor film by dissolving the underneath PSSNa layer. Later, the semiconductor (or dielectrics) film was hold with two aluminum tensile grips coated with a thin PDMS layer, and then tensile tests were performed. For stress-strain measurements and cyclic tests, the strain rate is 0.05 s^{-1}. For stress relaxation, a strain rate of 0.03 s^{-1} is applied and the strain is kept within the elastic region (5%). The elastic modulus was obtained from the slope of the linear fit of the stress–strain curve using the first 0.5% strain (elastic region).

For cyclic tests and stress relaxation measurement, an additional thin layer of PDMS is directly prepared on top of the semiconductor film by spin-coating a dielectrics solution (2.8 g PDMS, base/crosslinker-6:1 in 10 mL hexane) at 5000 r.p.m. for 2 min, followed by annealing at 70 ºC for 1h in air and annealing in glove box at 150 ºC for 40 min. The resultant thickness for dielectrics is around 2.4 µm.

OFET Fabrication

(1) “Rigid transistor”:

The semiconductor thin film was prepared on OTS-treated SiO_2 dielectrics (300 nm, C= 10 nF/cm^2) or iRUM-d dielectrics. Top-contact MoO_3 (3 nm) and Au (40 nm) were subsequently deposited by thermal evaporation through a shadow mask with channel dimension of length L = 50 µm, width W = 1000 µm. For photo-patterning iRUM-s on SiO_2, the channel dimension is of length L = 50 µm, width W = 500 µm.

(2) "Stretchable transistor":

The semiconductor film (neat conjugated polymer or iRUM-s) was prepared on OTS-treated 300 nm SiO_2 substrate first. The solution for dielectrics was prepared by diluting 2.8 g PDMS (Sylgard 184 [6:1] base versus crosslinker in weight ratio) into 10 mL hexane, stir for at least 10min before spin-coating. Then the dielectric layer was prepared by directly spin-coating dielectric solution onto the semiconductor layer at 5000 r.p.m. for 2min, and anneal at 70°C for 1h in air. Then the substrate with the two layers was further annealed in glove box at 150 °C for 40 min. The resultant thickness for dielectrics is around 2.4 μm as confirmed by a Profilometer (Bruker Dektak XT). Carbon nanotube (CNT) solution for gate electrode was prepared by dispersing P2-SWNT (0.2 mg/mL) with P3HT (0.05 mg/mL) into chloroform through ultra-sonication for 30 min at 30% amplitude using a 750 W ultra-sonication probe, followed by centrifugation at 893.8 × g for 30 min. Then the dispersed CNT solution was spray-coated onto OTS-treated SiO_2 substrate using a commercial airbrush (Master Airbrush, Model SB844-SET) to obtain the gate layer. PDMS-based tough elastomer (PDMS-$MPU_{0.4}$-$IU_{0.6}$)[8] solution (30 mg/ml in chloroform) was spin-coated onto the gate layer at 3000 r.p.m. for 1 min to form an embedding layer. The stretchable substrate was prepared subsequently by spin coating PDMS (Sylgard 184 [12:1] base vs cross linker in weight ratio) at 500 r.p.m. for 30 s onto the embedding layer. Then the three layers were heated together at 70 °C overnight. The resultant thickness for substrate is around 1mm. The gate layer was used to lift-off the dielectric and semiconductor layer. Then we have the device structure from bottom to top as PDMS substrate, embedding layer, CNT gate electrode, PDMS dielectric and polymer semiconductor. For source/drain electrodes, CNT (P3-SWNT, Carbon Solutions Inc., 0.3 mg/mL in isopropanol) was sonicated overnight, followed by ultrasonication for 10 min at 30% amplitude and centrifugation at 503.1 × g for 20 min. The dispersed CNT was directly spray-coated onto the semiconductor through a shadow mask (L = 150 μm, W = 1000 μm) to obtain the patterned source/drain electrodes.

(3) Fully patterned transistor array fabrication:

A Si/SiO_2 wafer was firstly cleaned with O_2 plasma (150 W, 200 mTorr) for 1-2 min, and then PSSNa solution (3 wt. % in water) was spin-coated on top at 2500 r.p.m. for 1min. The wafer was then baked on a hot plate at 180 °C for 30 min and cooled down to 80 °C to fully get rid of water. Then iRUM-d was photo-patterned on top of PSSNa, following the procedures mentioned above, serving as the dielectrics with a thickness of about 1.2 μm. Subsequently, iRUM-s was photo-patterned on top of iRUM-d following the procedures mentioned above, serving as the semiconductor with a thickness of about 35 nm. For source/drain electrodes, CNT dispersed solutions (P3-SWNT, 0.3 mg/mL in isopropanol) were prepared and directly spray-coated onto the semiconductor through a shadow mask (L = 120 μm, W = 400 μm) to obtain the patterned source/drain electrodes. Then, a SEBS (H1221) stretchable substrate was laminated onto the fabricated devices on the Si/SiO_2 substrate. Then, the array was transferred onto the substrate by immersing the entire device in water to dissolve the sacrificial PSSNa layer. Finally, the gate electrodes were patterned on top of the SEBS dielectric by spray-coating the same CNT dispersed solution through a shadow mask. Alignment of the shadow masks was performed under optical microscopy.

Synthetic Methods

(1) 4-Azido-2,3,5,6-tetrafluorobenzoyl chloride

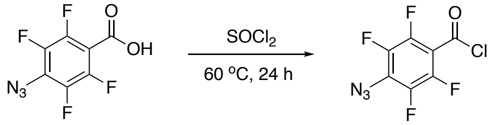

To a 100 mL round-bottom flask was added 6 mL anhydrous thionyl chloride (82 mmol). Under stirring at room temperature, 2 g of 4-azido-2,3,5,6-tetrafluorobenzoic acid (8.5 mmol) was added slowly in batches. The suspension was then heated to 60 °C to reflux for 24 h. After the reaction, the residue thionyl chloride was removed under vacuum. The product was cooled down to room temperature and filtered off through a 0.45-µm polytetrafluoroethylene filter to yield a light yellowish liquid. The obtained 4-azido-2,3,5,6-tetrafluorobenzoyl chloride used without further purification. Yield: ~90%.

^{19}F-NMR (376 MHz, $CDCl_3$, δ/ppm): -138.11 − -138.21 (2F, m), -150.18 − -150.28 (2F, m). ^{13}C-NMR (100 MHz, $CDCl_3$, δ/ppm): 158.39, 146.00−143.17, 141.91−139.23, 125.76, 111.86.

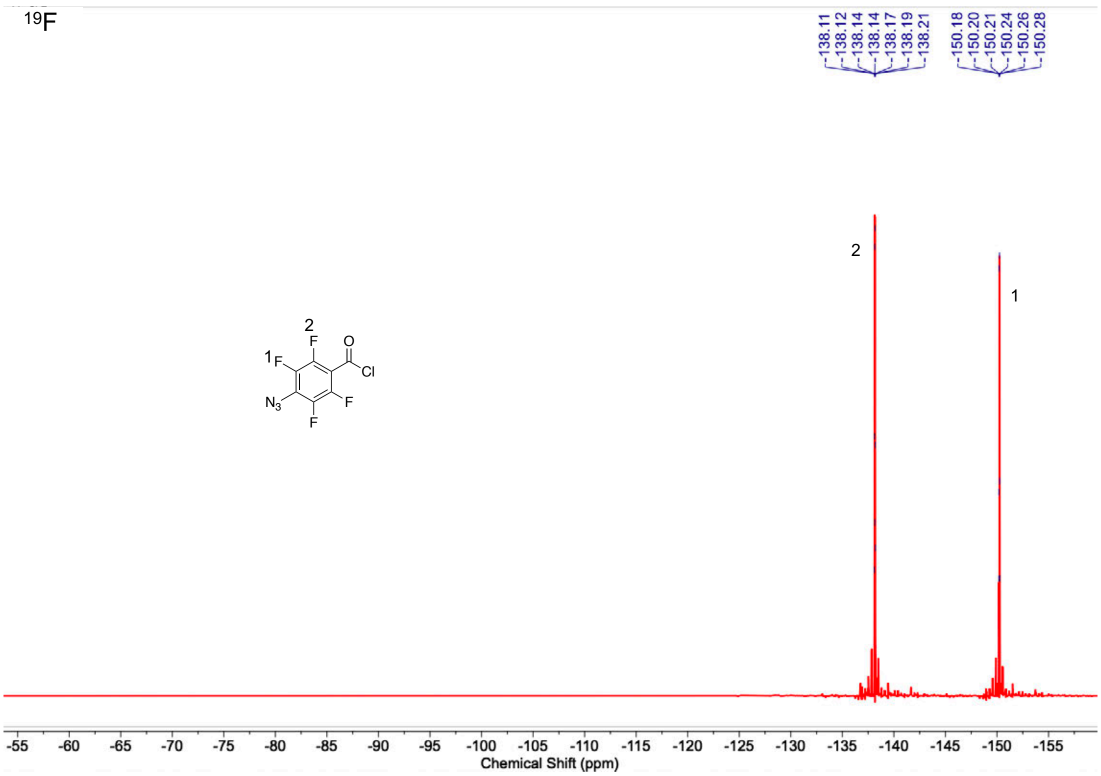

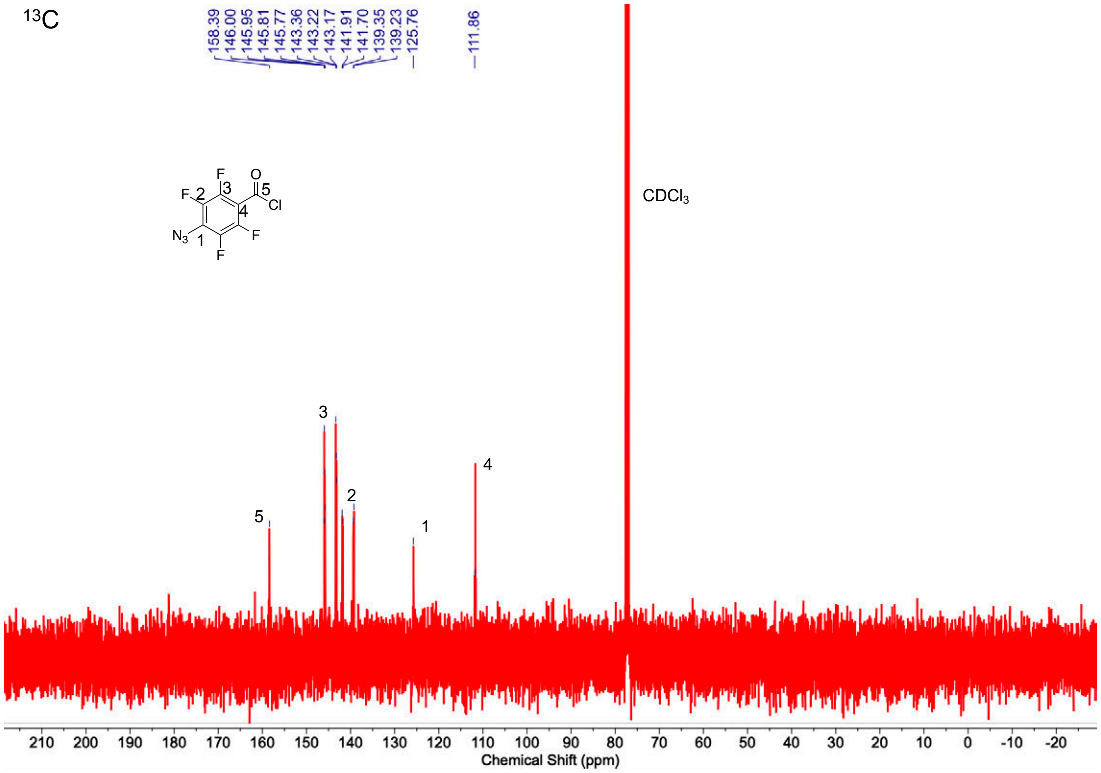

(2) 2,3,4,5,6-Pentafluorobenzoyl chloride

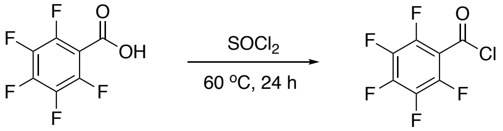

To a 100 mL round-bottom flask was added 6 mL anhydrous thionyl chloride (82 mmol). Under stirring at room temperature, 2 g of 2,3,4,5,6-pentafluorobenzoic acid (9.4 mmol) was added slowly in batches. The suspension was then heated to 65 °C to reflux for 24 h. After the reaction, the residue thionyl chloride was removed under vacuum. The product was cooled down to room temperature and filtered off through a 0.45-μm polytetrafluoroethylene filter to yield a light yellowish liquid. The obtained 2,3,4,5,6-pentafluorobenzoyl chloride used without further purification. Yield: ~90%.

^{19}F-NMR (376 MHz, $CDCl_3$, δ/ppm): -137.73 – -137.85 (2F, m), -144.89 – -145.29 (1F, m), -159.16 – -159.32 (2F, m). ^{13}C-NMR (100 MHz, $CDCl_3$, δ/ppm): 158.36, 145.94−143.22, 143.27, 139.30−136.57, 112.69.

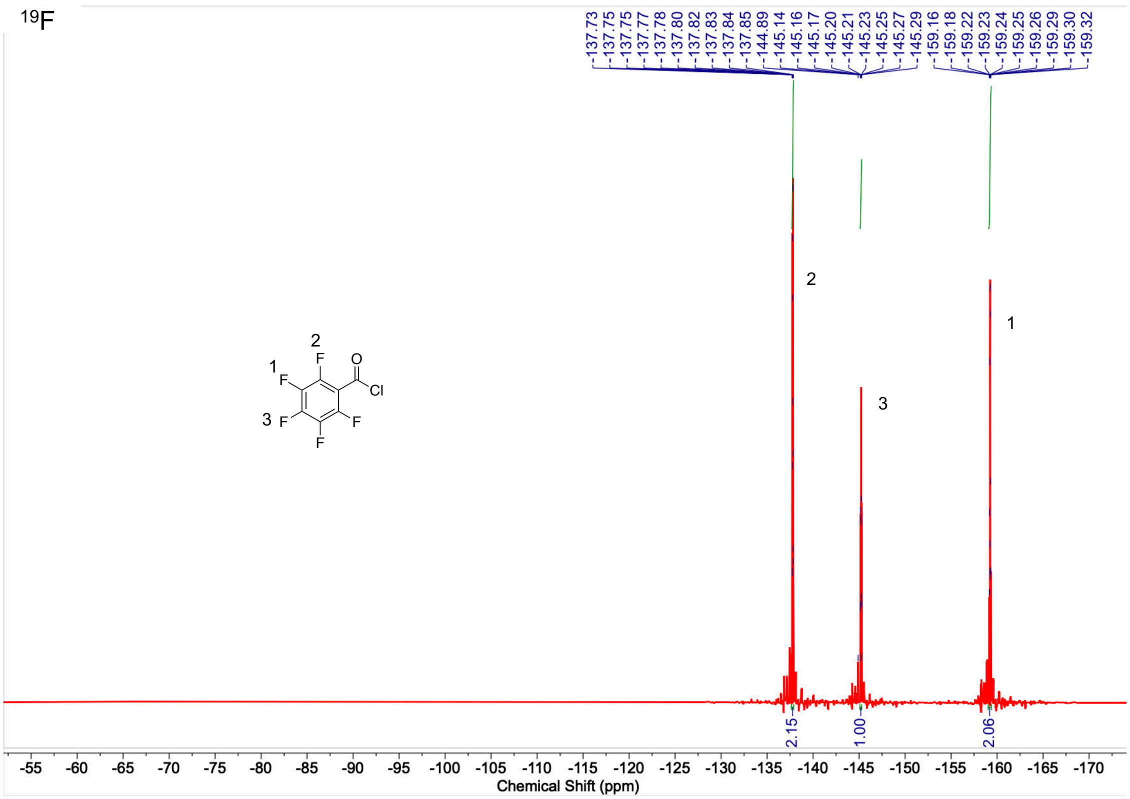
19F
-137.73
-137.75
-137.75
-137.77
-137.78
-137.80
-137.82
-137.83
-137.84
-137.85
-144.89
-145.14
-145.16
-145.17
-145.20
-145.21
-145.23
-145.25
-145.27
-145.29
-159.16
-159.18
-159.22
-159.23
-159.24
-159.25
-159.26
-159.29
-159.30
-159.32
2
1
3
2.15
1.00
2.06
Chemical Shift (ppm)

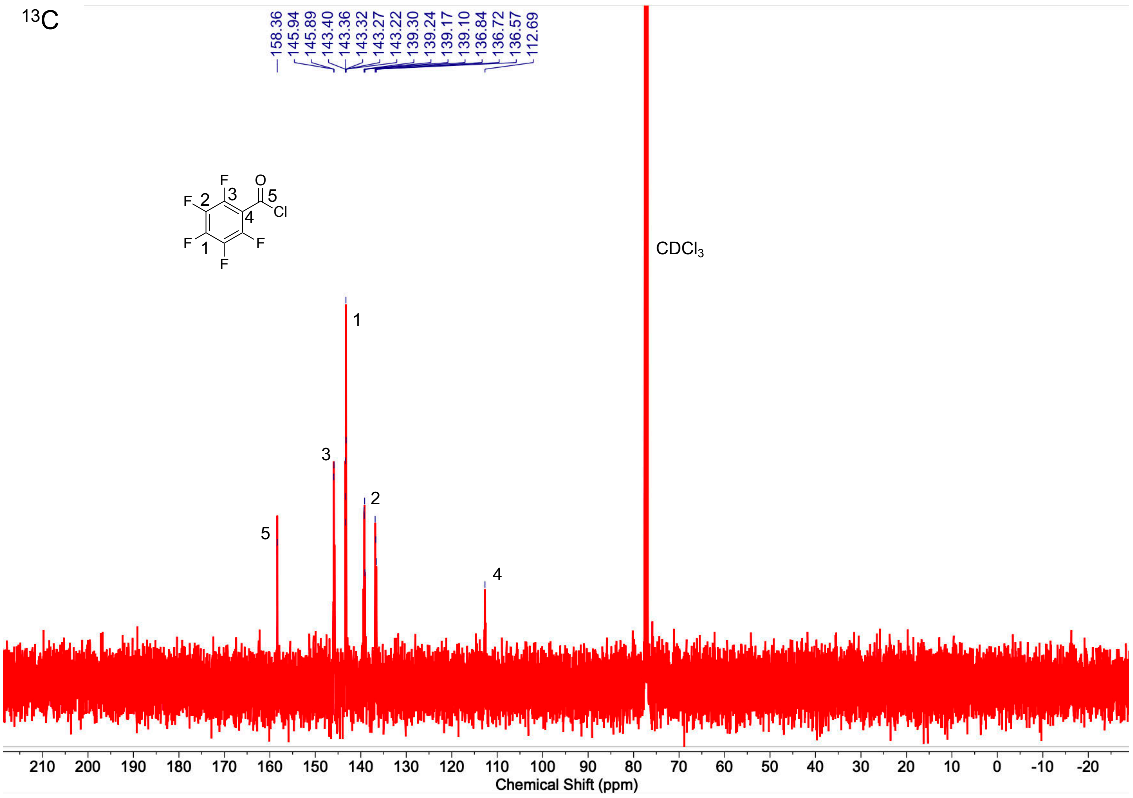
13C
158.36
145.94
145.89
143.40
143.36
143.32
143.27
143.22
139.30
139.24
139.17
139.10
136.84
136.72
136.57
112.69
CDCl3
1
3
2
5
4
Chemical Shift (ppm)

(3) BA

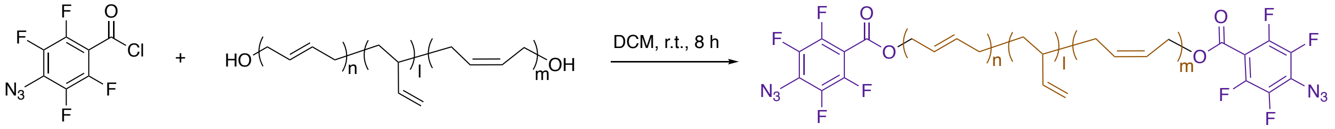

To a 50 mL round-bottom flask were added 10 mL anhydrous dichloromethane (DCM) and 1.2 g hydroxyl-terminated polybutadiene (1 mmol). The mixture was stirred at room temperature for 5 min until homogeneous solution formed. 520 mg 4-azido-2,3,5,6-tetrafluorobenzoyl chloride (2.04 mmol) was first dissolved in 5 mL DCM and the solution was added slowly into the stirring mixture. The solution was then stirred at room temperature for 8 h. After the reaction, the solvent was removed under vacuum (40 ºC for 30 min). The product was cooled down to room temperature and dissolved in chlorobenzene (CB) to form 25 mg mL^{-1} solution for further use (BA easily reacts with itself even below -10 ºC thus it should be kept in dilute solution state). Yield: >99%.

^{1}H-NMR (400 MHz, $CDCl_3$, δ/ppm): 5.66−5.36 (−CH=CH− on backbone, m), 4.99−4.91 (CH_2=CH− on backbone, m), 2.07−2.03 (−CH_2− on backbone, m). ^{19}F-NMR (376 MHz, $CDCl_3$, δ/ppm): -138.09 − -138.19 (4F, m), -150.14 − -150.24 (4F, m). ^{13}C-NMR (100 MHz, $CDCl_3$, δ/ppm): 142.93, 131.48−128.58, 114.55−114.46, 43.78−25.10. ATR-FTIR: ~2123 cm^{-1} (azide), ~1745 cm^{-1} (C=O between azide and backbone).

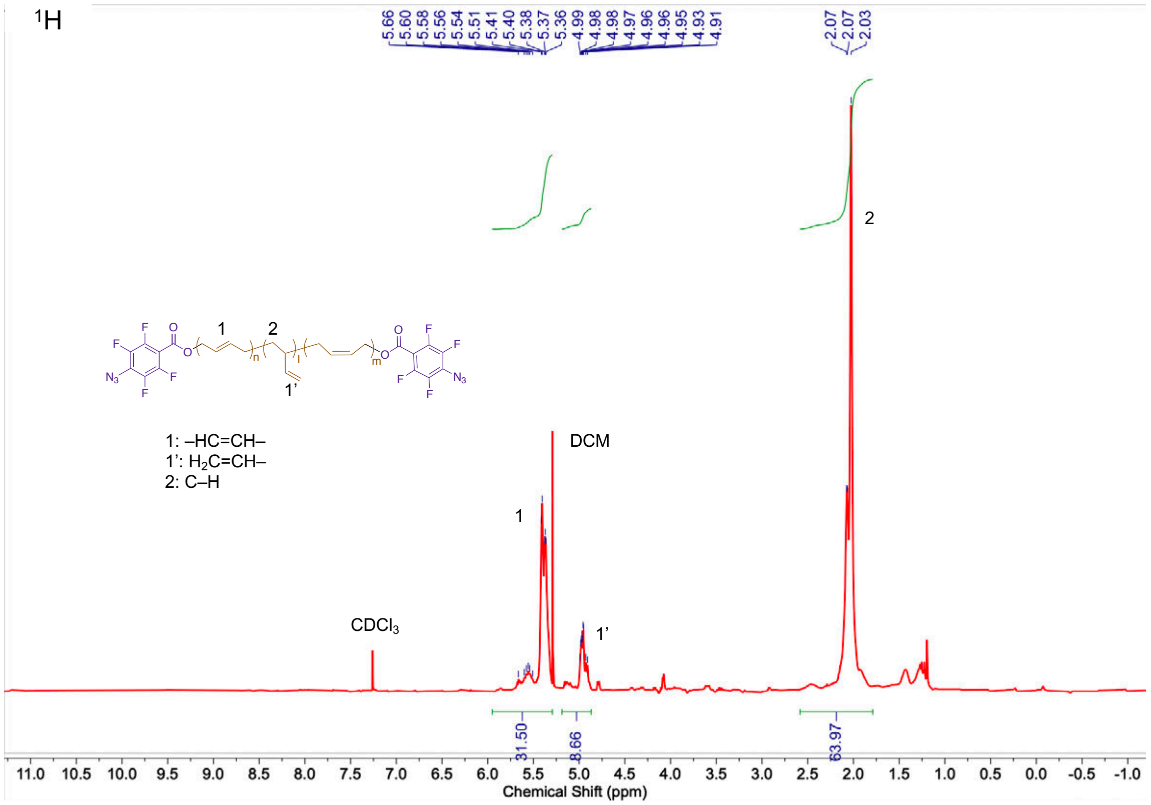

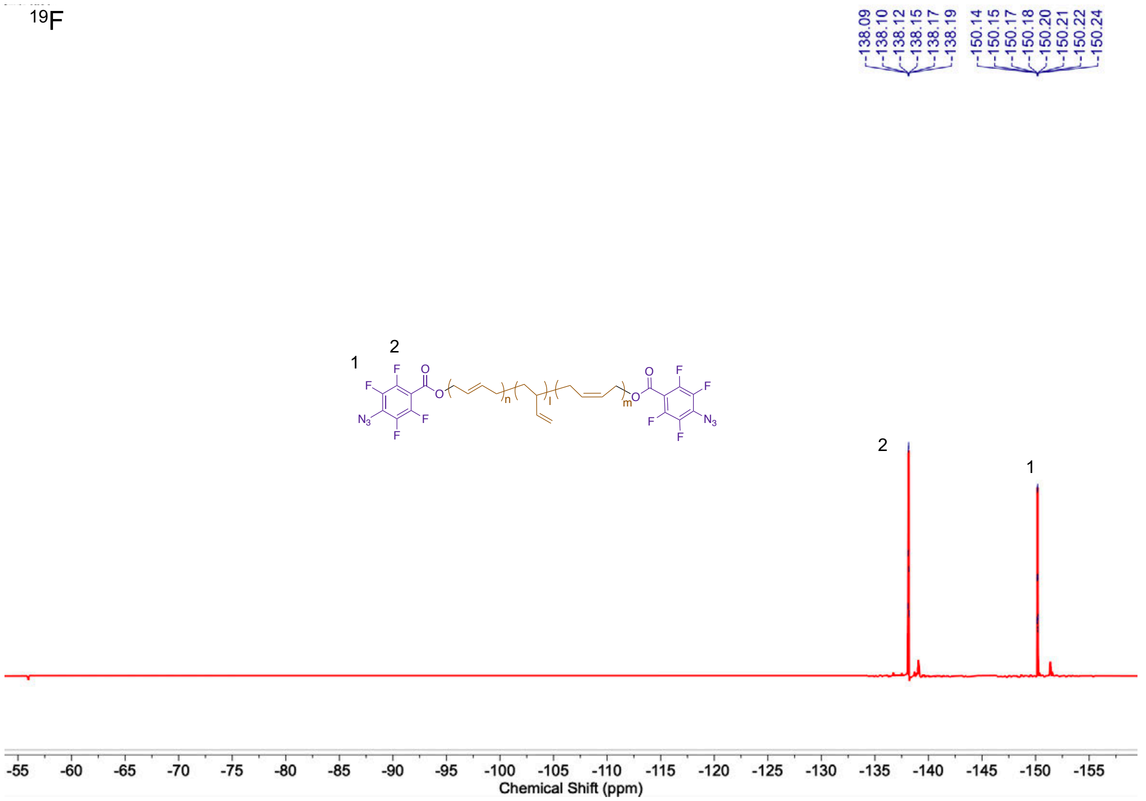
19F
-138.09
-138.10
-138.12
-138.15
-138.17
-138.19
-150.14
-150.15
-150.17
-150.18
-150.20
-150.21
-150.22
-150.24
1
2
N3
2
1
-55 -60 -65 -70 -75 -80 -85 -90 -95 -100 -105 -110 -115 -120 -125 -130 -135 -140 -145 -150 -155
Chemical Shift (ppm)

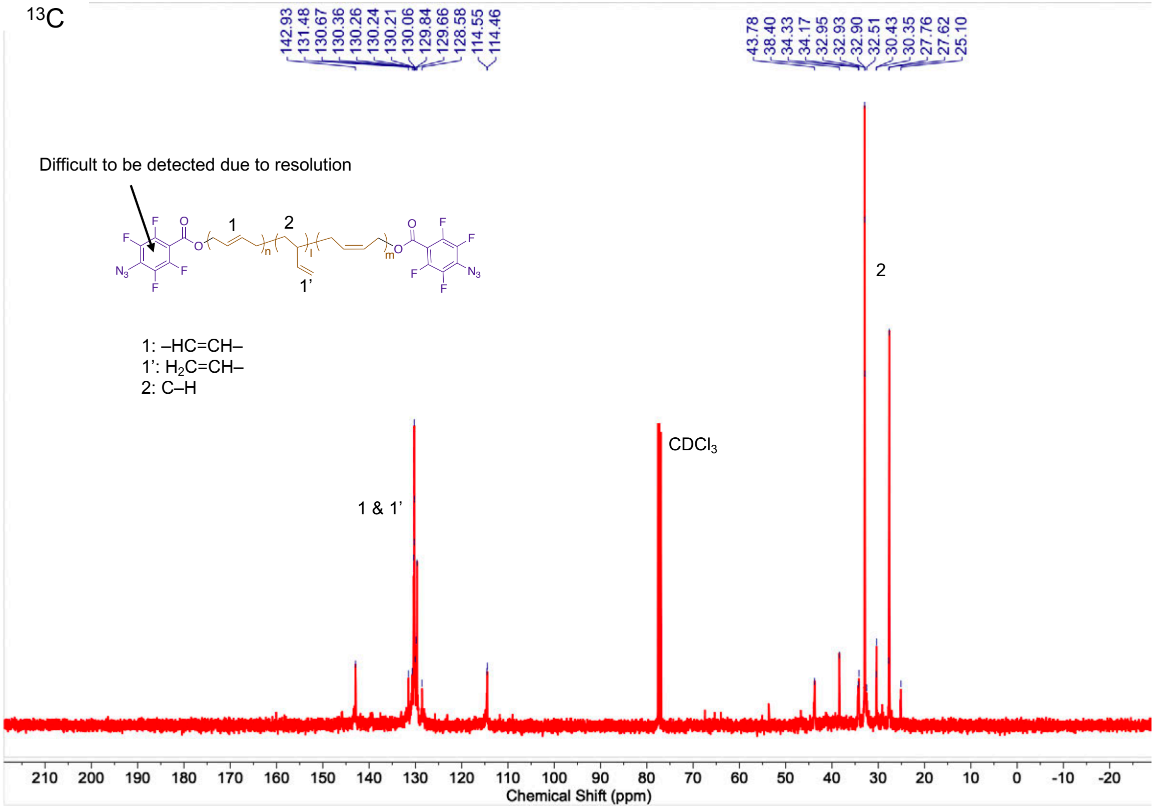
13C
142.93
131.48
130.67
130.36
130.26
130.24
130.21
130.06
129.84
129.66
128.58
114.55
114.46
43.78
38.40
34.33
34.17
32.95
32.93
32.90
32.51
30.43
30.35
27.76
27.62
25.10
Difficult to be detected due to resolution
1
2
1'
1: –HC=CH–
1': H2C=CH–
2: C–H
2
CDCl3
1 & 1'
210 200 190 180 170 160 150 140 130 120 110 100 90 80 70 60 50 40 30 20 10 0 -10 -20
Chemical Shift (ppm)

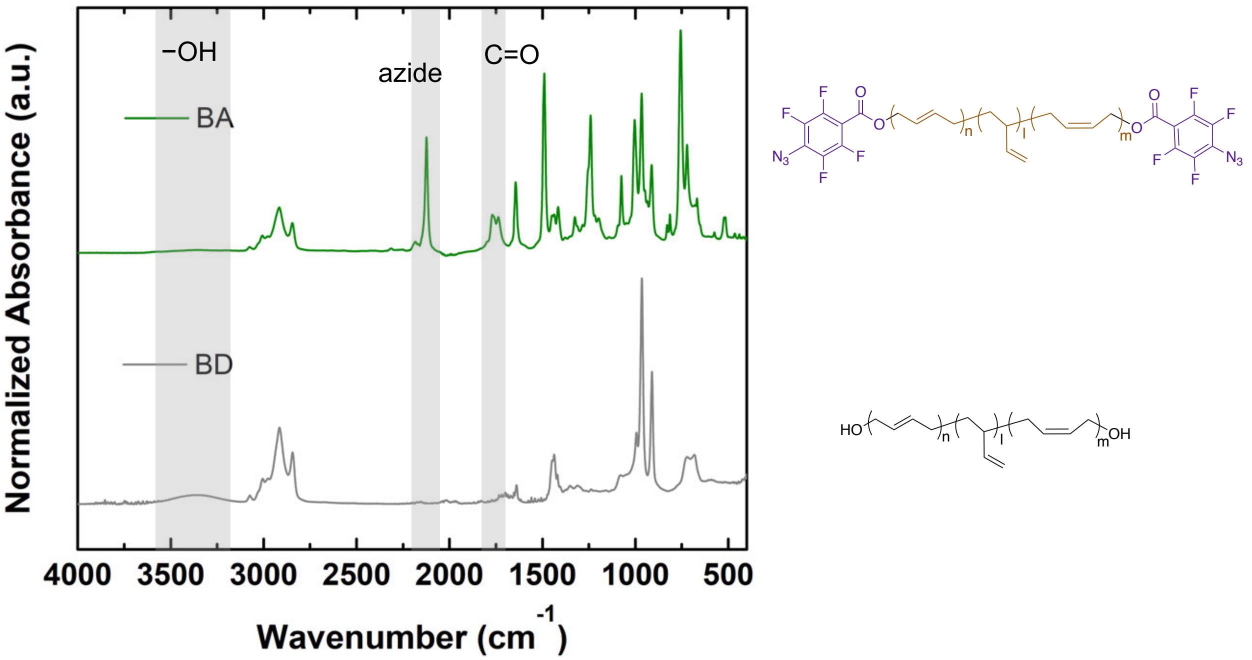

(4) BF

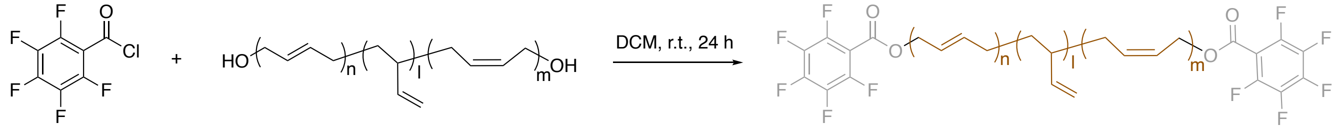

To a 50 mL round-bottom flask were added 10 mL anhydrous DCM and 1.2 g hydroxyl-terminated polybutadiene (1 mmol). The mixture was stirred at room temperature for 5 min until homogeneous solution formed. 475 mg 2,3,4,5,6-pentafluorobenzoyl chloride (2.05 mmol) was first dissolved in 5 mL DCM and the solution was added slowly into the stirring mixture. The solution was then stirred at room temperature for 24 h. After the reaction, the solvent was removed under vacuum (40 °C for 30 min). The product was cooled down to room temperature and dissolved in CB to form 25 mg mL^{-1} solution for further use. Yield: ~99%.

^{1}H-NMR (400 MHz, $CDCl_3$, δ/ppm): 5.56−5.31 (−CH=CH− on backbone, m), 4.98−4.89 (CH_2=CH− on backbone, m), 2.26−2.01 (−CH_2− on backbone, m). ^{19}F-NMR (376 MHz, $CDCl_3$, δ/ppm): -137.26 − -137.38 (4F, m), -144.72 − -144.86 (2F, m), -158.65 − -158.81 (2F, m). ^{13}C-NMR (100 MHz, $CDCl_3$, δ/ppm): 142.93, 130.37−129.67, 114.50, 43.76−25.11. ATR-FTIR: ~1740 cm^{-1} (C=O between azide and backbone).

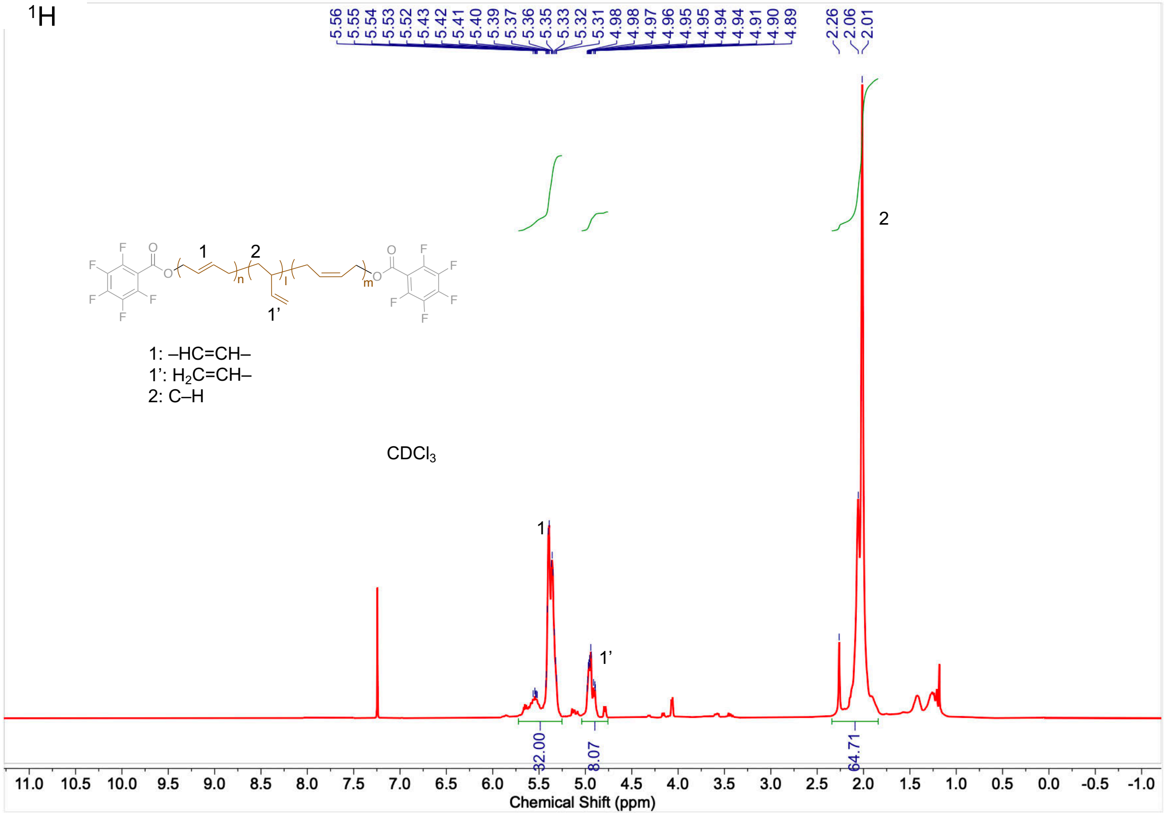

^{1}H
1
2
1'
1: –HC=CH–
1': H_2C=CH–
2: C–H
CDCl$_3$
Chemical Shift (ppm)

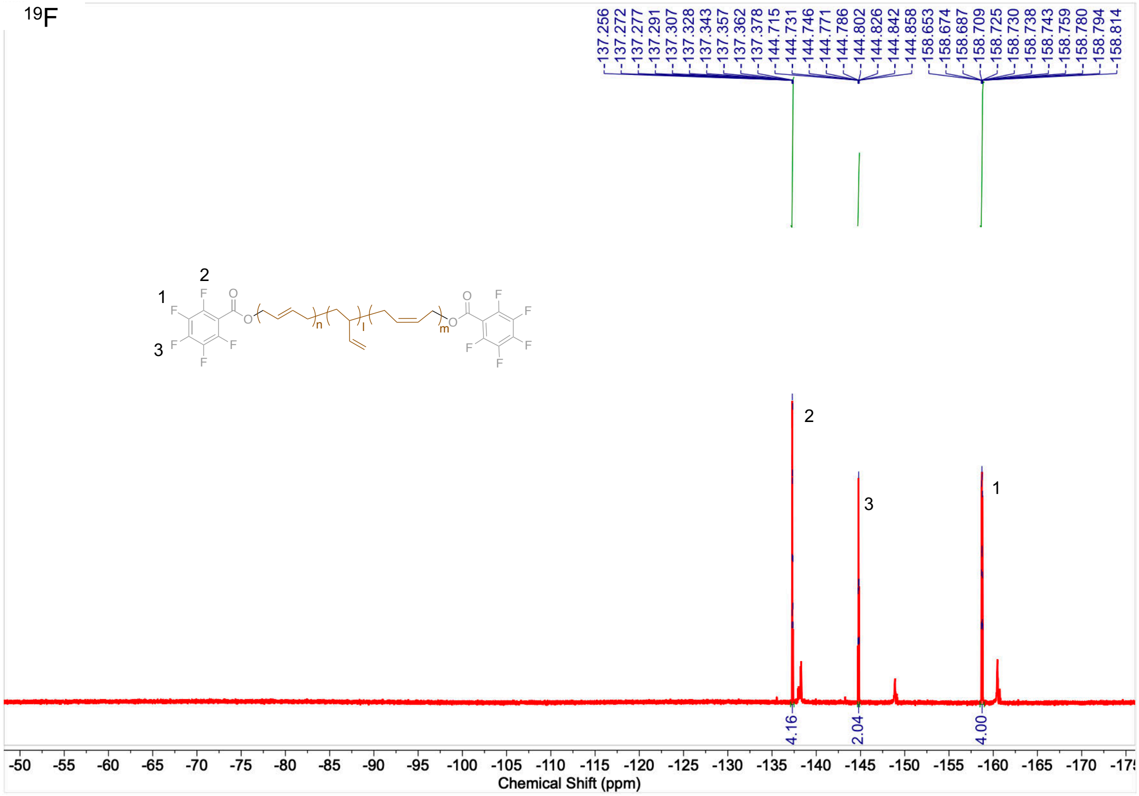

^{19}F
1
2
3
Chemical Shift (ppm)

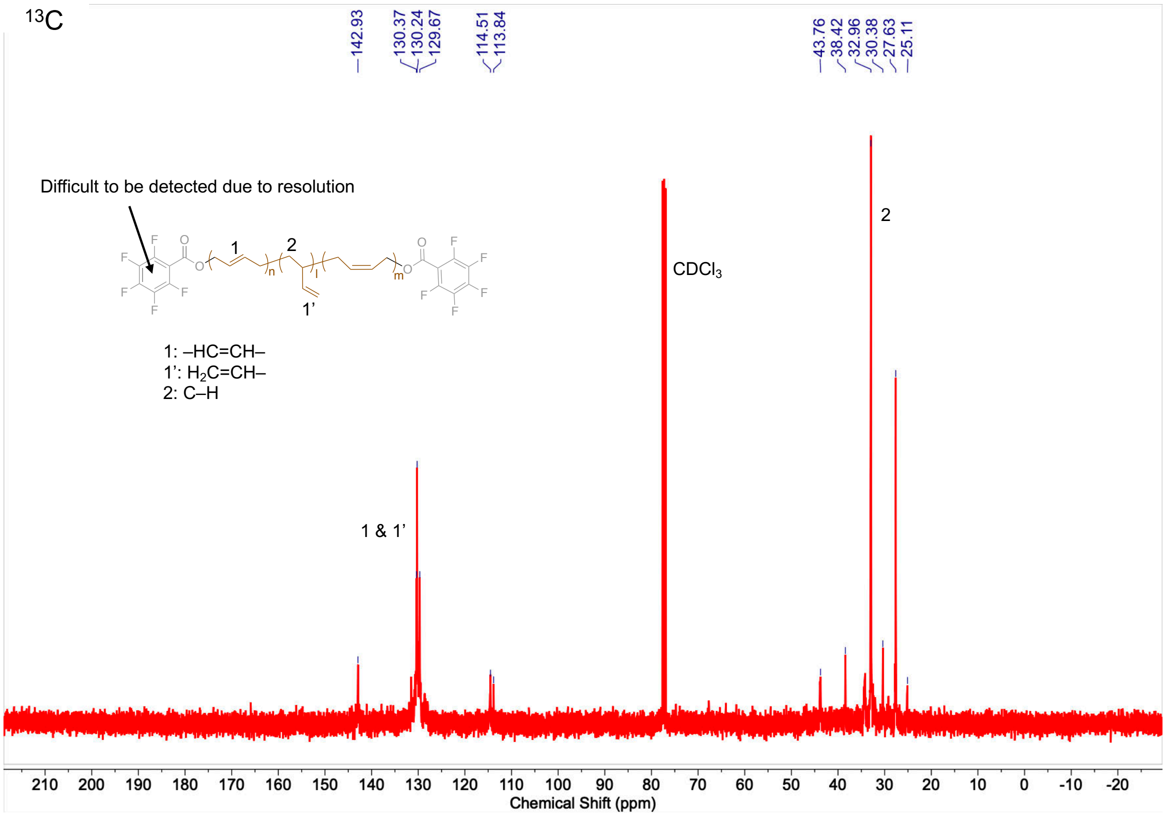

^{13}C
142.93
130.37
130.24
129.67
114.51
113.84
43.76
38.42
32.96
30.38
27.63
25.11
Difficult to be detected due to resolution
1: –HC=CH–
1': H_2C=CH–
2: C–H
1 & 1'
$CDCl_3$
2
Chemical Shift (ppm)

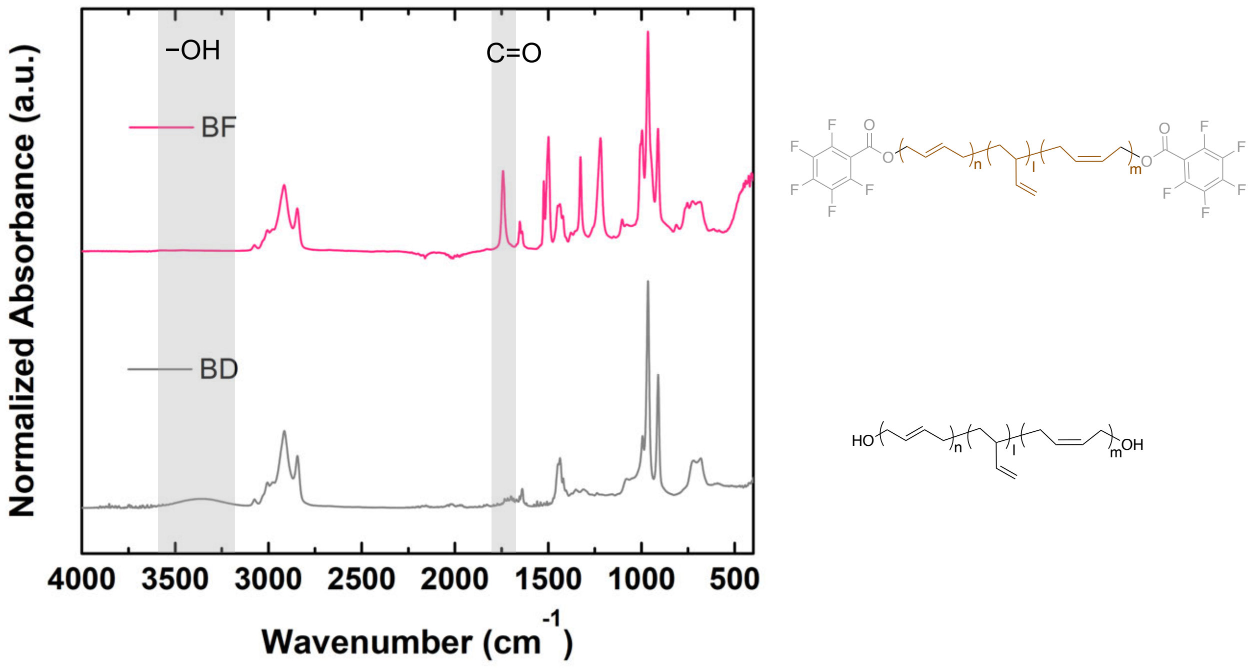

−OH
C=O
BF
BD
Normalized Absorbance (a.u.)
Wavenumber (cm^{-1})

(5) BAc

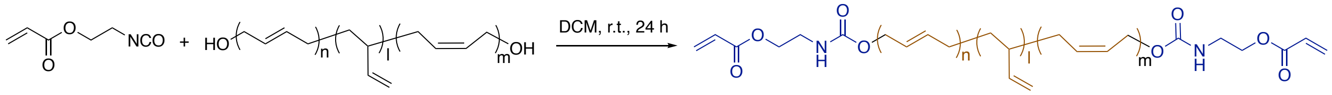

To a 50 mL round-bottom flask were added 10 mL anhydrous DCM and 1.2 g hydroxyl-terminated polybutadiene (1 mmol). The mixture was cooled down to 0 ºC and stirred for 5 min until homogeneous solution formed. At 0 ºC, 290 mg 2-isocyanatoethyl acrylate (2.05 mmol) was first dissolved in 5 mL DCM and was added slowly into the stirring mixture. The solution was then allowed to warm up to room temperature and stirred for 24 h. After the reaction, the solvent was removed under vacuum (40 ºC for 30 min). The product was cooled down to room temperature and dissolved in CB to form 25 mg mL^{-1} solution for further use (BAc can be kept by itself at -10 ºC). Yield: ~99%.

^{1}H-NMR (400 MHz, $CDCl_3$, δ/ppm): 6.50−5.84 (6H on CH_2=CH− of acrylate, m), 5.59−5.36 (−CH=CH− on backbone, m), 4.99−4.90 (CH_2=CH− on backbone, m), 4.24−4.22 (4H on $-CH_2-$ of acrylate, m), 3.49−3.48 (4H on $-CH_2-$ of acrylate, m), 2.07−2.02 ($-CH_2-$ on backbone, m). ^{13}C-NMR (100 MHz, $CDCl_3$, δ/ppm): 166.24, 142.90, 131.64−128.19, 114.48, 63.83, 43.78−27.62. ATR-FTIR: ~1723 cm^{-1} (C=O between azide and backbone).

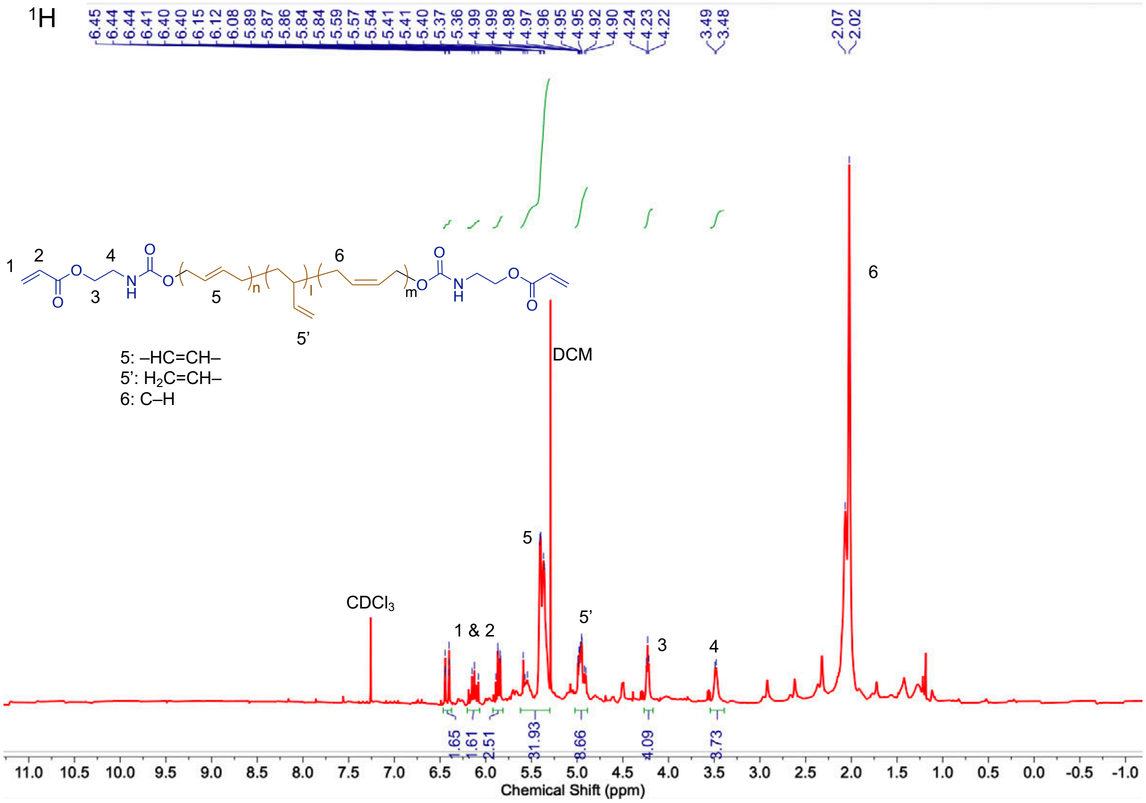

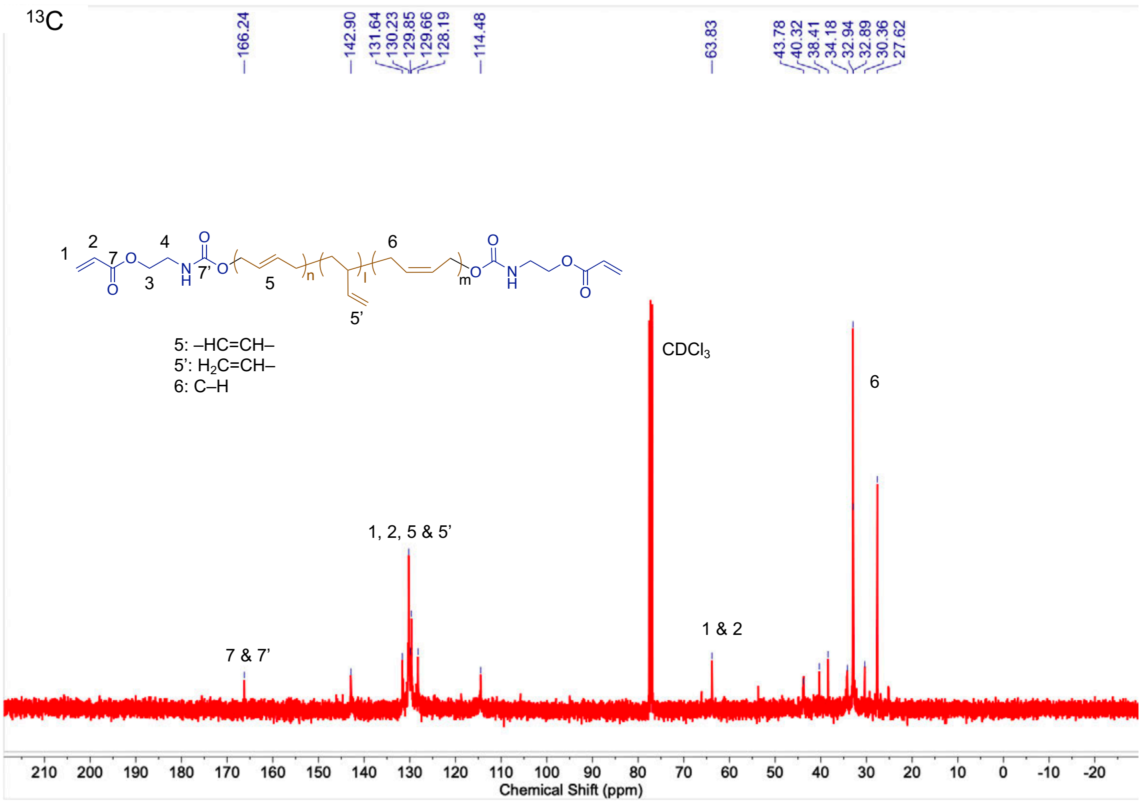

13C
166.24
142.90
131.64
130.23
129.85
129.66
128.19
114.48
63.83
43.78
40.32
38.41
34.18
32.94
32.89
30.36
27.62
5: –HC=CH–
5': H2C=CH–
6: C–H
CDCl3
6
1, 2, 5 & 5'
1 & 2
7 & 7'
Chemical Shift (ppm)

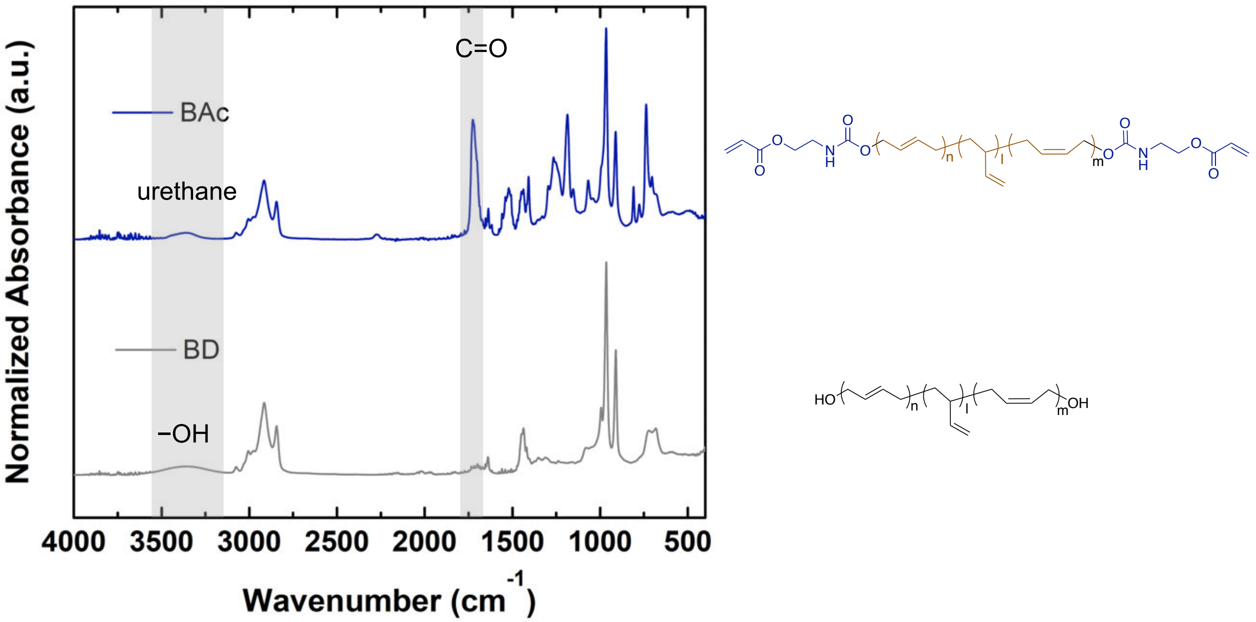

C=O
BAc
urethane
BD
–OH
Normalized Absorbance (a.u.)
Wavenumber (cm-1)

(6) Hydrogenated hydroxyl-terminated polybutadiene

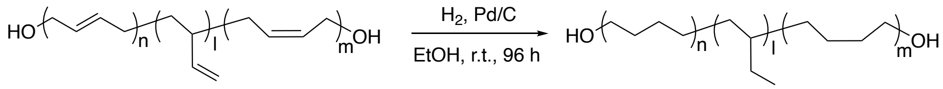

To a 250 mL double-neck round-bottom flask were added 100 mL anhydrous ethyl acetate (EA) and 4.8 g hydroxyl-terminated polybutadiene (4 mmol). The mixture was degassed by N_2 and stirred for 30 min until homogeneous emulsion formed. Then 500 mg Pd/C (dry, type 487) was added to form suspension and the N_2 atmosphere was purged with H_2 balloons. The solution was stirred at room temperature for 96 h during which H_2 balloons were frequently refilled to ensure enough H_2 pressure. After the reaction, the suspension was filtered off and the solvent of obtained solution was removed under vacuum (50 °C for 1 h). The product was cooled down to room temperature to yield white, non-flowable wax hydrogenated hydroxyl-terminated polybutadiene. Yield: ~50%.

^{1}H-NMR (400 MHz, $CDCl_3$, δ/ppm): 1.24−1.20 (C−H on backbone, m). ^{13}C-NMR (100 MHz, $CDCl_3$, δ/ppm): 39.05−25.99 (alkyl C on backbone, m).

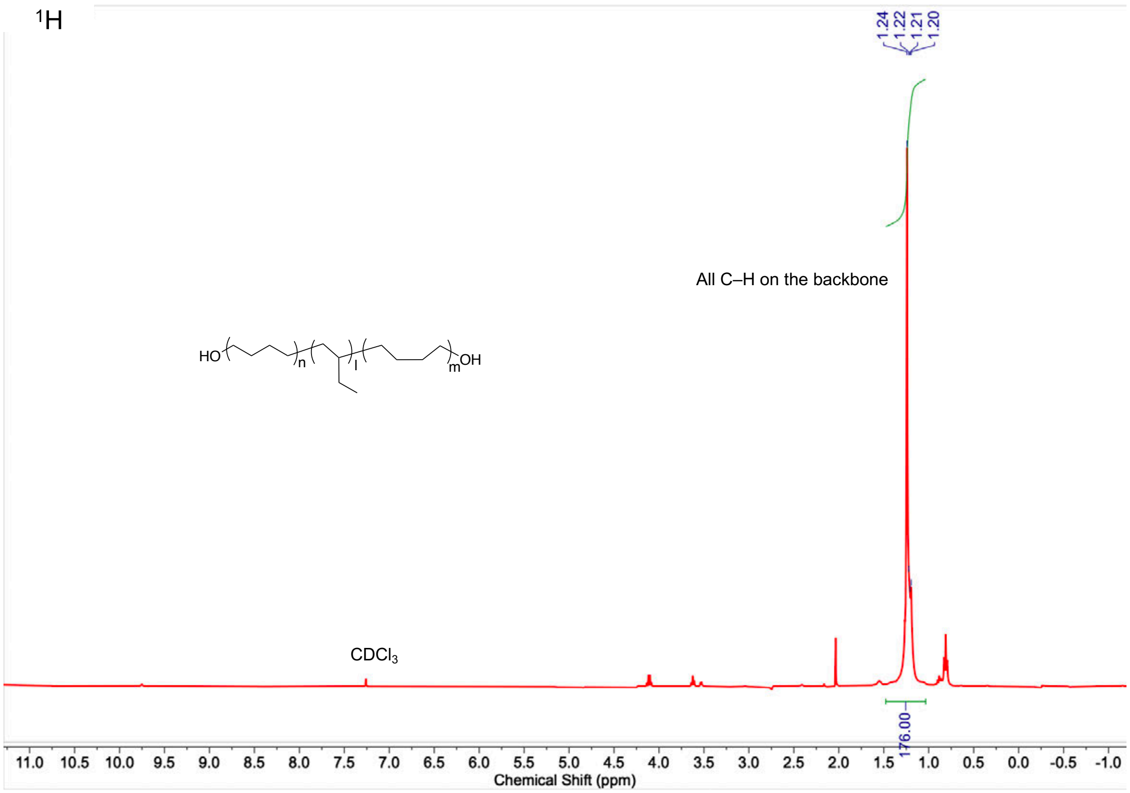

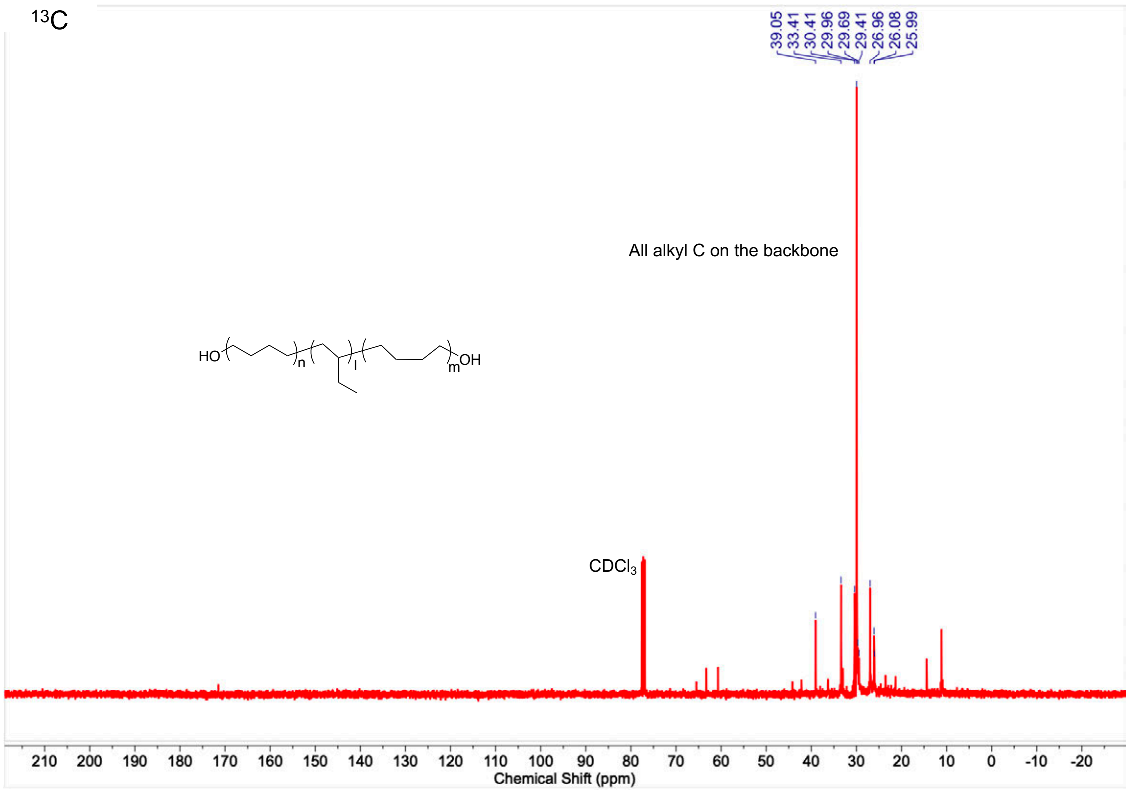

(7) BH

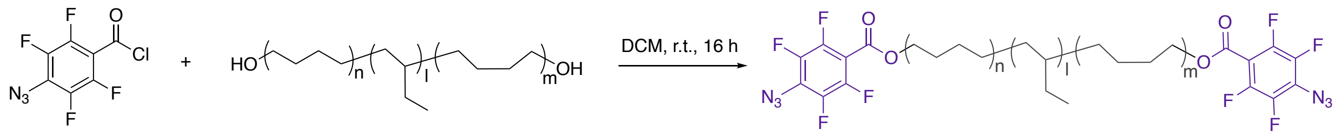

To a 50 mL round-bottom flask were added 10 mL anhydrous DCM and 620 mg hydrogenated hydroxyl-terminated polybutadiene (0.5 mmol). The mixture was stirred at room temperature for 5 min until homogeneous solution formed. 260 mg 4-azido-2,3,5,6-tetrafluorobenzoyl chloride (1.02 mmol) was first dissolved in 5 mL DCM and the solution was added slowly into the stirring mixture. The solution was then stirred at room temperature for 16 h. After the reaction, the solvent was removed under vacuum (40 ºC for 30 min). The product was cooled down to room temperature and dissolved in CB to form 100 mg mL^{-1} solution for further use. (BH is not as reactive as BA due to the lack of double bonds but it would be better to be kept in dilute solution state) Yield: ~99%.

^{1}H-NMR (400 MHz, $CDCl_3$, δ/ppm): 1.26−1.21 (C−H on backbone, m). ^{19}F-NMR (376 MHz, $CDCl_3$, δ/ppm): -138.11 − -138.21 (4F, m), -150.16 − -150.26 (4F, m). ^{13}C-NMR (100 MHz, $CDCl_3$, δ/ppm): 39.07−26.10 (alkyl C on backbone, m). ATR-FTIR: ~2126 cm^{-1} (azide), ~1734 cm^{-1} (C=O between azide and backbone).

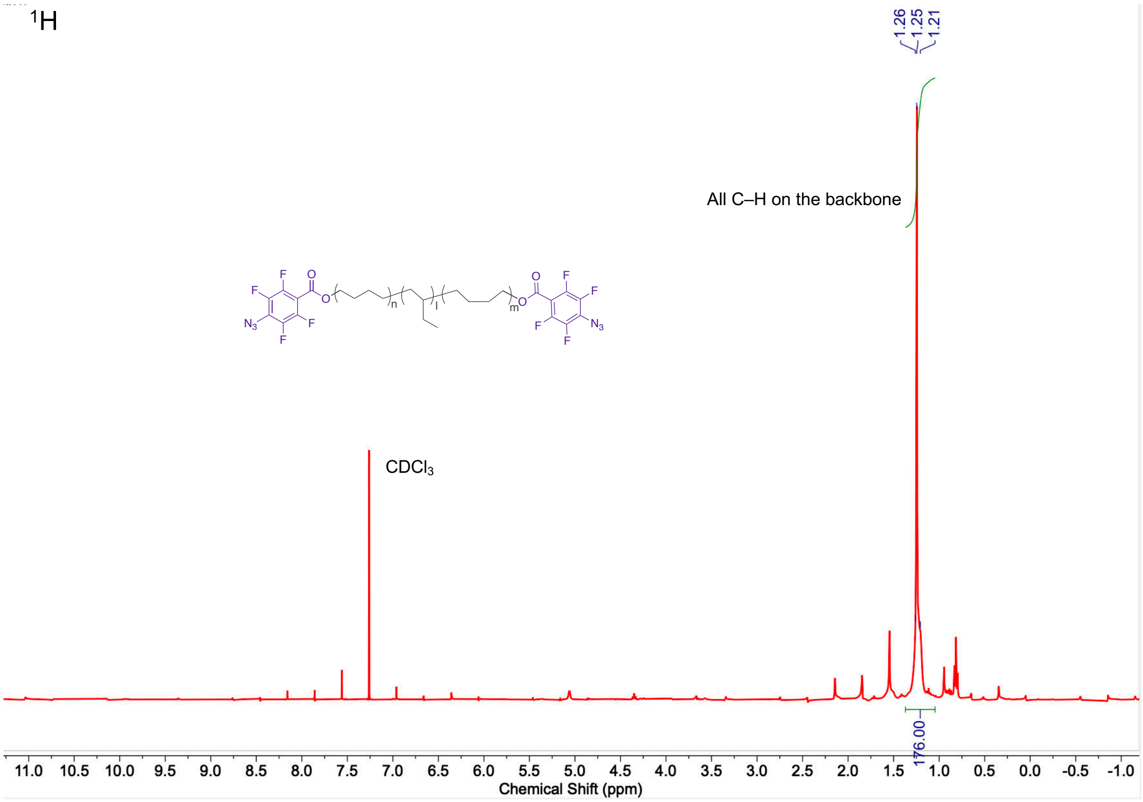

1H
1.26
1.25
1.21
All C–H on the backbone
CDCl3
176.00
11.0 10.5 10.0 9.5 9.0 8.5 8.0 7.5 7.0 6.5 6.0 5.5 5.0 4.5 4.0 3.5 3.0 2.5 2.0 1.5 1.0 0.5 0.0 -0.5 -1.0
Chemical Shift (ppm)

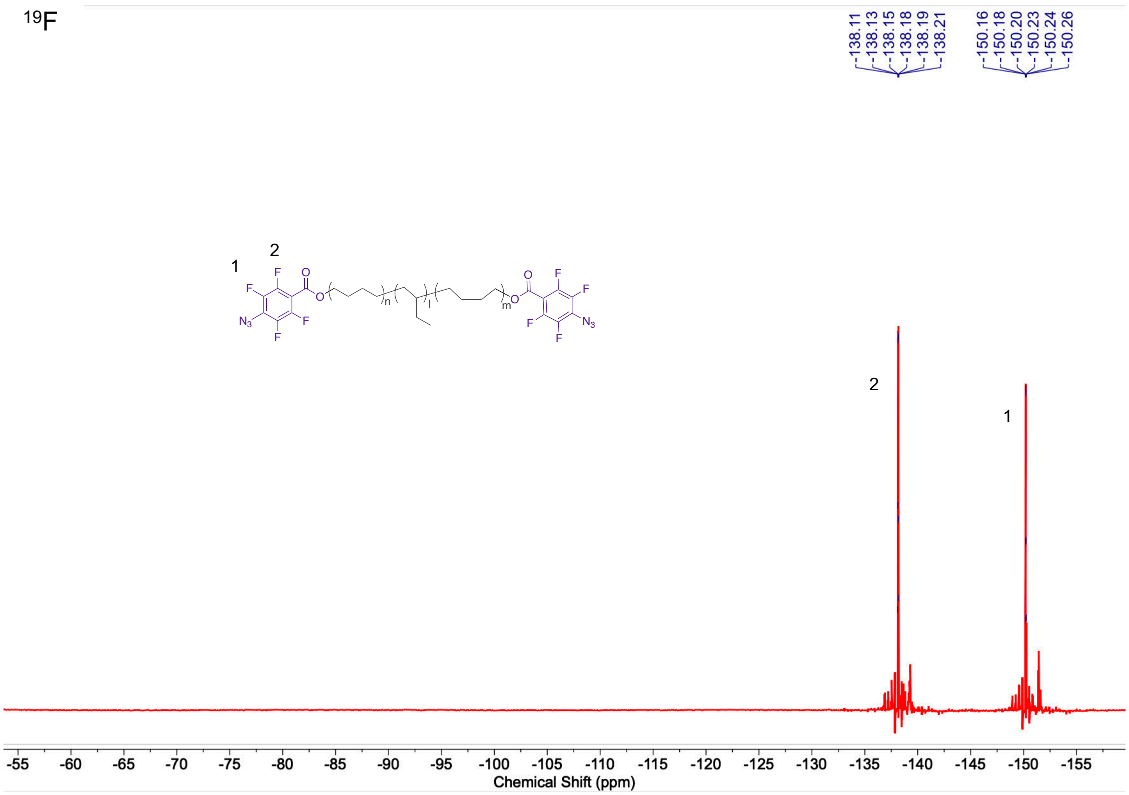

19F
-138.11
-138.13
-138.15
-138.18
-138.19
-138.21
-150.16
-150.18
-150.20
-150.23
-150.24
-150.26
1
2
-55 -60 -65 -70 -75 -80 -85 -90 -95 -100 -105 -110 -115 -120 -125 -130 -135 -140 -145 -150 -155
Chemical Shift (ppm)

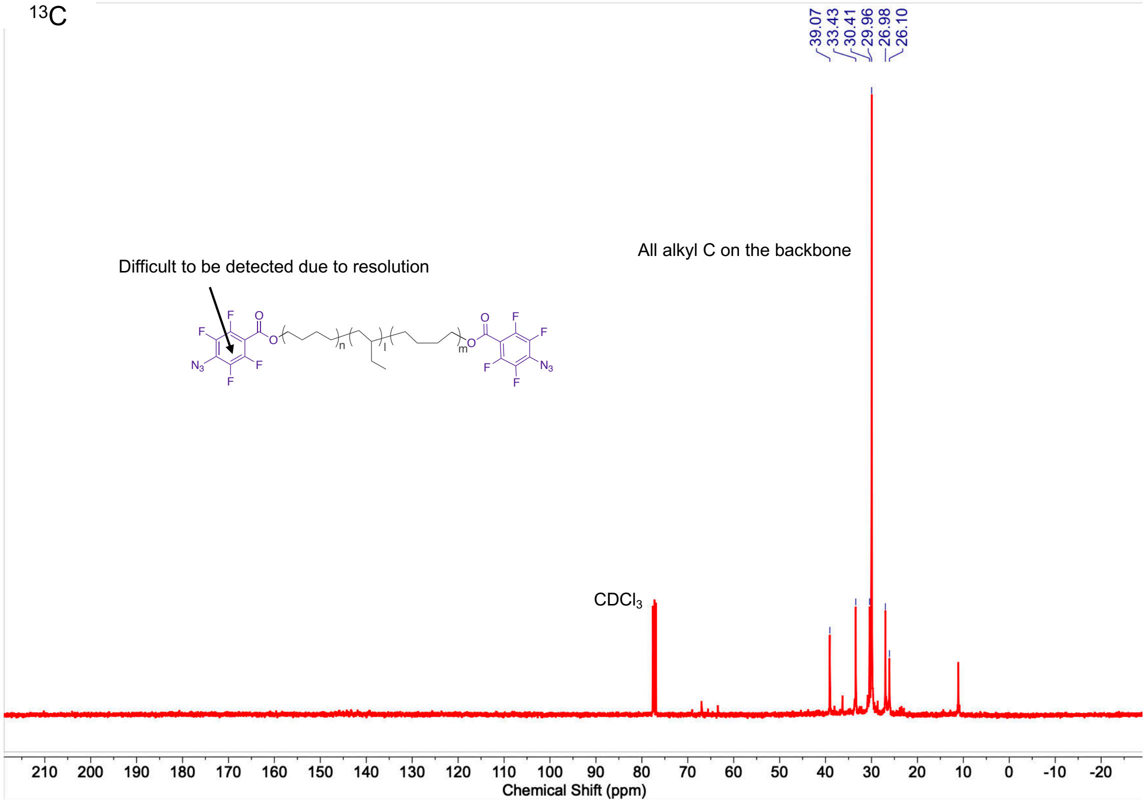

13C
39.07
33.43
30.41
29.96
26.98
26.10
Difficult to be detected due to resolution
All alkyl C on the backbone
CDCl3
210 200 190 180 170 160 150 140 130 120 110 100 90 80 70 60 50 40 30 20 10 0 -10 -20
Chemical Shift (ppm)

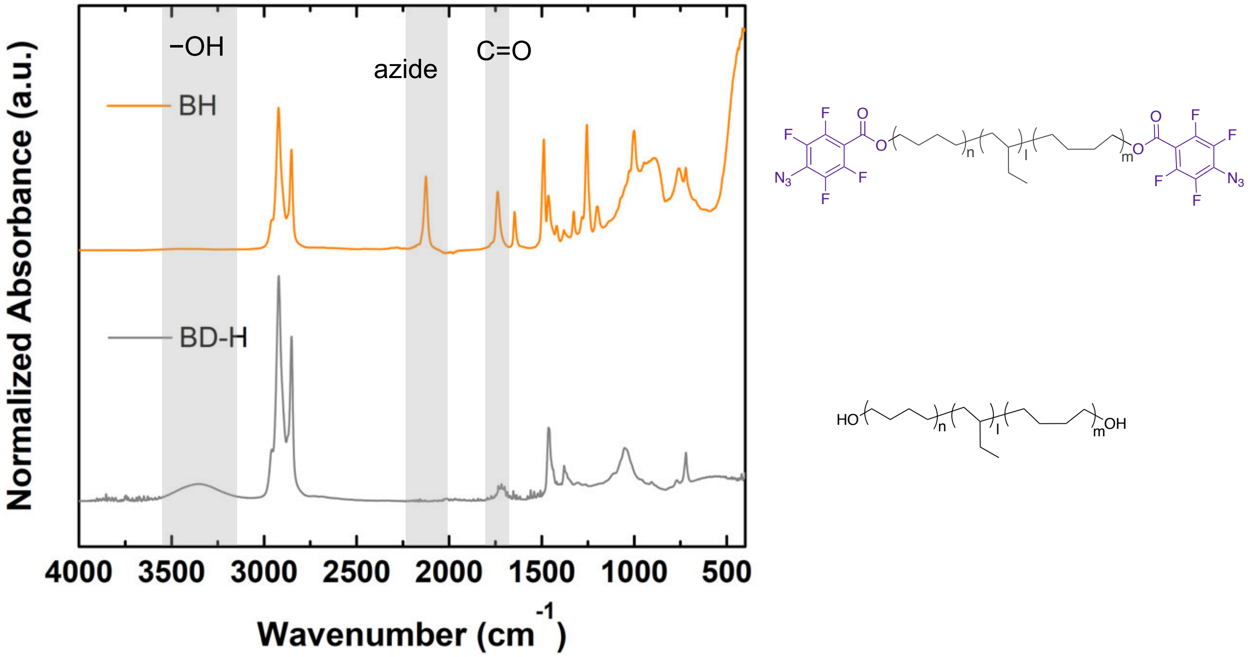

−OH
azide
C=O
BH
BD-H
Normalized Absorbance (a.u.)
4000 3500 3000 2500 2000 1500 1000 500
Wavenumber (cm-1)

2. Supplementary Figures 1-68, Tables 1-6, Notes 1-2, and Movie 1

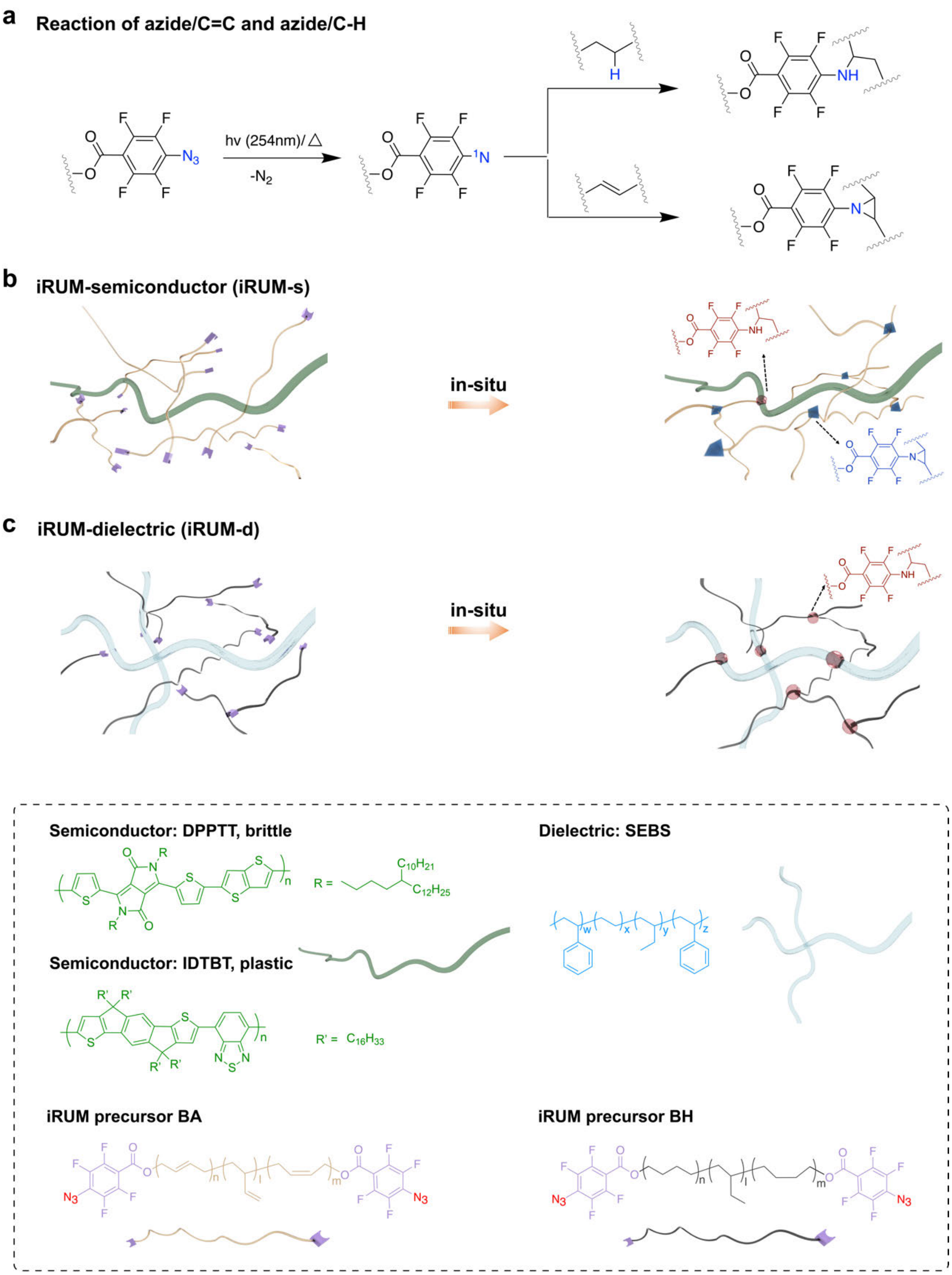

Supplementary Figure 1. (a) Perfluorophenyl azide (PFPA) crosslinking reaction with C=C or C-H. (b,c) In-situ rubber formation processes of iRUM-s (b) and iRUM-d (c).

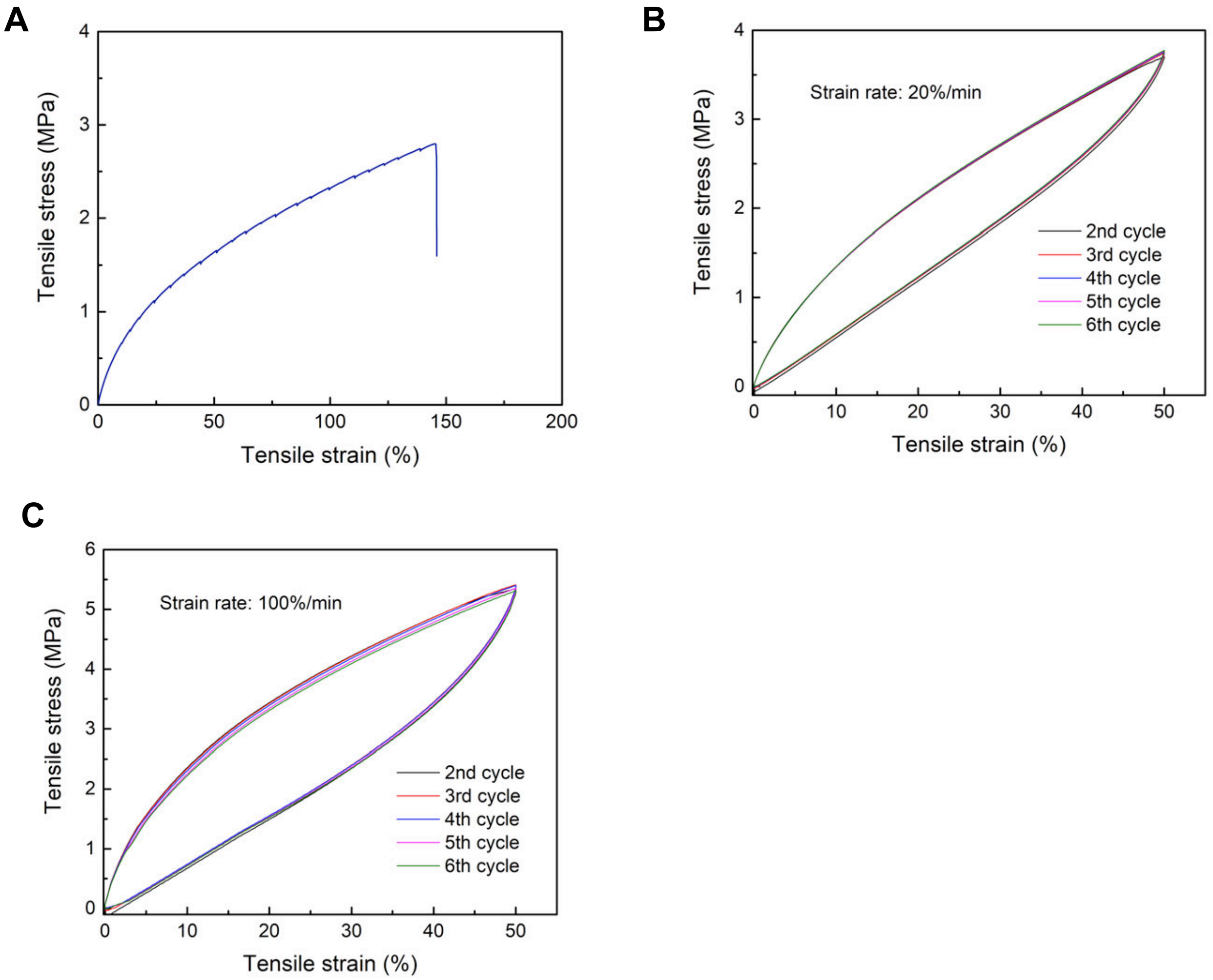

Supplementary Figure 2. Characterizations of mechanical properties for a crosslinked free-standing BA rubber film prepared by heating in vacuum. (**A**) stress-strain curve under single loading with the strain rate of 100%/min. Film dimension: length 2mm, width 2.7mm, thickness 0.8mm. The extracted Young's modulus is 6.86 MPa, while the strain at break is 145.85%. Cyclic stress-strain curves with the strain rate of (**B**) 20%/min and (**C**)100%/min. Film dimension: length 1.7mm, width 2.7mm, thickness 0.8mm. There is no residue strain after multiple loading-unloading cycles and the stress-strain curve at different cycle overlap with each other very well, indicating the elastic behavior with no strain-rate dependence. The first cycle is omitted due to the clamp sliding.

Supplementary Note 1. The rationale for good mixing.

The well blending of polybutadiene-derived precursors with conjugated polymers is the premise to enable our iRUM approach. This allows uniform crosslinking and prevents large-domain phase separation, which may result in stress concentration at domain boundaries and more likely to have mechanical failure under strain.

As confirmed by AFM and IR-AFM of BF/DPPTT-x:y blend films, no micrometer-sized domains due to crystallization was observed at different BF ratios. The high flexibility of polybutadiene and matched surface energy with semiconducting polymer contributes to their uniform mixing with conjugated polymer nanostructures, which are essential for high charge carrier mobility. However, in some small molecule/conjugated polymer blend systems, the small molecules have strong tendencies to crystallize, thus making large non-uniform phase separation more favorable.

BF/DPPTT was used as an analog to mimic the non-crosslinked blend and it showed similar morphology to the crosslinked one (iRUM-s-x:y, BAc/DPPTT-x:y and BH/DPPTT-x:y crosslinked films). The uniform distribution of DPPTT nanostructures was still maintained. Therefore, we assumed that the morphology of composite film was mainly determined during solution mixing and subsequent solution deposition (spin coating), which was not affected too much by crosslinking process. Furthermore, the low surface roughness of iRUM-s films is also beneficial when incorporated into electronic devices where the charge transport is across or along interfaces between different device components.

**BA/DPPTT** (wt/wt)	1:9	3:7	1:1	3:1
Crosslinking density (10^{-4} mol/g)	1.2	3.6	6	9

Supplementary Table 1. Crosslinking density of iRUM-s films. The crosslinking density is calculated by using the molar percentage of azide over the total weight of composite film.

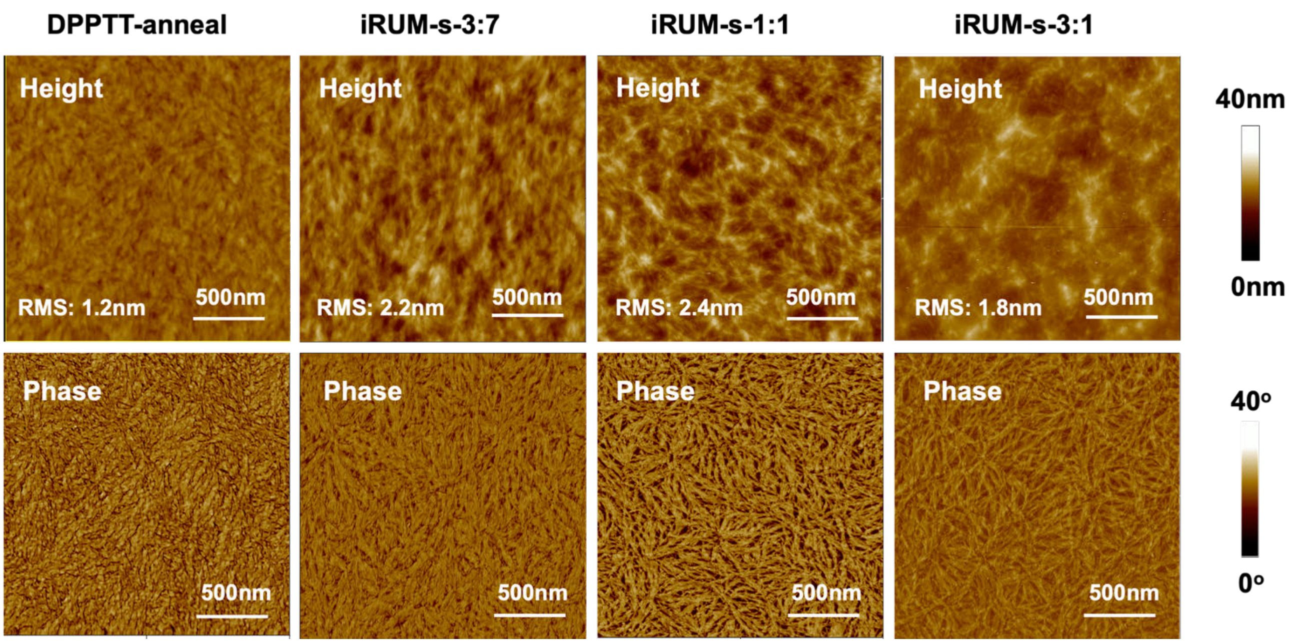

Supplementary Figure 3. AFM height and phase images of DPPTT film annealed at 150 °C for 40 min and iRUM-s-x:y films, where x:y is the BA-to-DPPTT weight ratio. The surface roughness is obtained from AFM height image.

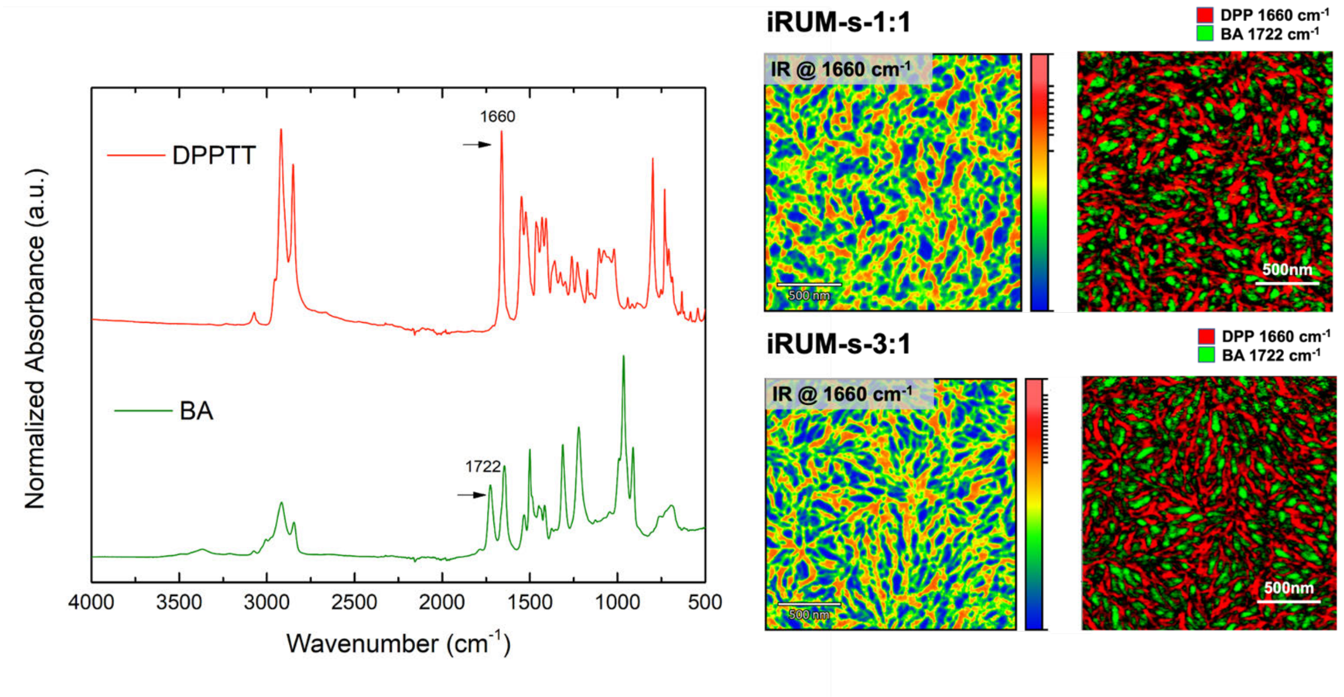

Supplementary Figure 4. IR absorption spectrum of DPPTT and crosslinked BA taken by FT-IR. The non-overlapping characteristic peaks selected for DPPTT and BA are 1660 and 1722 cm^{-1}, respectively. IR-AFM overlay images (composition map) highlighting the distributions of DPPTT and BA in iRUM-s-x:y films (30-40 nm). The IR-AFM probes the nanoscale topography and local chemical composition of film surface.

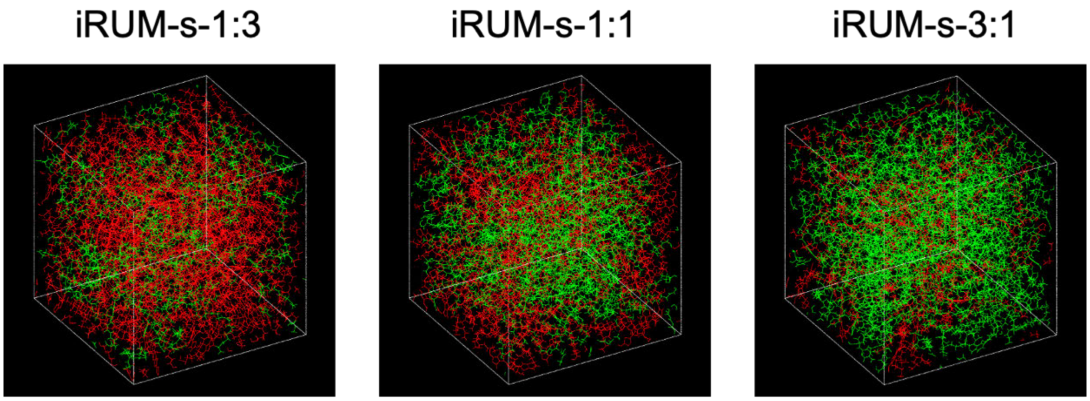

iRUM-s-x:y	Chain number BA	Chain number DPPTT_5
1:3	19	15
1:1	37	10
3:1	56	5

Supplementary Figure 5. Screen shot of MD simulated iRUM-s-x:y films and the corresponding molecule numbers used in the simulations. The red color represents DPPTT, while the green color represents BA. Box size: 5.8-5.9 nm. The number of BA and DPPTT chains in the simulation box for different iRUm-s-x:y films is referred to the table below. This simulation result suggests uniform mixing of BA and DPPTT in a blend.

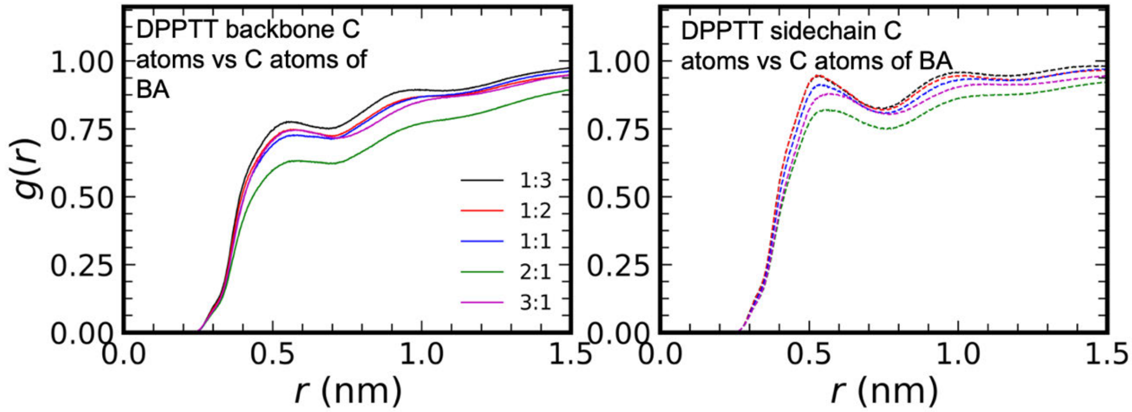

Supplementary Figure 6. Radial distribution function (RDF) of BA/DPPTT-x:y blends extracted from MD simulations. The ratio numbers (r) in the figure legend represent the weight ratios (x:y). The corresponding chain number ratios can be obtained from the above MD simulations section.

Note: The association between DPPTT sidechain and BA is stronger than the association between DPPTT backbone and BA. As BA concentration increases, the association between BA with both DPPTT backbone and side chain decreases, which is due to the increased association among BA molecules.

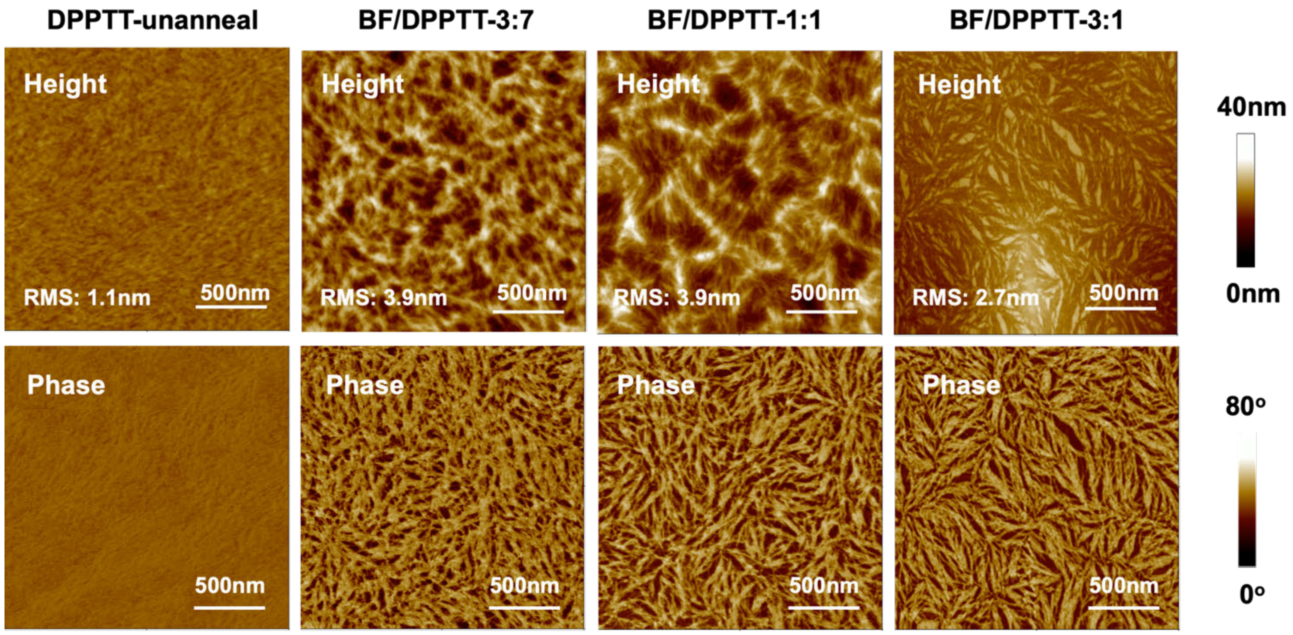

Supplementary Figure 7. AFM height and phase images of DPPTT unannealed film and BF/DPPTT-x:y blend films, where x:y is the BF-to-DPPTT weight ratio. The surface roughness is obtained from AFM height image. Note: DPPTT+BF was used as an analog to mimic the non-crosslinked blend and it showed similar morphology to the crosslinked one, DPPTT+BA. Therefore, we assumed that the morphology of composite film was mainly determined during solution mixing and subsequent solution deposition (spin coating).

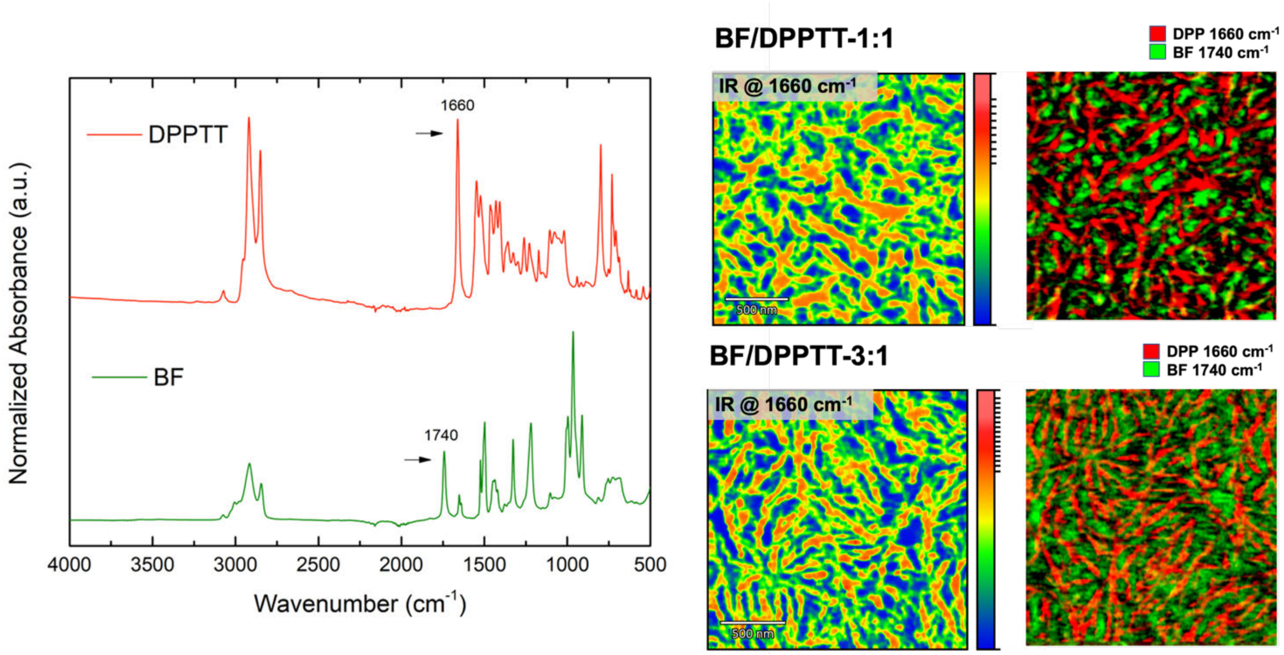

Supplementary Figure 8. IR absorption spectrum of DPPTT and BF taken by FT-IR. The characteristic peaks selected for DPPTT and BF are 1660 and 1740 cm^{-1}, respectively. IR-AFM overlay images (composition map) highlighting the distributions of DPPTT and BF in BF/DPPTT-x:y blend films.

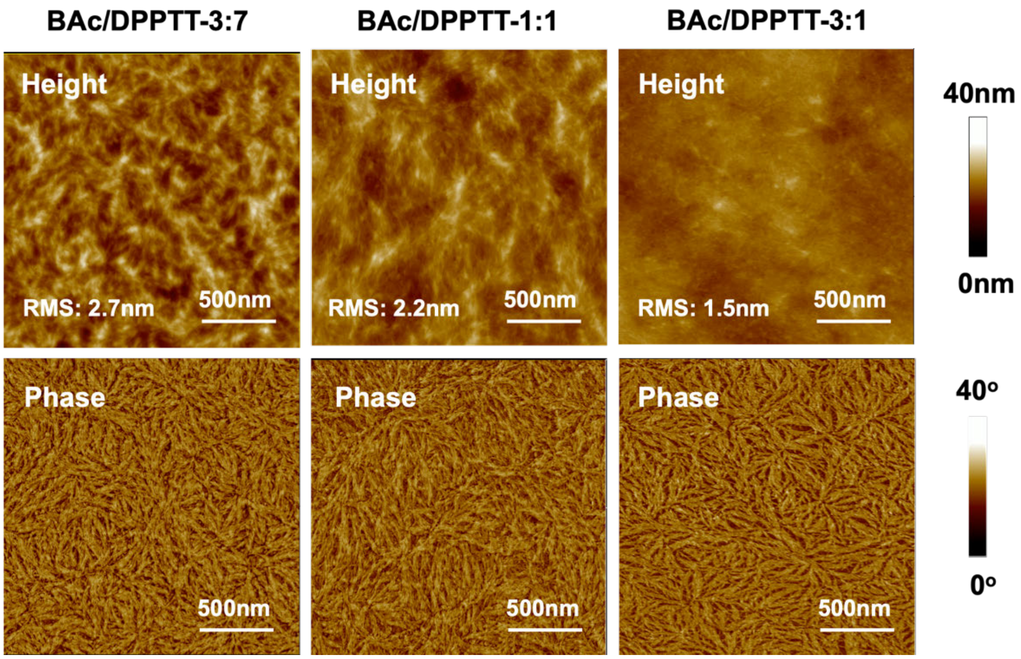

Supplementary Figure 9. AFM height and phase images of BAc/DPPTT-x:y crosslinked films, where x:y is the BAc-to-DPPTT weight ratio. The surface roughness is obtained from AFM height image.

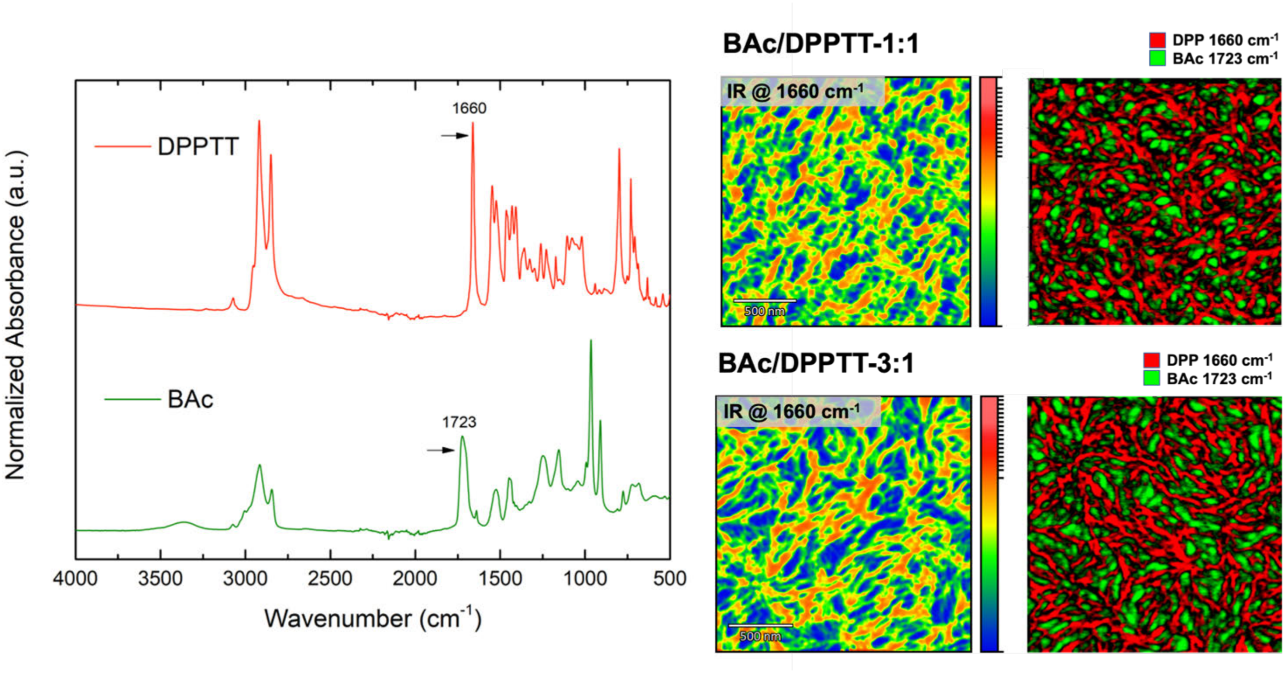

Supplementary Figure 10. IR absorption spectrum of DPPTT and crosslinked BAc taken by FT-IR. The characteristic peaks selected for DPPTT and BAc are 1660 and 1723 cm^{-1}, respectively. IR-AFM overlay images (composition map) highlighting the distributions of DPPTT and BAc in BAc/DPPTT-x:y crosslinked films.

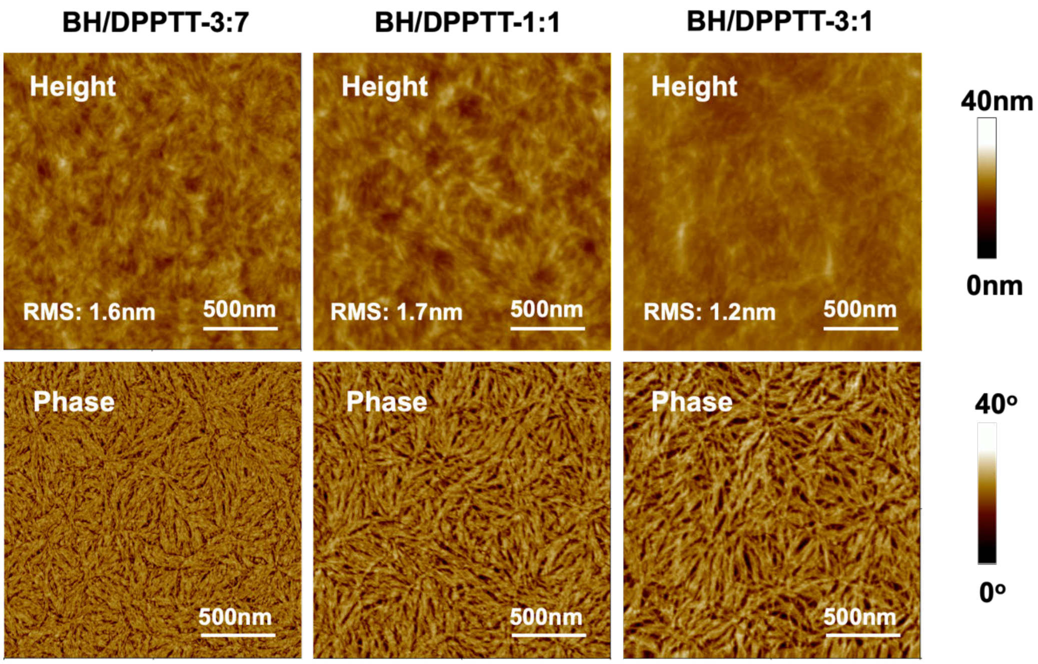

Supplementary Figure 11. AFM height and phase images of BH/DPPTT-x:y crosslinked films, where x:y is the BH-to-DPPTT weight ratio. The surface roughness is obtained from AFM height image.

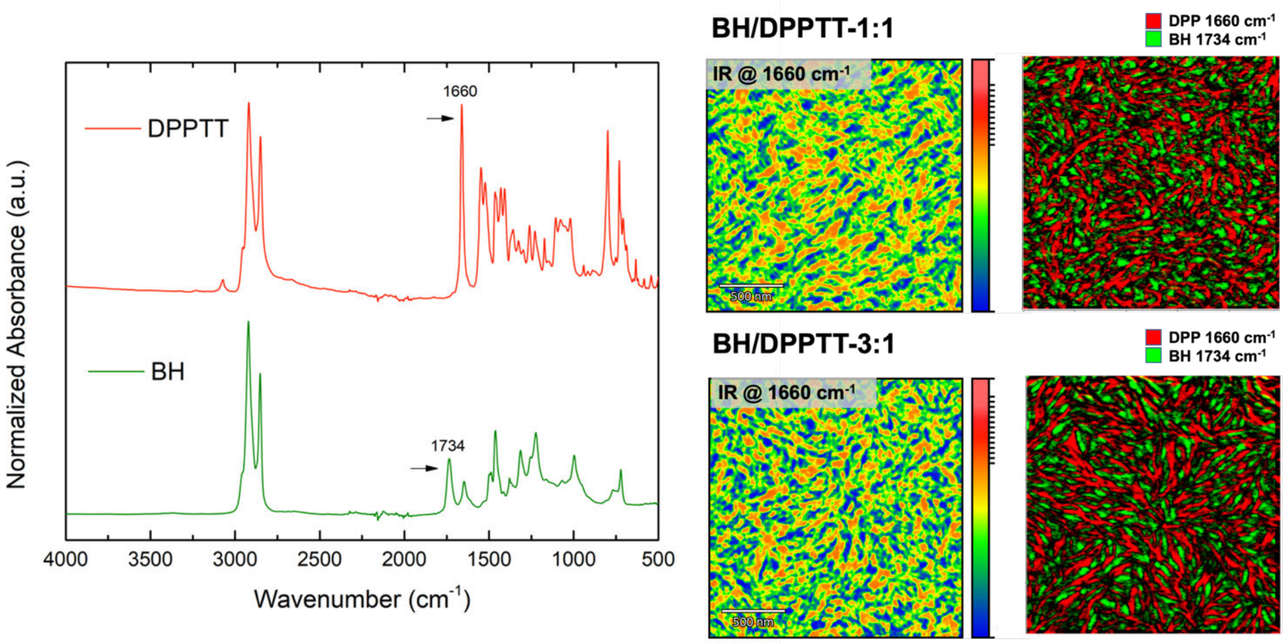

Supplementary Figure 12. IR absorption spectrum of DPPTT and crosslinked BH taken by FT-IR. The characteristic peaks selected for DPPTT and BH are 1660 and 1734 cm^{-1}, respectively. IR-AFM overlay images (composition map) highlighting the distributions of DPPTT and BH in BH/DPPTT-x:y crosslinked films.

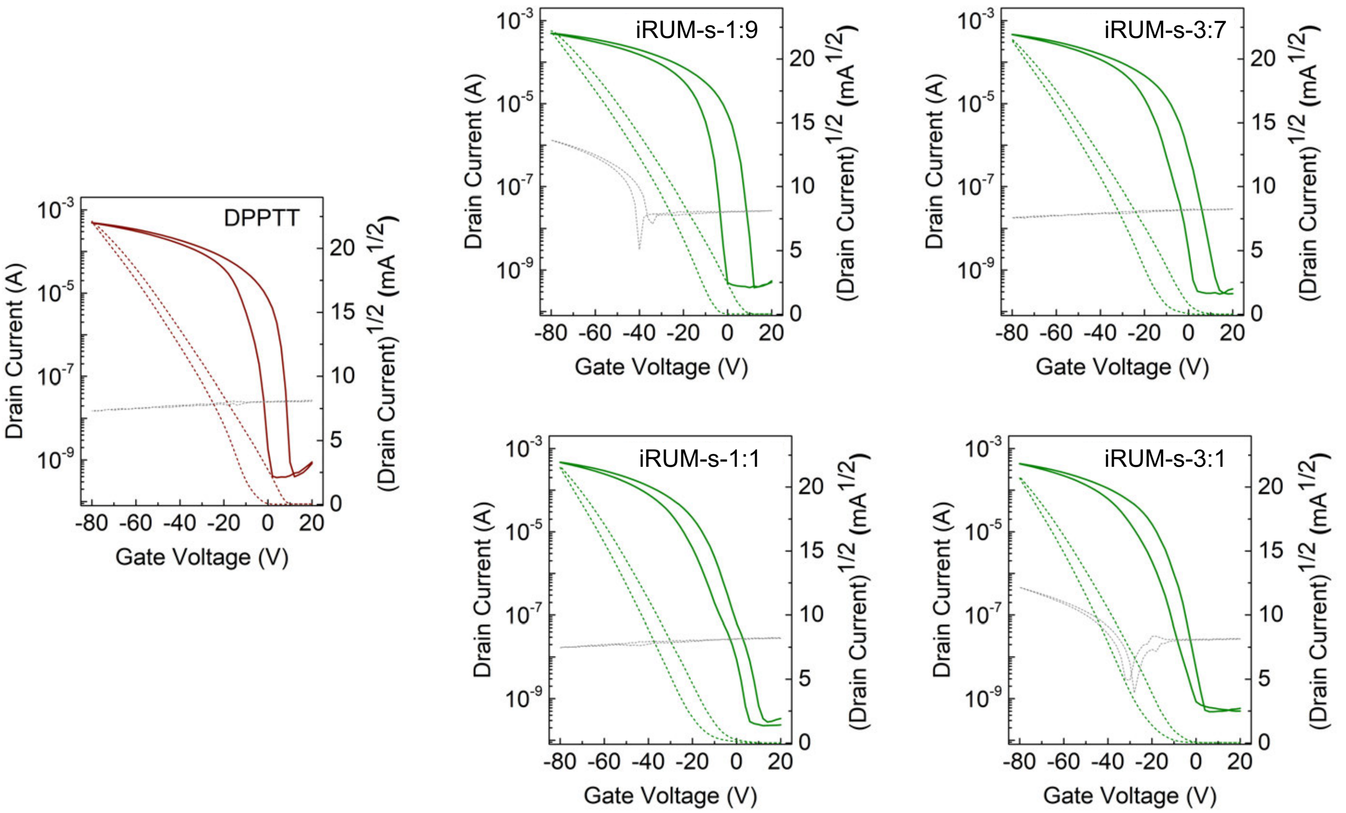

Supplementary Figure 13. Transfer curves from the bottom-gate top-contact transistors, with DPPTT or iRUM-s-x:y as semiconductor, highly-doped Si as gate electrode, MoO_3/Au as source/drain electrodes (W= 1000 µm, L= 50 µm) and octadecyltrimethoxysilane (OTS)-modified SiO_2 (300 nm) as dielectrics (solid lines: drain current; green dash lines: square root of the drain current; gray dash lines: gate current). The extracted mobility is well maintained at different BA proportion compared to neat DPPTT.

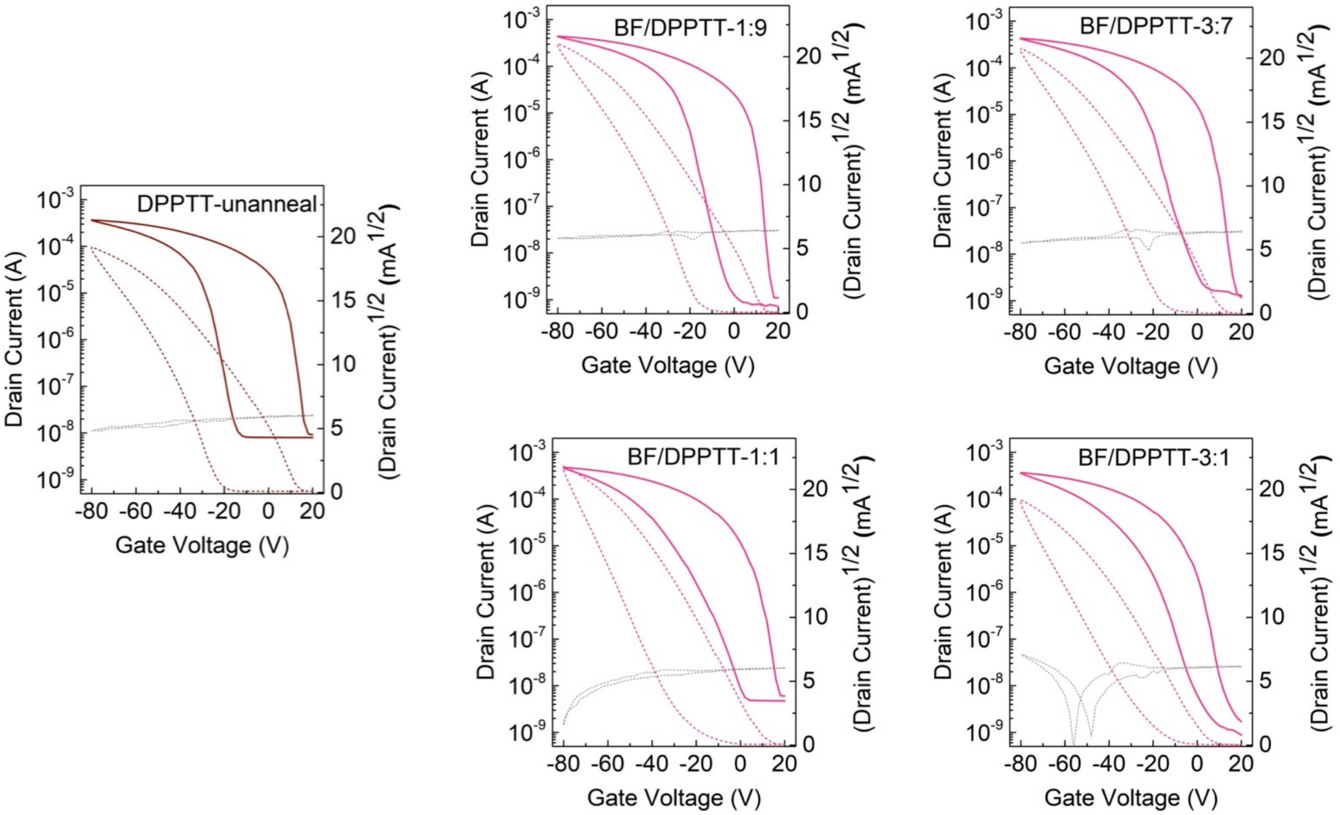

Supplementary Figure 14. Transfer curves from the bottom-gate top-contact transistors, with unannealed DPPTT or BF/DPPTT blend film as semiconductor, highly-doped Si as gate electrode, MoO_3/Au as source/drain electrodes (W= 1000 μm, L= 50 μm) and octadecyltrimethoxysilane (OTS)-modified SiO_2 (300nm) as dielectrics (solid lines: drain current; pink dash lines: square root of the drain current; gray dash lines: gate current). The extracted mobility is well maintained at different BF proportion compared to neat DPPTT.

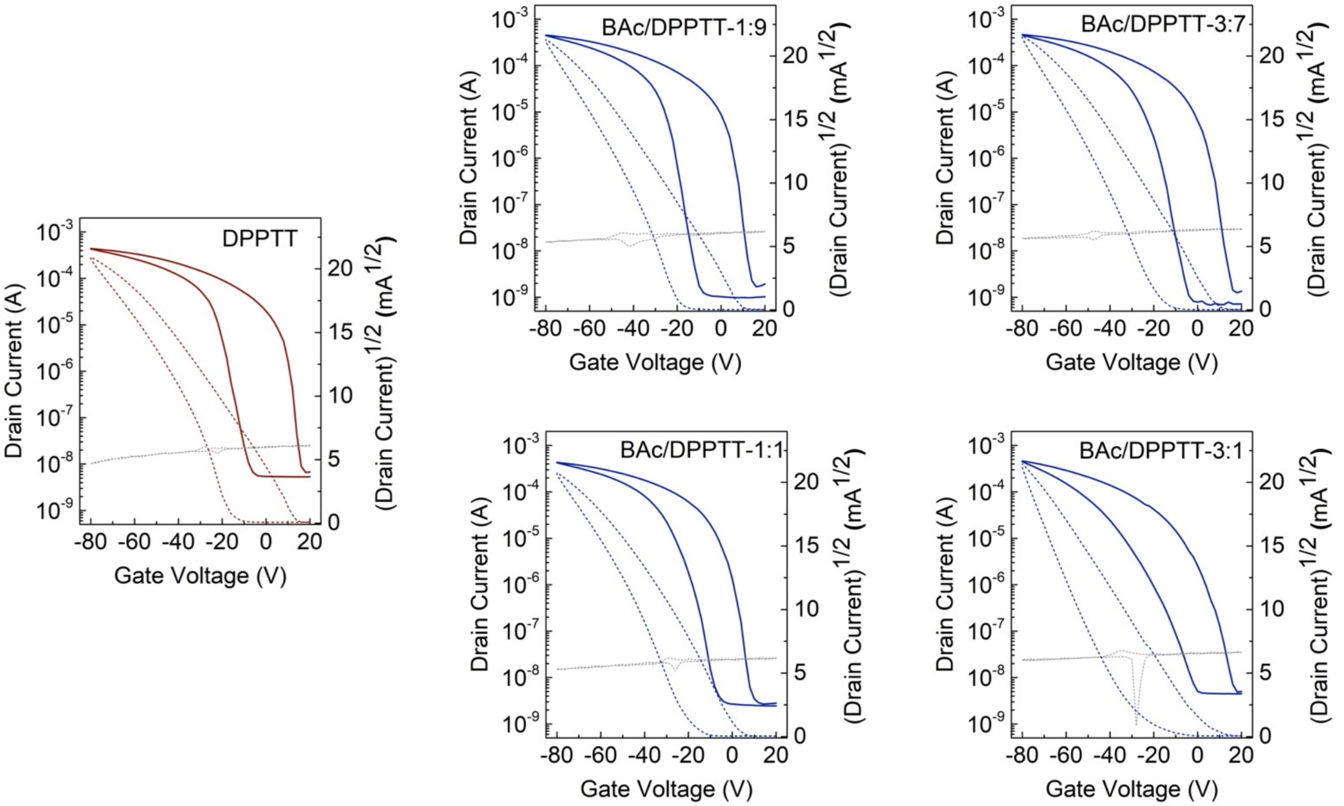

Supplementary Figure 15. Transfer curves from the bottom-gate top-contact transistors, with DPPTT or BAc/DPPTT crosslinked film as semiconductor, highly-doped Si as gate electrode, MoO_3/Au as source/drain electrodes (W= 1000 μm, L= 50 μm) and octadecyltrimethoxysilane (OTS)-modified SiO_2 (300nm) as dielectrics (solid lines: drain current; blue dash lines: square root of the drain current; gray dash lines: gate current). The extracted mobility is well maintained at different BAc proportion compared to neat DPPTT.

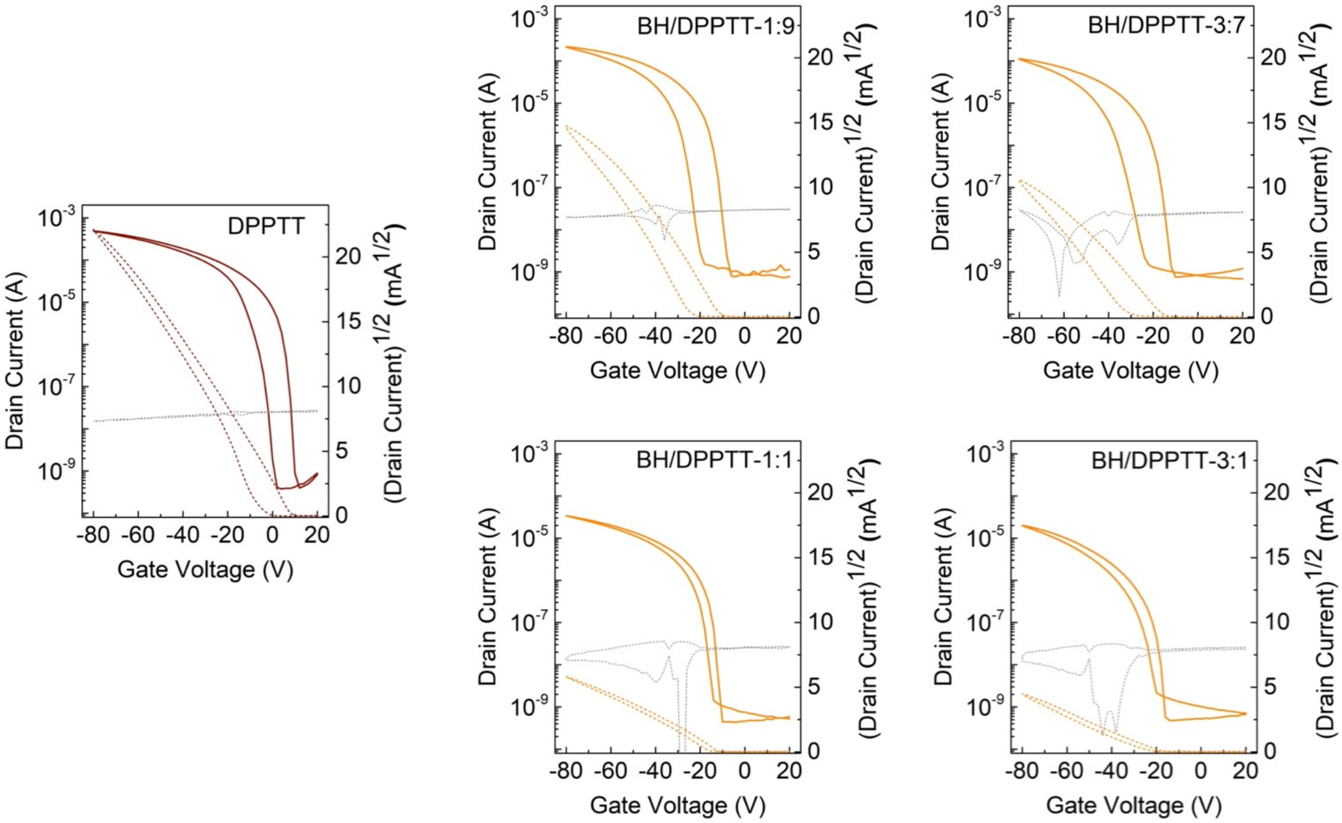

Supplementary Figure 16. Transfer curves from the bottom-gate top-contact transistors, with DPPTT or BH/DPPTT crosslinked film as semiconductor, highly-doped Si as gate electrode, MoO_3/Au as source/drain electrodes (W= 1000 μm, L= 50 μm) and octadecyltrimethoxysilane (OTS)-modified SiO_2 (300nm) as dielectrics (solid lines: drain current; orange dash lines: square root of the drain current; gray dash lines: gate current). The extracted mobility suffers from a drastic decay with BH proportion increasing compared to neat DPPTT.

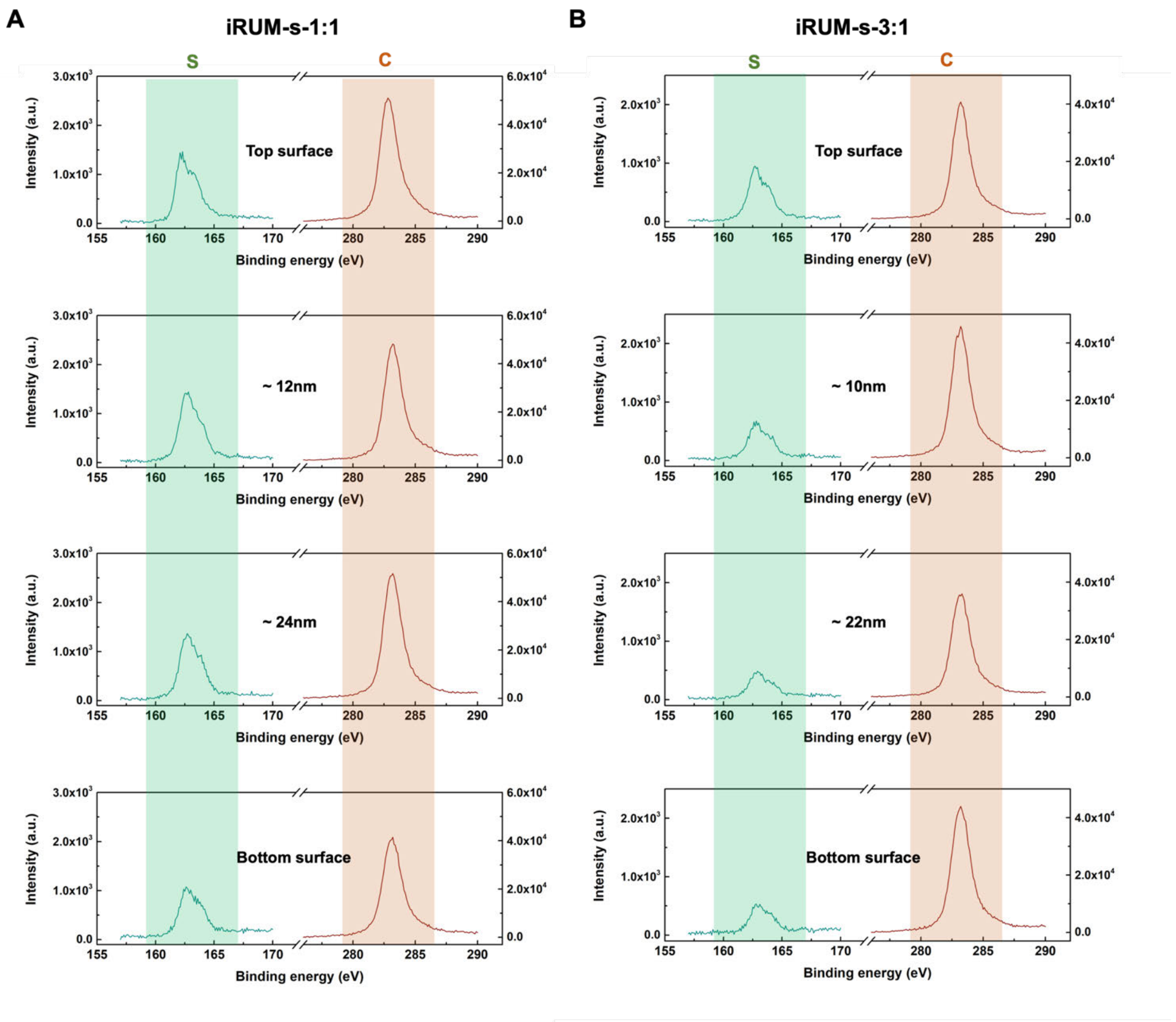

Supplementary Figure 17. XPS spectra obtained at four different depths of (**A**) iRUM-s-1:1 (thickness: 35nm) and (**B**) iRUM-s-3:1 (thickness: 32 nm) films, showing the change of S 2p peak and C 1s peak.

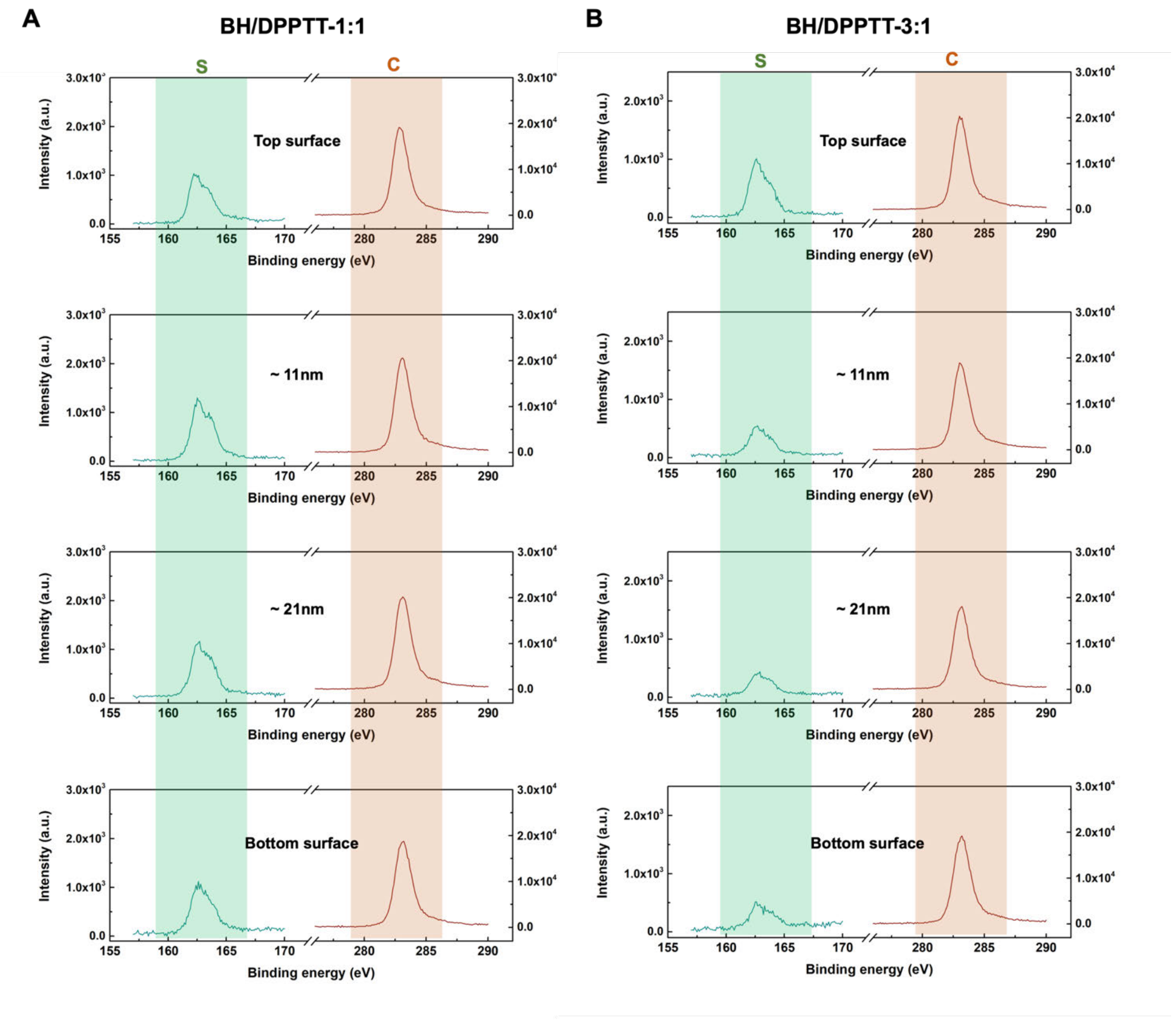

Supplementary Figure 18. XPS spectra obtained at four different depths of (**A**) BH/DPPTT-1:1 (thickness: 35nm) and (**B**) BH/DPPTT-3:1 (thickness: 32 nm) films, showing the change of S 2p peak and C 1s peak.

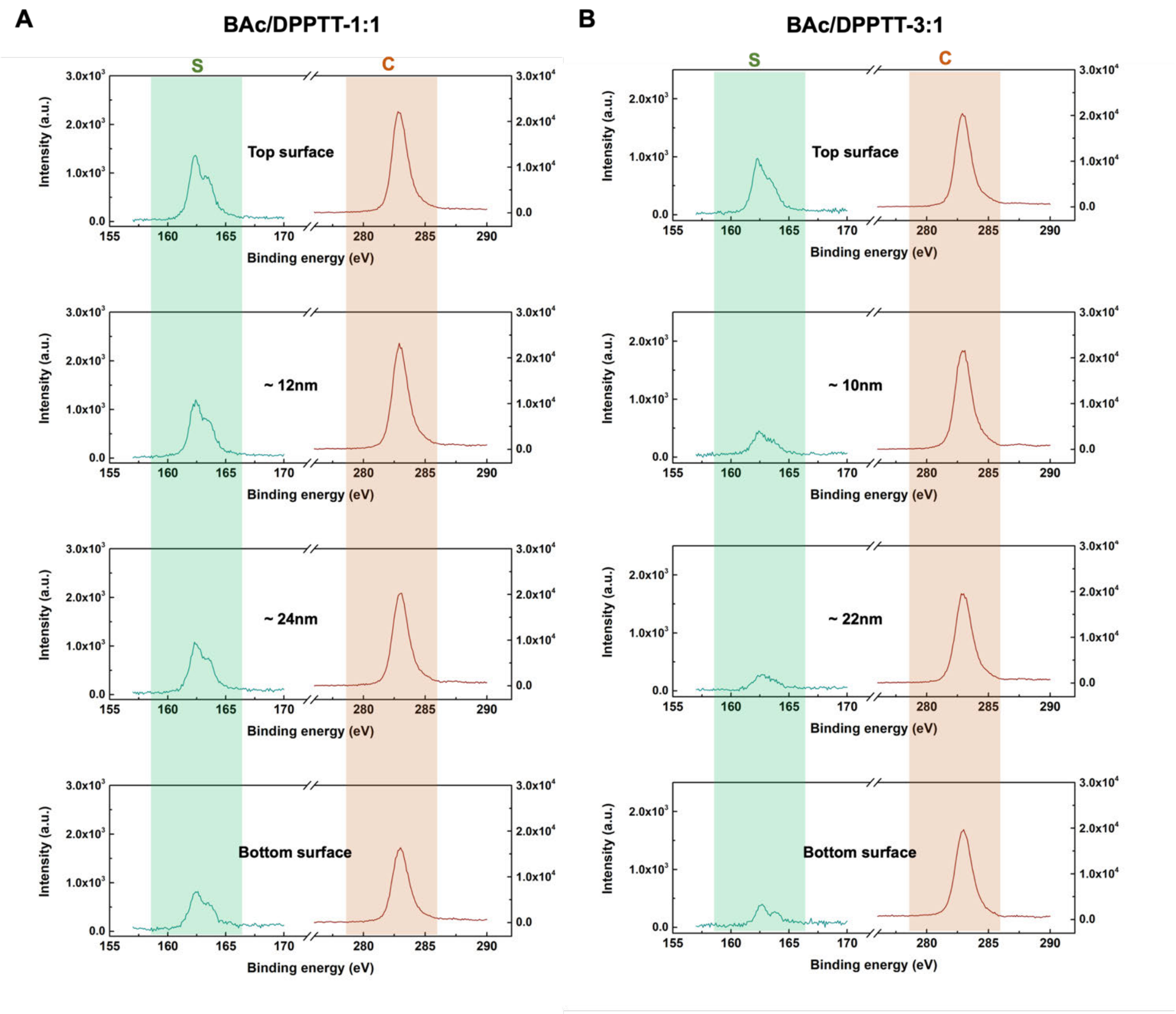

Supplementary Figure 19. XPS spectra obtained at four different depths of (**A**) BAc/DPPTT-1:1 (thickness: 35nm) and (**B**) BAc/DPPTT-3:1 (thickness: 32 nm) films, showing the change of S 2p peak and C 1s peak.

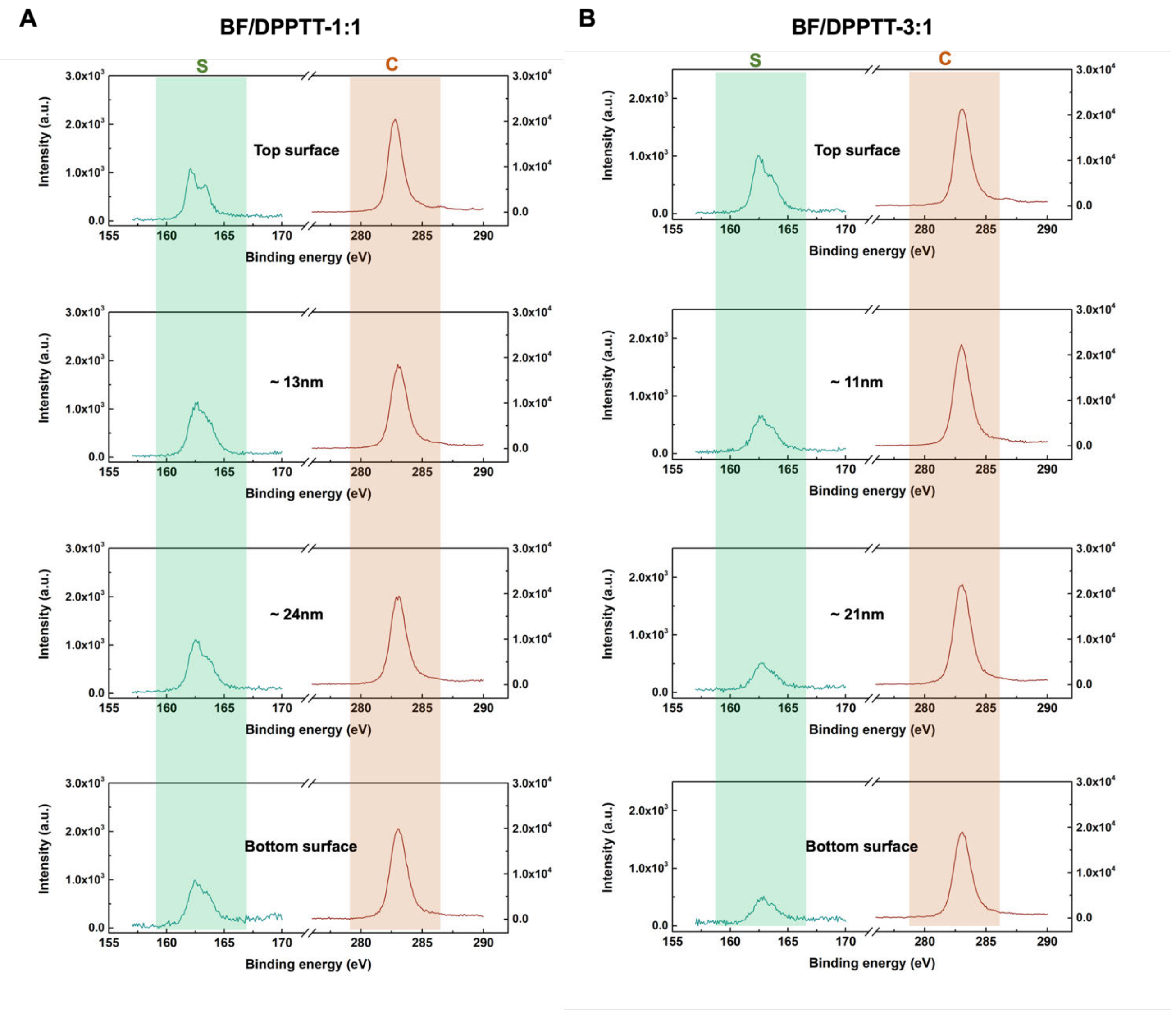

Supplementary Figure 20. XPS spectra obtained at four different depths of (**A**) BF/DPPTT-1:1 (thickness: 35nm) and (**B**) BF/DPPTT-3:1 (thickness: 32 nm) films, showing the change of S 2p peak and C 1s peak.

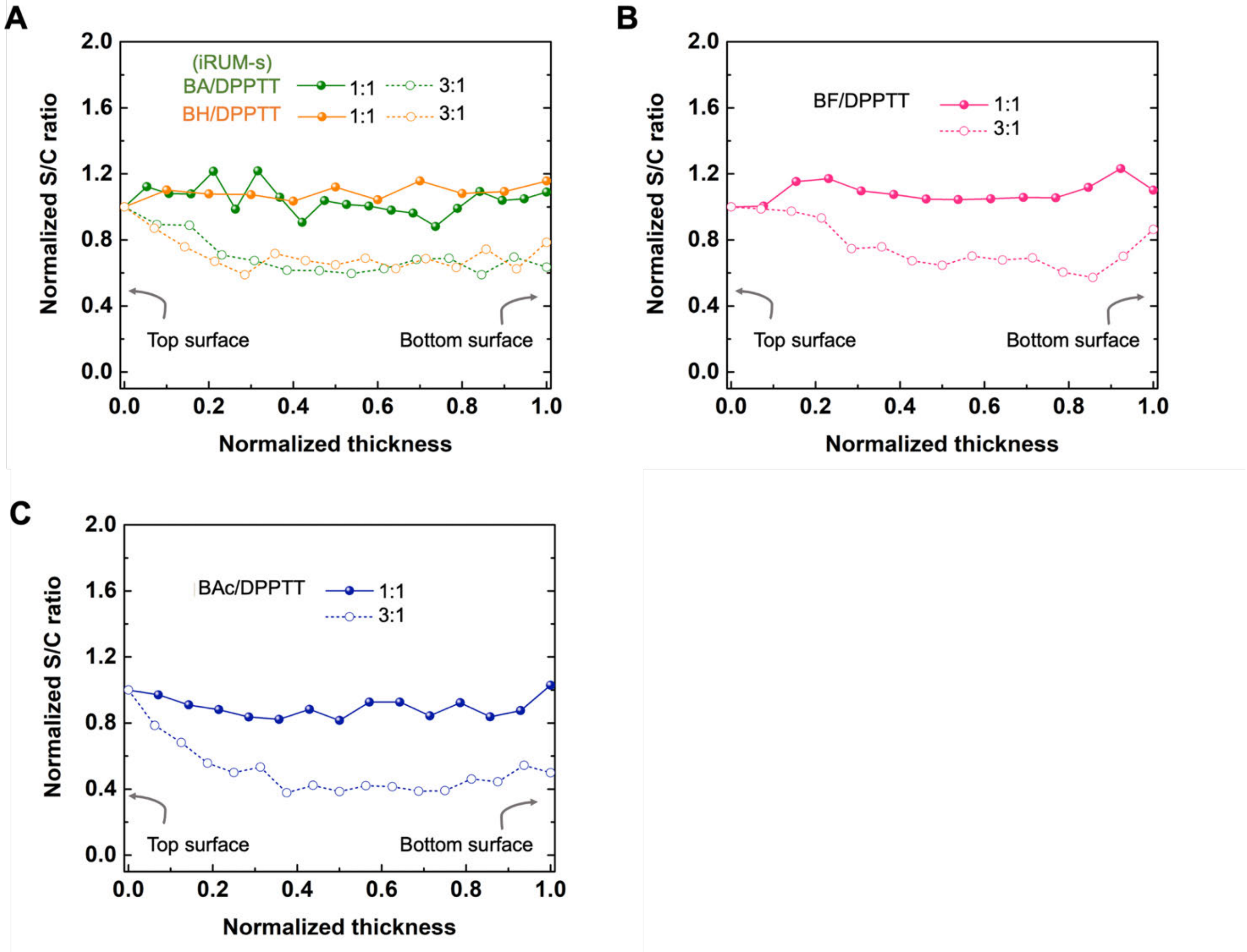

Supplementary Figure 21. The normalized ratios between S 2p peak and C 1s peak (normalized S/C ratio) from XPS spectra obtained at a series of different depths of (**A**) iRUM-s-x:y and BH/DPPTT-x:y crosslinked films, (**B**) BF/DPPTT blend films and (**C**) BAc/DPPTT crosslinked films. Since S only exists in DPPTT, the S/C ratio qualitatively reflects the distribution of DPPTT across film thickness (around 30-35 nm).

Note: When polybutadiene-based precursor/DPPTT is 1:1 in w.t.%, the DPPTT exhibit similarly uniform distribution across film thickness in all of the crosslinked or blend films. When polybutadiene-based precursor/DPPTT is 3:1 in w.t.%, small degree of vertical phase separation occurs. BAc/DPPTT-3:1 crosslinked film has larger phase separation, while iRUM-s-3:1, BH/DPPTT-3:1 crosslinked film and BF/DPPTT-3:1 blend film exhibited similar and more uniform DPPTT distribution. Therefore, the difference in vertical distribution of DPPTT that is not observed here cannot fully explain the electrical performance maintenance of iRUM-s and dramatic degradation of BH/DPPTT crosslinked films.

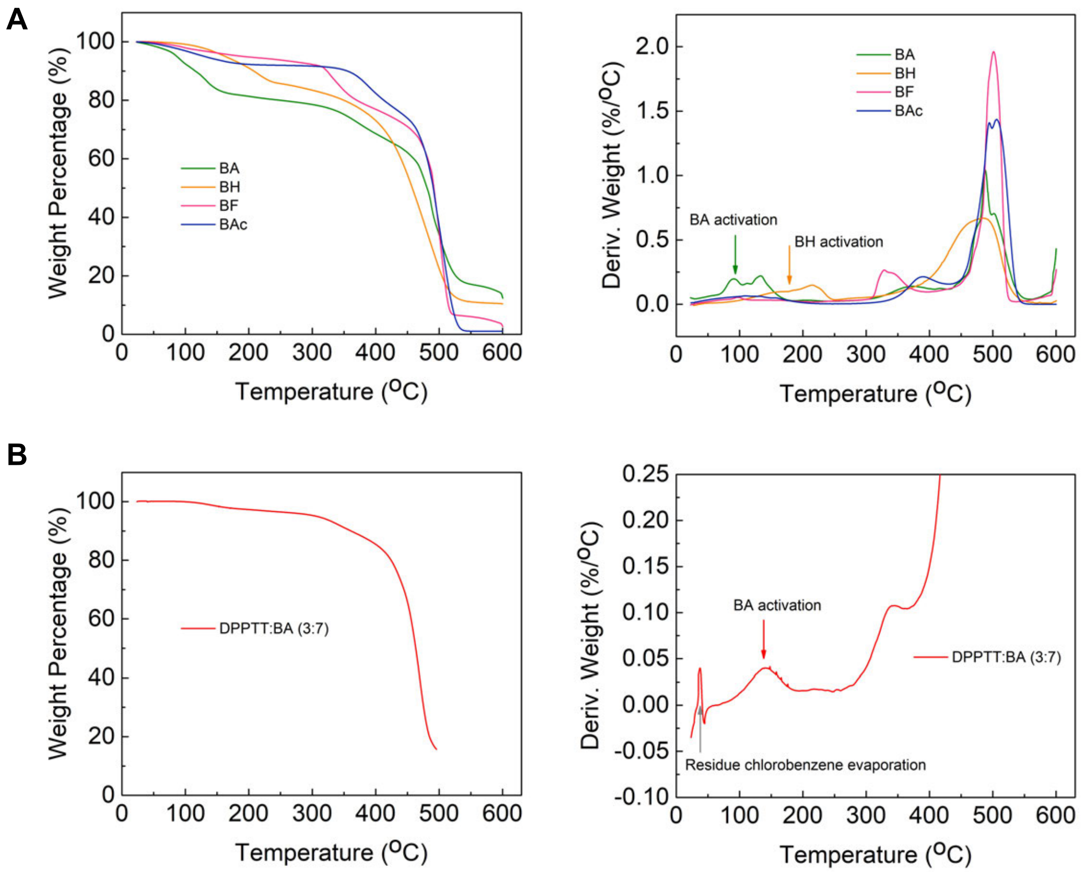

Supplementary Figure 22. Thermogravimetric analysis (TGA) of (A) different polybutadiene-derived precursors (B) **DPPTT**/**BA**-3:7 blend. Left: weight loss at different temperatures. Right: Derivative of weight% over temperature as a function of temperature. The azide started to react at a lower temperature for **BA** than **BH**. This confirms the reactivity of azide/C=C cycloaddition is higher than that of azide/C-H insertion reported previously[9,10]. **BA** activation temperature in **DPPTT**/**BA** blend is in the similar temperature range as that of pure **BA**.

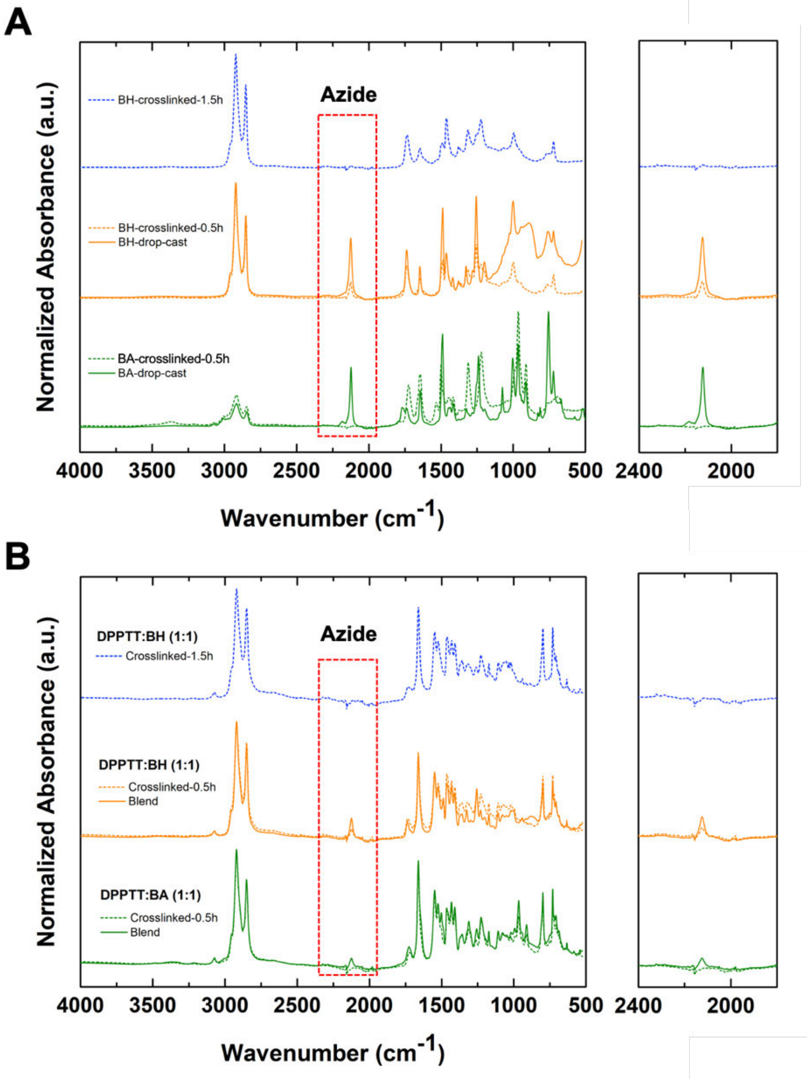

Supplementary Figure 23. FTIR spectrum of (**A**) Neat **BA** and **BH** before and after thermal crosslinking at 150 °C; (**B**) **BA/DPPTT**-1:1 and **BH/DPPTT**-1:1 blend after thermal crosslinking.

Note: The different reactivity of **BA** and **BH** can be seen by the time needed for the azide characteristic peak to disappear in FTIR. The drop-casted **BA** and **BH** both have sharp azide stretch peak at 2120 cm^{-1}. After thermal crosslinking at 150 °C, the azide peak of BA completely disappeared. On the other hand, the azide peak of BH still remained, and it disappeared after thermal crosslinking for another one hour, which indicates that BH needs higher energy to be fully activated, thus a lower crosslinking efficiency. The same trend can be observed in BA/DPPTT and BH/DPPTT blends. Referring to this, we decided the thermal annealing condition for iRUM-s film and BH/DPPTT crosslinked film preparation.

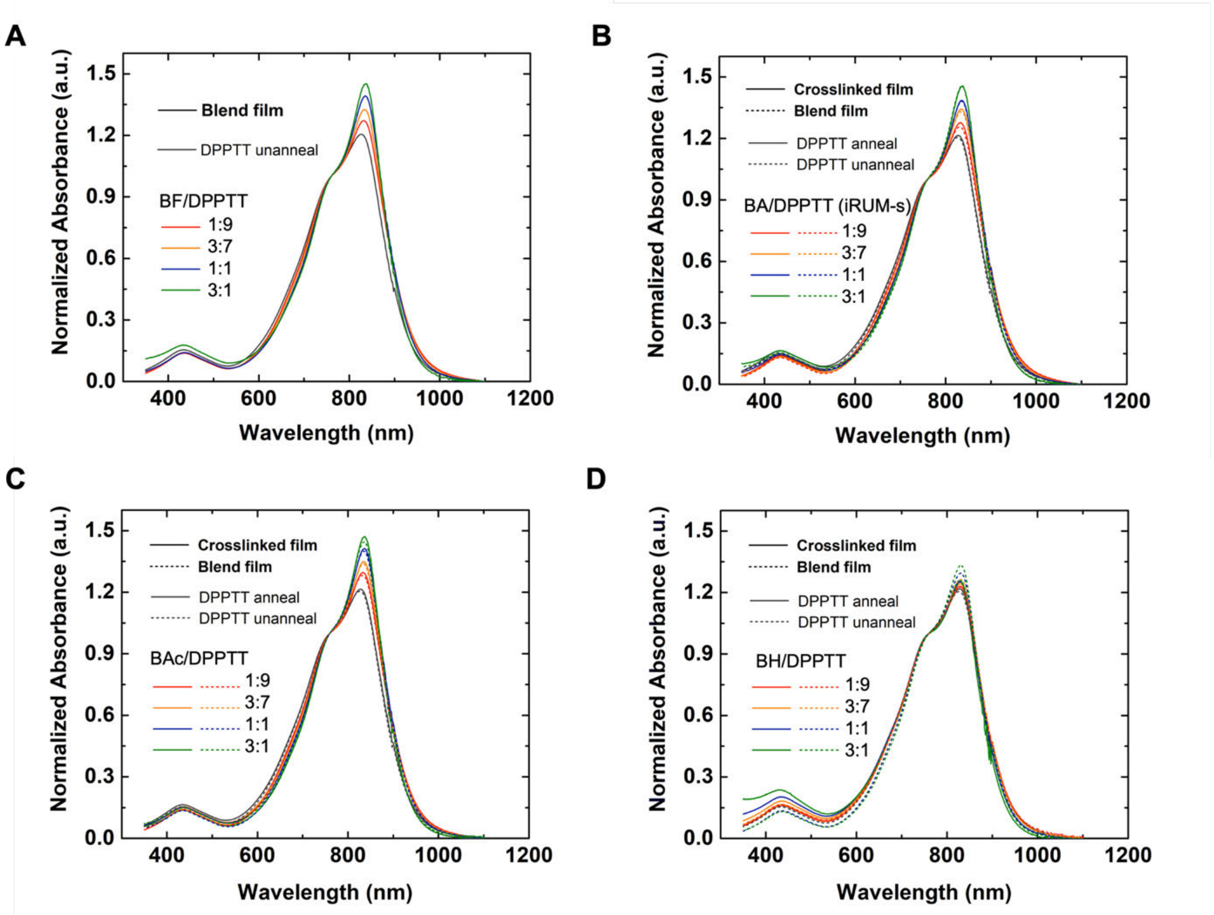

Supplementary Figure 24. Normalized UV-Vis absorption spectrum of (**A**) BF/DPPTT-x:y blend films (**B**) BA/DPPTT-x:y blend and crosslinked films (**C**) BAc/DPPTT-x:y blend and crosslinked films (**D**) BH/DPPTT-x:y blend and crosslinked films, where x:y is polybutadiene-based precursor to DPPTT in weight ratio.

Note: a $\pi - \pi^*$ transition was recorded around 450nm for all the films. Their internal charge transfer peaks between 700 and 900 nm showed two distinct vibronic bands: the 0−1 and 0−0 transitions, with the lower energy 0−0 peak (higher wavenumber) typically attributed to DPPTT aggregation. When normalized to the 0−1 peak, an increase in the intensity of 0-0 peak and a slight red-shift are indicative of a higher degree of DPPTT aggregation. For BF/DPPTT-x:y blend films, with BF proportion increasing, DPPTT aggregation increases. This is due to the introduction of a secondary component into DPPTT film, resulting in higher interaction between DPPTT polymer chains, which is also observed in previously reported conjugated polymer/elastomer blending systems (ref). For BA/DPPTT-x:y, BAc/DPPTT-x:y and BH/DPPTT-x:y blend films, such an increase in DPPTT aggregation can also be observed. However, after crosslinking, DPPTT aggregation changed in different manners. BAc/DPPTT-x:y crosslinked films exhibited higher DPPTT aggregation than blend films, which is due to the formation of a rubber matrix that contributes to stronger DPPTT chain interactions than that from precursors. For BA/DPPTT-x:y (iRUM-s-x:y) crosslinked films, a slight increase in DPPTT aggregation can also be observed than blend films. However, the degree of DPPTT aggregation increase is lower than that observed in BAc/DPPTT-x:y crosslinked films. This can be explained by the reactivity difference between azide/double-bond cycloaddition and azide/C-H insertion. A higher proportion of BA will react with the double bonds on itself to create a rubber matrix, contributing to increase in DPPTT aggregation. However, a small proportion of BA can also react with DPPTT side chains, thus disrupting DPPTT aggregation. This claim can be supported by the fact that BH/DPPTT-x:y crosslinked films exhibited obvious lower DPPTT aggregation than blend films. Without such reactivity difference, the DPPTT aggregation was continuously disrupted with BH proportion increasing.

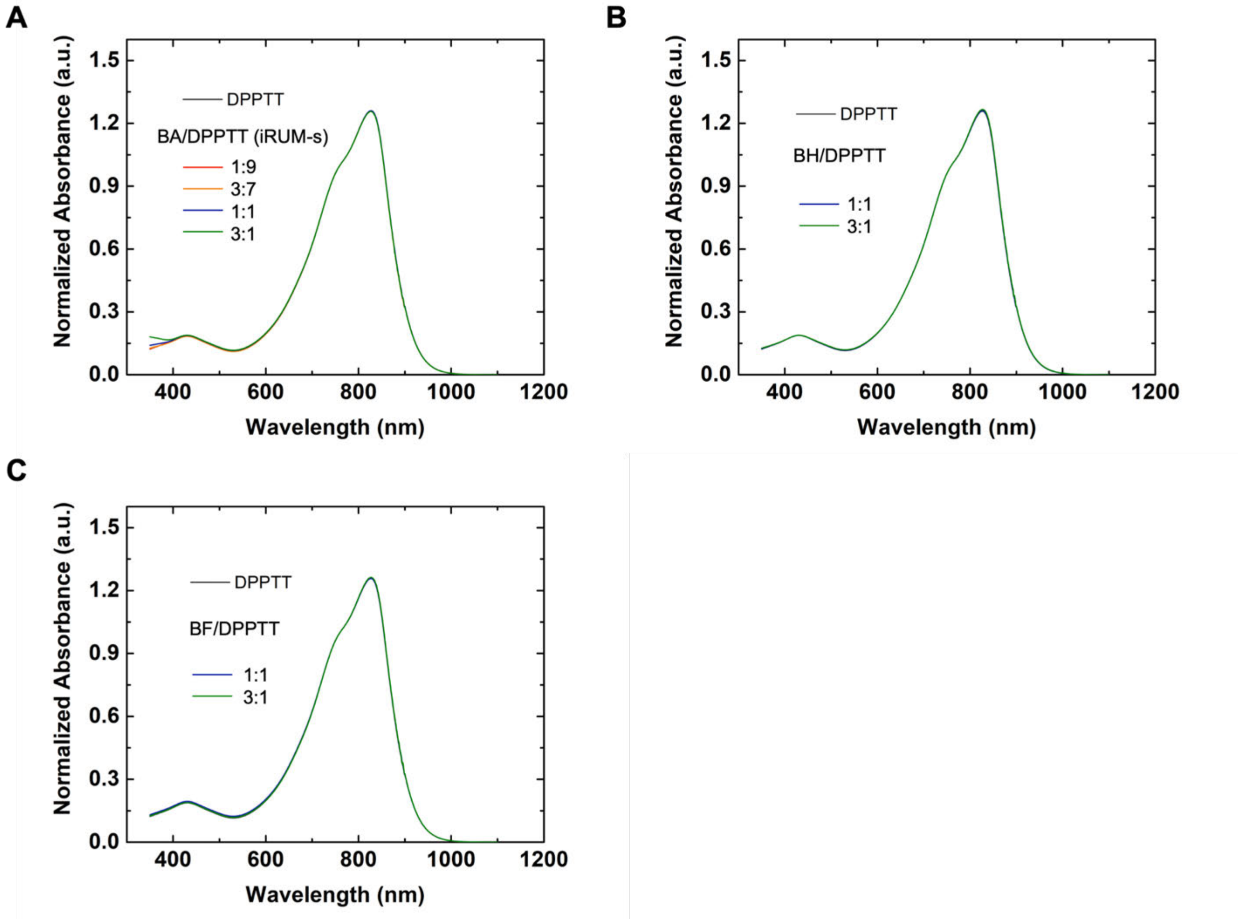

Supplementary Figure 25. Normalized UV-Vis absorption spectrum of the mixture of (**A**) BA/DPPTT-x:y (**B**) BH/DPPTT-x:y (**C**) BF/DPPTT-x:y in solution, where x:y is polybutadiene-based precursor to DPPTT in weight ratio, and concentration is 0.01mg/mL in chlorobenzene.

Note: Different from the trend observed in thin-film UV-Vis absorption, the mixture of polybutadiene-based precursor and DPPTT didn't exhibit any change in aggregation compared with neat DPPTT solution. This indicates the excellent miscibility of different polybutadiene-derived precursors with DPPTT, resulting from the highly flexible, random coil-like precursor backbones.

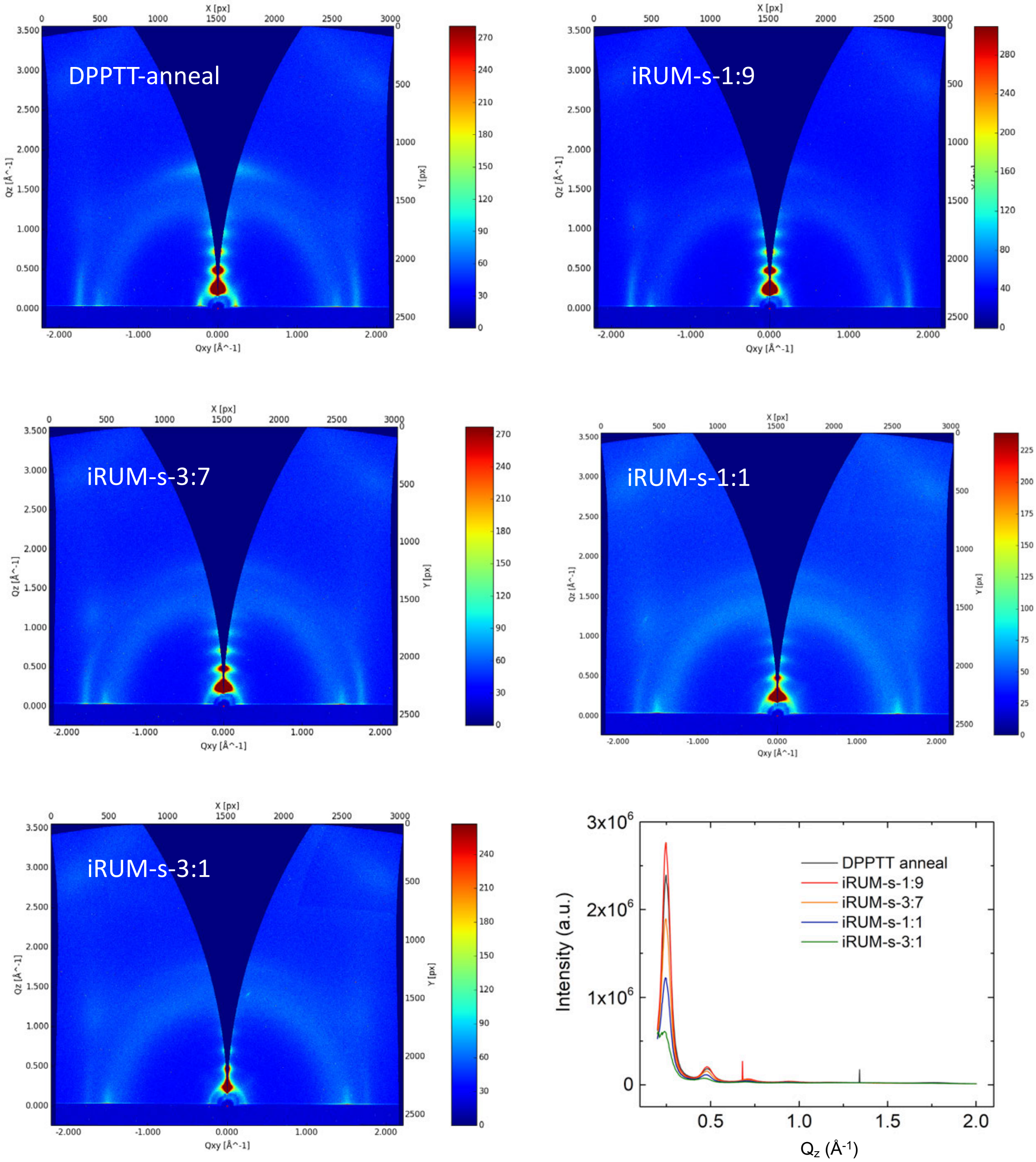

Supplementary Figure 26. 2D Grazing-incidence X-ray diffraction (GIXD) patterns and polarization-corrected meridian line cuts along the Q_z axis of DPPTT and iRUM-s-x:y films. The plots were scaled by illuminated volume and exposure time.

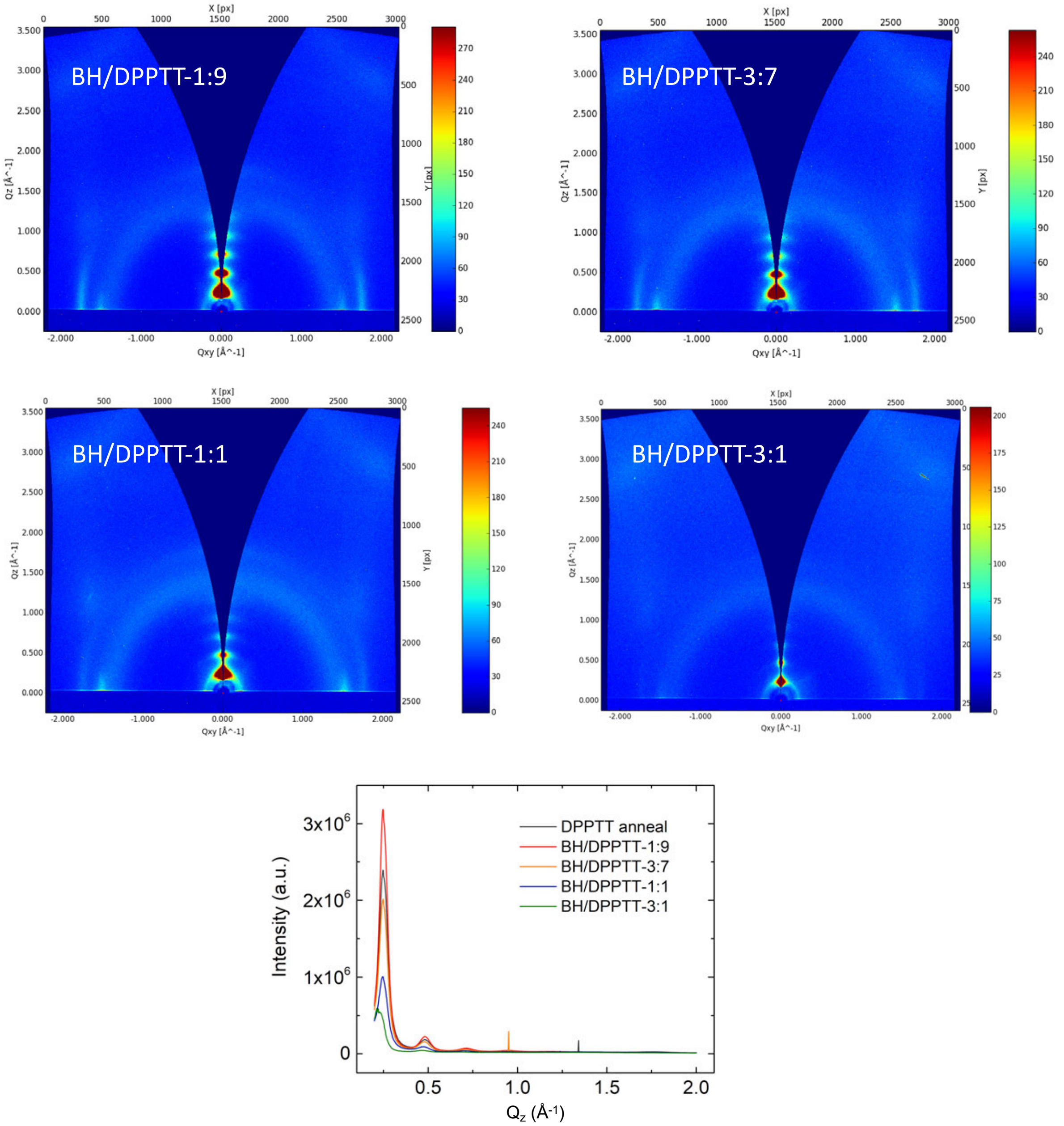

Supplementary Figure 27. 2D Grazing-incidence X-ray diffraction (GIXD) patterns and polarization-corrected meridian line cuts along the Q_z axis of BH/DPPTT-x:y crosslinked films.

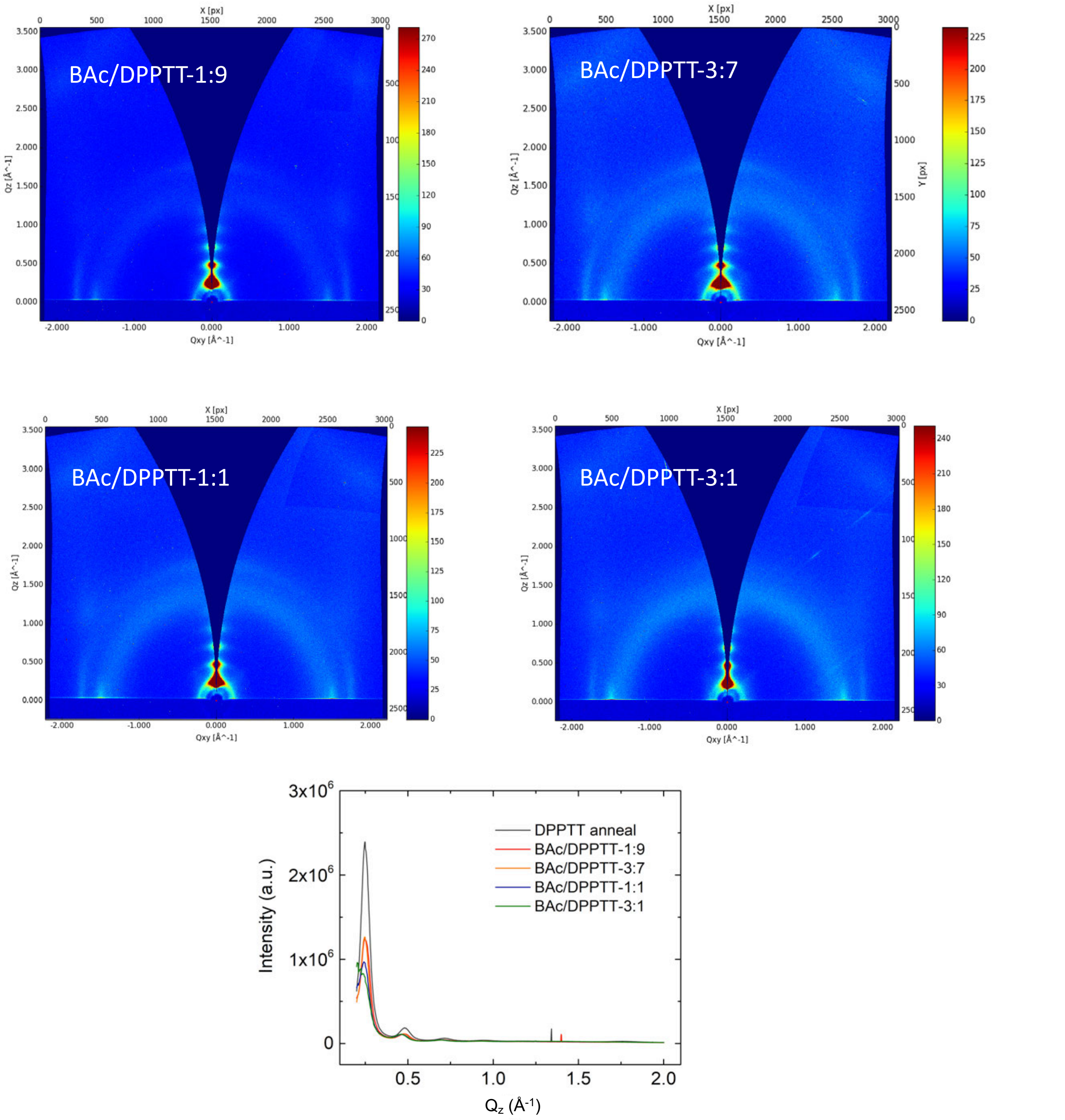

Supplementary Figure 28. 2D Grazing-incidence X-ray diffraction (GIXD) patterns and polarization-corrected meridian line cuts along the Q_z axis of BAc/DPPTT-x:y crosslinked films.

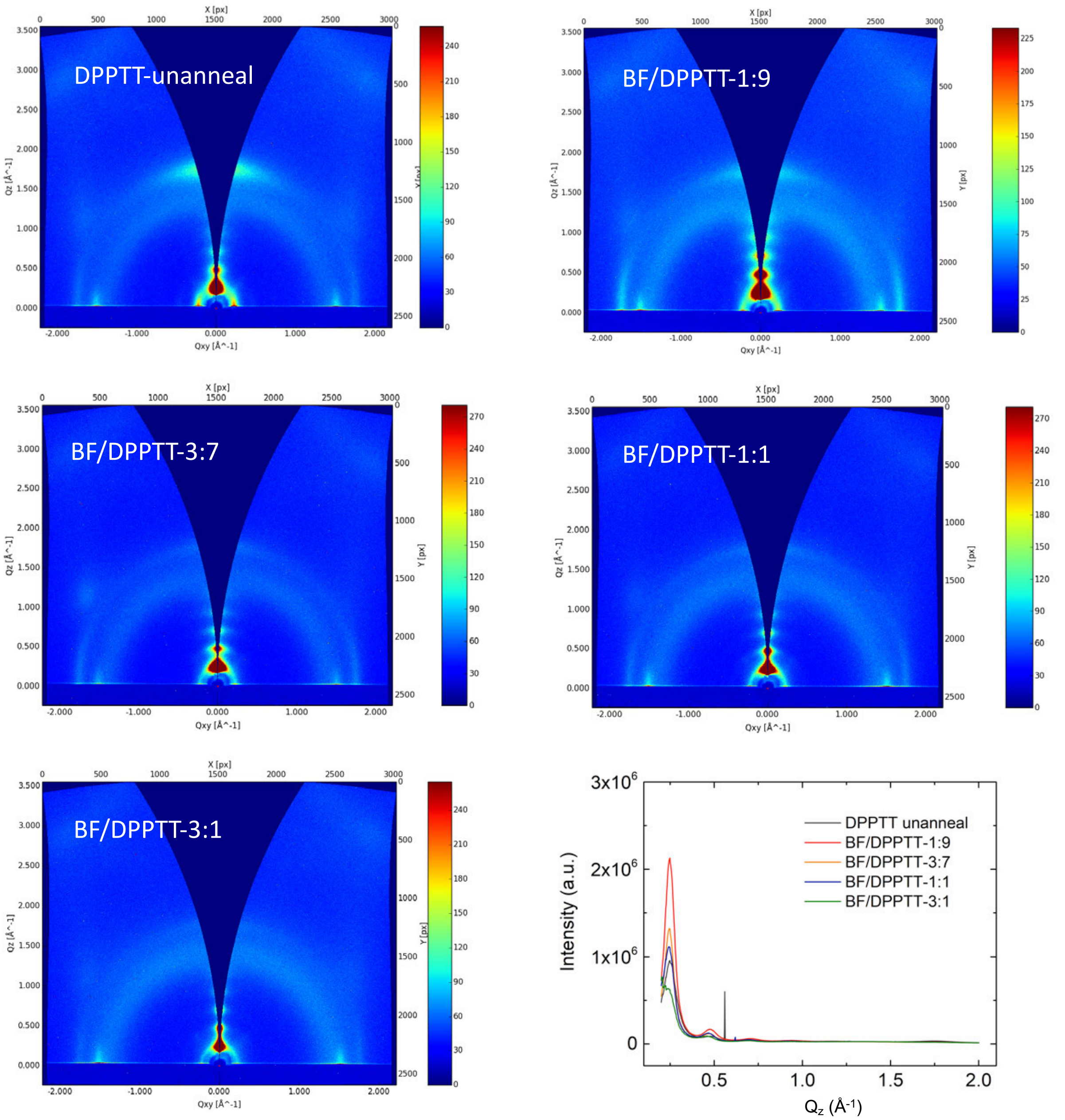

Supplementary Figure 29. 2D Grazing-incidence X-ray diffraction (GIXD) patterns and polarization-corrected meridian line cuts along the Q_z axis of BF/DPPTT-x:y blend films.

Supplementary Table 2. Crystallographic parameters of iRUM-s-x:y films.

BA/DPPTT	Lamella spacing (Å)[a]	Lamella FWHM ($Å^{-1}$)[b]	Coherence length (nm)[c]	π-π spacing (Å)[d]	π-π FWHM ($Å^{-1}$)
DPPTT-anneal	26.01	0.0651	9.7	3.57	0.11
1:9	26.11	0.0636	9.9	3.56	0.11
3:7	26.27	0.0633	9.9	3.56	0.11
1:1	26.43	0.0625	10.1	3.56	0.10
3:1	26.89	0.0635	9.9	3.55	0.11

Supplementary Table 3. Crystallographic parameters of BH/DPPTT-x:y crosslinked films.

BH/DPPTT	Lamella spacing (Å)[a]	Lamella FWHM ($Å^{-1}$)[b]	Coherence length (nm)[c]	π-π spacing (Å)[d]	π-π FWHM ($Å^{-1}$)
1:9	26.04	0.0645	9.7	3.57	0.11
3:7	26.23	0.0653	9.6	3.57	0.11
1:1	26.42	0.0651	9.7	3.55	0.10
3:1	26.83	0.0683	9.2	3.60	0.10

Supplementary Table 4. Crystallographic parameters of BAc/DPPTT-x:y crosslinked films.

BAc/DPPTT	Lamella spacing (Å)[a]	Lamella FWHM ($Å^{-1}$)[b]	Coherence length (nm)[c]	π-π spacing (Å)[d]	π-π FWHM ($Å^{-1}$)
1:9	26.14	0.0637	9.9	3.58	0.11
3:7	26.42	0.0629	10.0	3.57	0.11
1:1	26.88	0.0620	10.1	3.57	0.11
3:1	27.19	0.0638	9.8	3.57	0.10

Supplementary Table 5. Crystallographic parameters of BF/DPPTT-x:y blend films.

BF/DPPTT	Lamella spacing (Å)[a]	Lamella FWHM ($Å^{-1}$)[b]	Coherence length (nm)[c]	π-π spacing (Å)[d]	π-π FWHM ($Å^{-1}$)
DPPTT-unanneal	25.94	0.0724	8.7	3.57	0.12
1:9	26.25	0.0714	8.8	3.58	0.11
3:7	26.57	0.0670	9.4	3.57	0.11
1:1	26.81	0.0673	9.3	3.56	0.11
3:1	27.13	0.0672	9.3	3.56	0.10

[a] Extracted from fitting (200) diffraction peak, the stacking distance was calculated by Bragg equation ($d = 2\pi/q$, q represents peak center position. [b] FWHM represents full width at half maximum for the peak. [c] Crystallite size is calculated from (200) diffraction peak using Bragg equation [d] Extracted from fitting (010) diffraction peak.

Supplementary Note 2. GIXD analysis (Supplementary Figure 25-28, Supplementary Table 2-5).

GIXD provides information regarding the crystalline region of conjugated polymer thin film. As shown from 2D diffraction patterns, all of the blend/crosslinked films exhibit an edge-on orientation, with out-of-plane (h00) lamella stacking peaks and in-plane (010) π-π stacking peak. Compared with neat DPPTT film (with or without thermal annealing), there is negligible change in π-π spacing (~3.6 Å) but an increase in lamella spacing, suggesting the more favorable interaction between polybutadiene-derived precursors with DPPTT alkyl side chains than conjugated backbones. However, this change in lamella spacing is small relative to the length of polybutadiene-derived precursors, thus, it is unlikely that the precursors inserted into the crystalline region. Instead, they are distributed mostly into the amorphous regions of conjugated polymers. For BF/DPPTT-x:y blend films, a decrease in FWHM of the (200) lamella stacking peak is observed with BF proportion increasing, which corresponds to an increase in coherence length. For the same value of x:y (precursor/DPPTT), the coherence length of BH/DPPTT-x:y crosslinked film is smaller than that of iRUM-s-x:y and BAc/DPPTT-x:y crosslinked films. This supports the claim that a higher proportion of azide/C-H insertion undergoes after blending DPPTT with BH than BA, thus chain aggregation and packing of polymer semiconductor is disrupted more in BH/DPPTT-x:y crosslinked films than iRUM-s-x:y films.

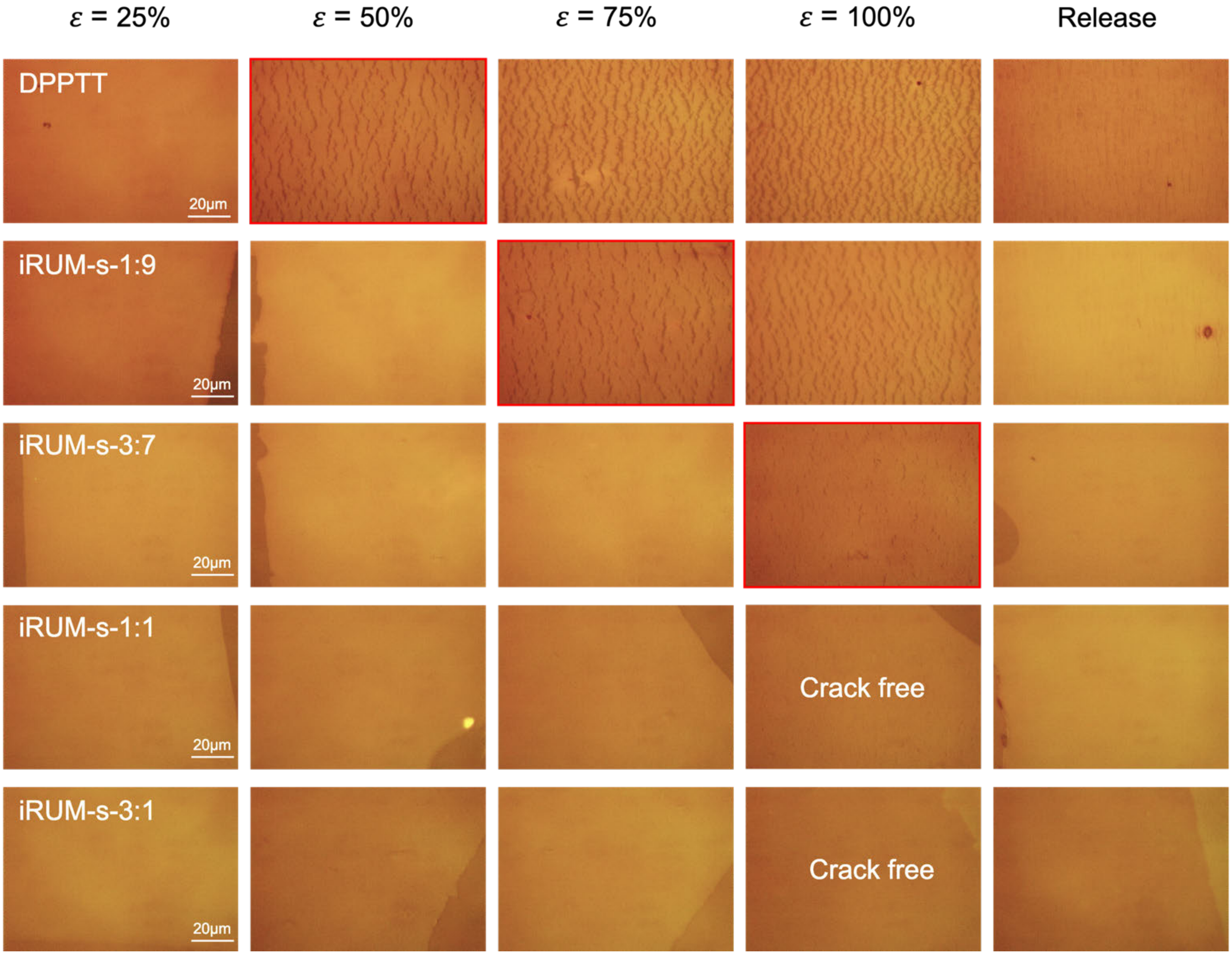

Supplementary Figure 30. Crack on-set strains of DPPTT and iRUM-s-x:y films, as measured by "film-on-elastomer" using optical microscope.

Note: "Film-on-elastomer": the semiconductor film is transferred onto a supported PDMS substrate (1 mm), and its crack formation is monitored under optical microscope with increasing strains. This measurement is relevant, as the semiconductor film is usually deformed on a supported dielectric in electronic devices. Our parameter of interest is crack on-set strain, which is usually used to evaluate the stretchability of a semiconductor film. As demonstrated in previous reports, the main failure mechanism for a semiconductor film during stretching is crack propagation, which can hinder charge transport and the device will suffer from dramatic electrical performance drop.

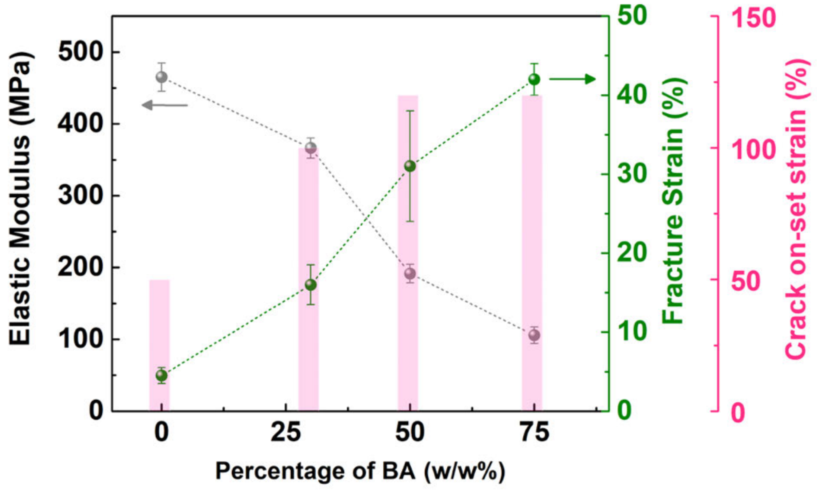

Supplementary Figure 31. Summarized mechanical properties characterization results for iRUM-s-x:y films. The elastic modulus and fracture strain are extracted from pseudo free-standing tensile tests (film-on-water). The crack on-set strain is obtained from "film-on-elastomer" measurement. The PDMS fractured when stretched to 120% strain, thus the crack on-set strain for iRUM-s-1:1 and iRUM-s-3:1 is defined as 120%.

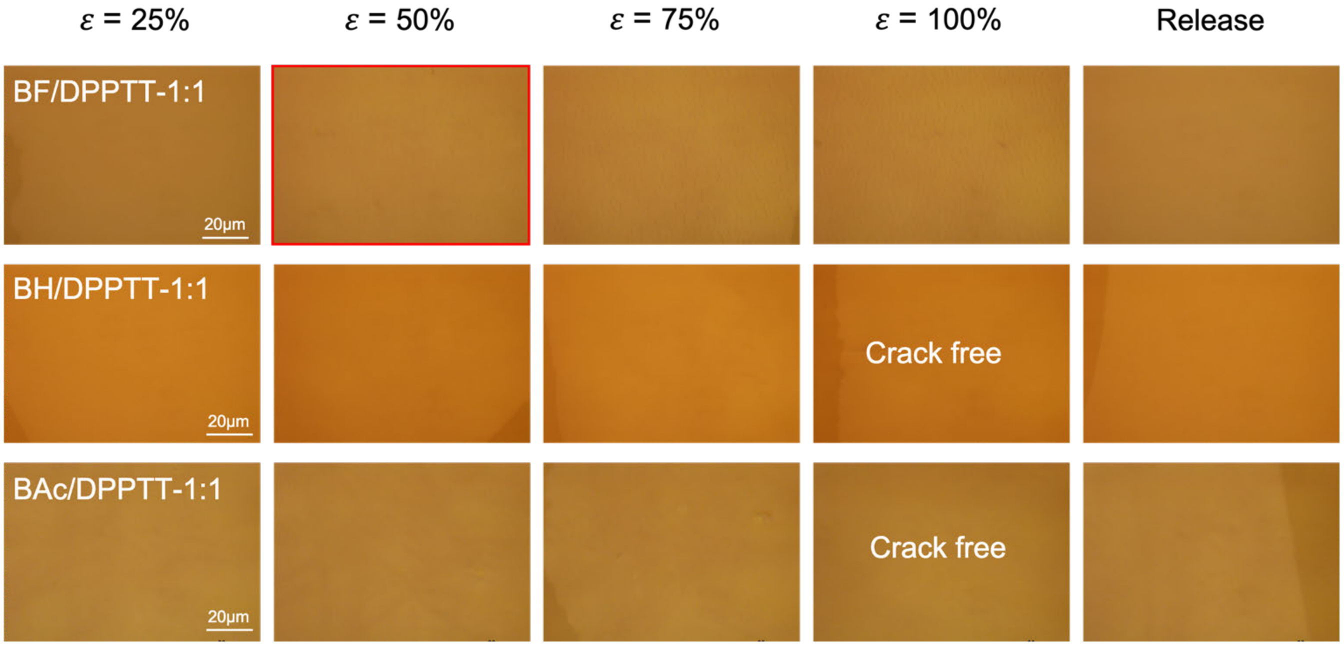

Supplementary Figure 32. Crack on-set strains of BF/DPPTT-1:1 blend film, BH/DPPTT-1:1 and BAc/DPPTT-1:1 crosslinked films, as measured by "film-on-elastomer" by optical microscope.

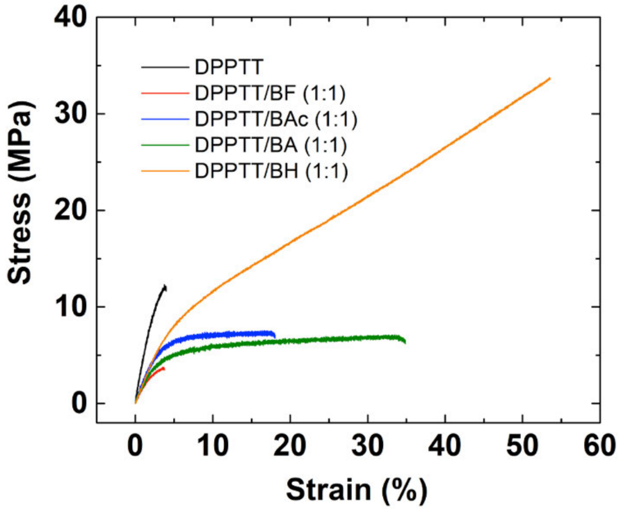

Supplementary Figure 33. The representative stress-strain curves for DPPTT, iRUM-s (BA/DPPTT) films, BF/DPPTT blend films, BAc/DPPTT and BH/DPPTT crosslinked films, where the precursor-to-DPPTT weight ratio is 1:1, which were obtained from pseudo free-standing tensile tests[7,11]. Water-soluble PSSNa sacrificial layer (thickness: ~30 nm) was firstly prepared on Si substrate, followed by directly spin-coating precursor/DPPTT-1:1 blend solutions and thermal crosslinking in nitrogen atmosphere (thickness: ~40 nm). The polymer films were first patterned into dog-bone shape according to previous reports, followed by slowly dipping into deionized water to release and float the semiconductor film by dissolving the underneath PSSNa layer. Later, the semiconductor film was hold with two aluminum tensile grips coated with a thin PDMS layer, and then tensile tests were performed. The strain rate is 0.05 s^{-1}.

Note: with the same value of precursor-to-DPPTT weight ratio, all of the films exhibit similar elastic modulus with each other, yet lower elastic modulus than neat DPPTT films, which is determined by the amount of secondary component added. BF/DPPTT doesn't show any improvement in fracture strain than DPPTT, due to the lack of energy dissipation mechanism. Simply softening DPPTT through the plasticizing effect of polybutadiene precursor cannot delay crack formation. For iRUM-s, BAc/DPPTT and BH/DPPTT, they show higher fracture strain (crack on-set strain higher than 100%) than DPPTT (crack on-set strain of 50%), due to the formation of a rubber matrix. However, the extent of improvement is different among them. BH/DPPTT shows the highest fracture strain, iRUM-s is in between, while BAc/DPPTT is the lowest. The reason lies in network structure. BH/DPPTT has one uniform network, BAc/DPPTT has double network, in which there only exists physical interaction between the two networks. In the case of iRUM-s, the BA network is chemically connected to DPPTT network. The more homogeneous of a crosslinked semiconductor film is, the higher fracture strain will be obtained.

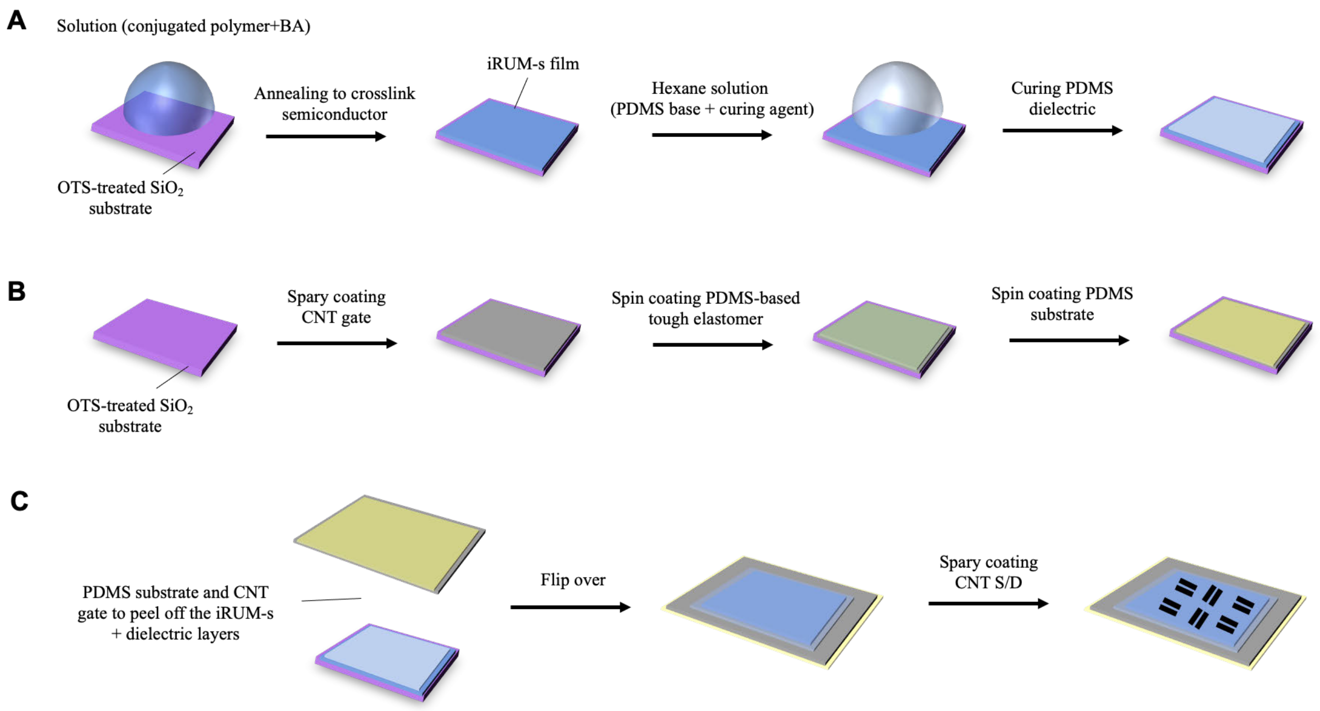

Supplementary Figure 34. Fabrication flow of a bottom-gate top-contact fully stretchable transistor. Carbon nanotube (CNT) serves as gate and source/drain electrodes (W= 1000 µm, L= 150 µm), iRUM-s as semiconductor, PDMS (2.4 µm) as dielectrics, and PDMS (1 mm) as stretchable substrate.

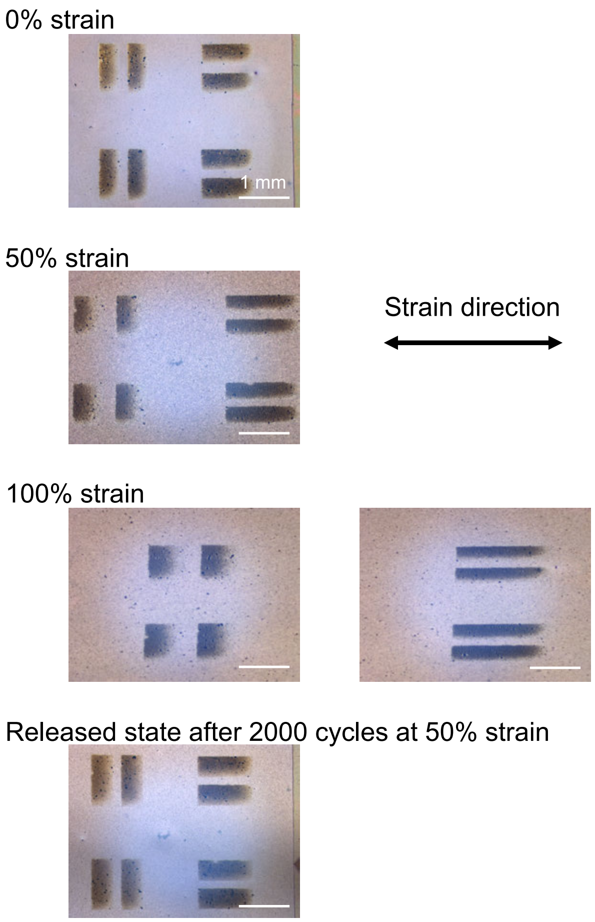

Supplementary Figure 35. Photographs of strained TFT's with source/drain electrodes both parallel and perpendicular to the strain direction. Scale bars: 1 mm.

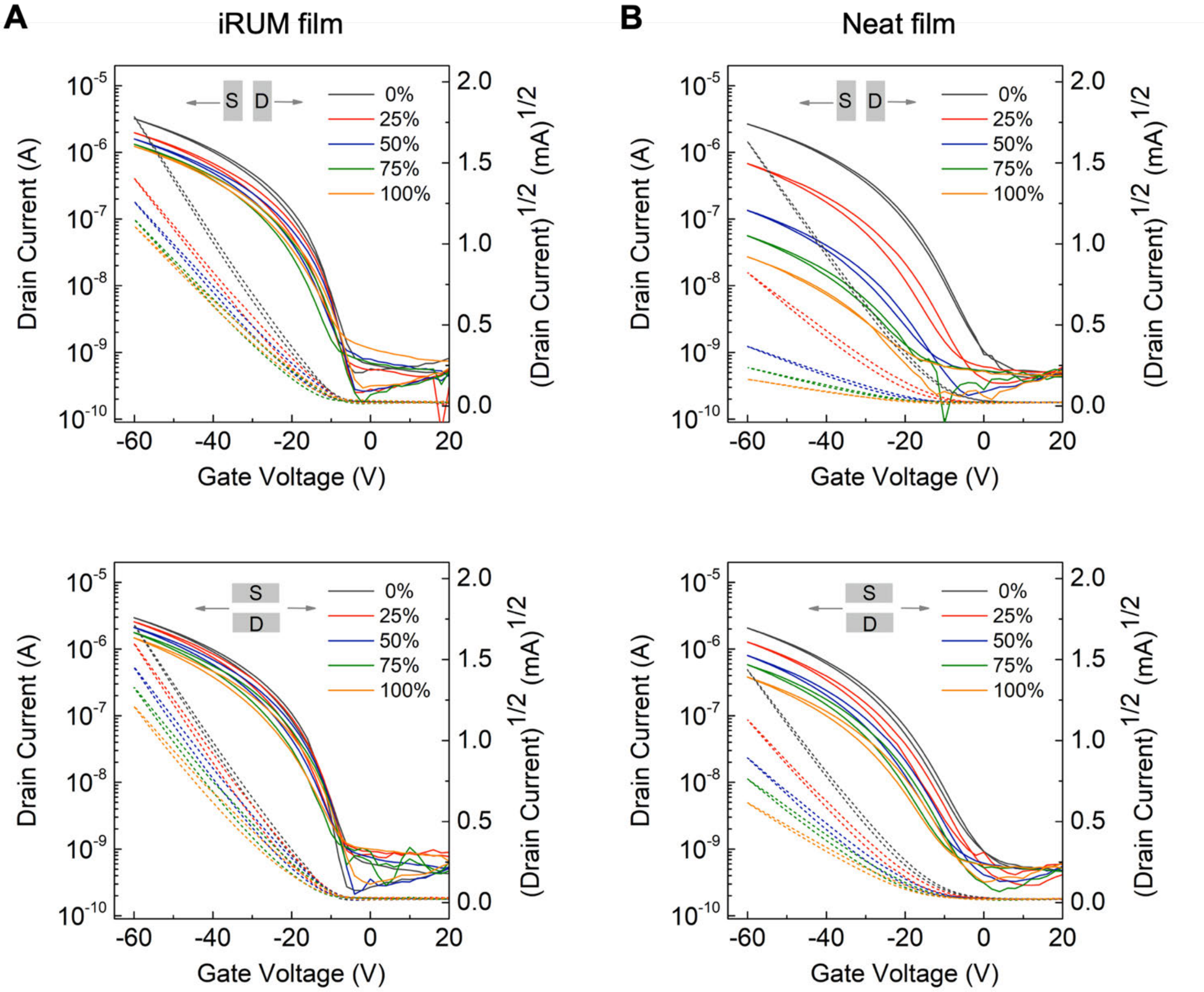

Supplementary Figure 36. Transfer curves from the bottom-gate top-contact fully stretchable transistors, with (**A**) iRUM film or (B) neat DPPTT film as semiconductor during single stretching, with charge transport parallel and perpendicular to stretching direction (solid lines: drain current; dash lines: square root of the drain current). Carbon nanotube (CNT) serves as gate and source/drain electrodes (W= 1000 μm, L= 150 μm), and PDMS (2.4 μm) as dielectrics.

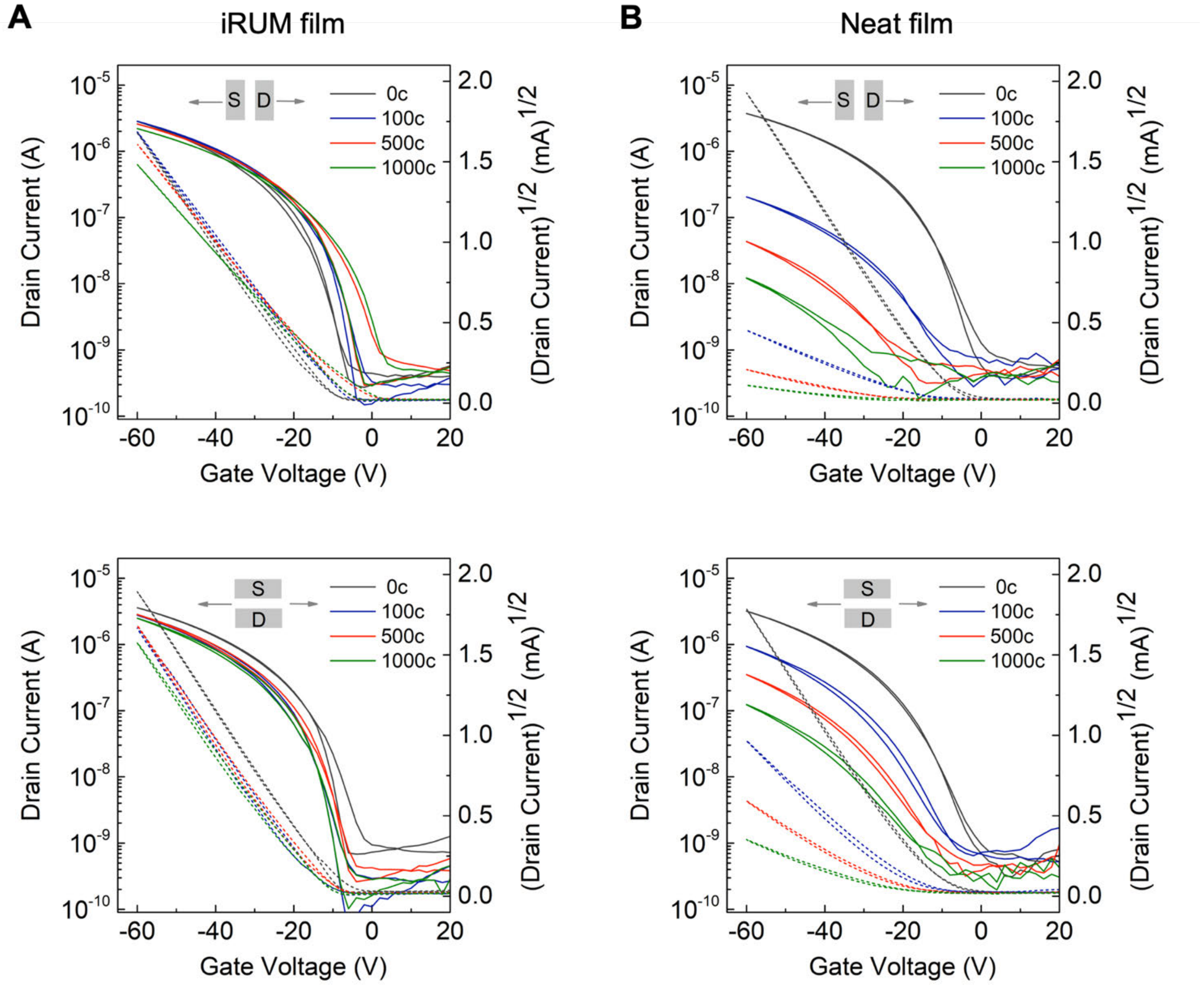

Supplementary Figure 37. Transfer curves from the bottom-gate top-contact fully stretchable transistors, with (**A**) iRUM film or (B) neat DPPTT film as semiconductor under strain released state after multiple stretching-releasing cycles at 50% strain, with charge transport parallel and perpendicular to stretching direction (solid lines: drain current; dash lines: square root of the drain current). Carbon nanotube (CNT) serves as gate and source/drain electrodes (W= 1000 μm, L= 150 μm), and PDMS (2.4 μm) as dielectrics.

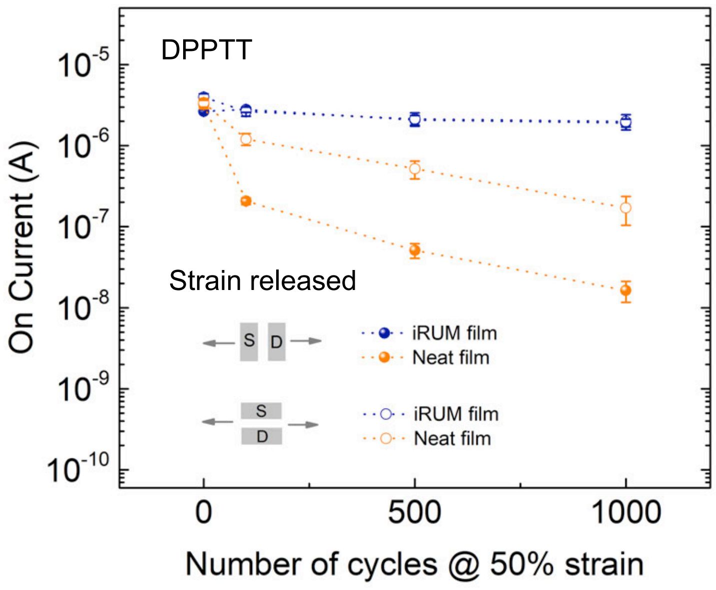

Supplementary Figure 38. Evolution in on current from the bottom-gate top-contact fully stretchable transistors, with iRUM film or neat DPPTT film as semiconductor under strain released state after multiple stretching-releasing cycles at 50% strain, with charge transport parallel and perpendicular to stretching direction. Carbon nanotube (CNT) serves as gate and source/drain electrodes (W= 1000 µm, L= 150 µm), and PDMS (2.4 µm) as dielectrics.

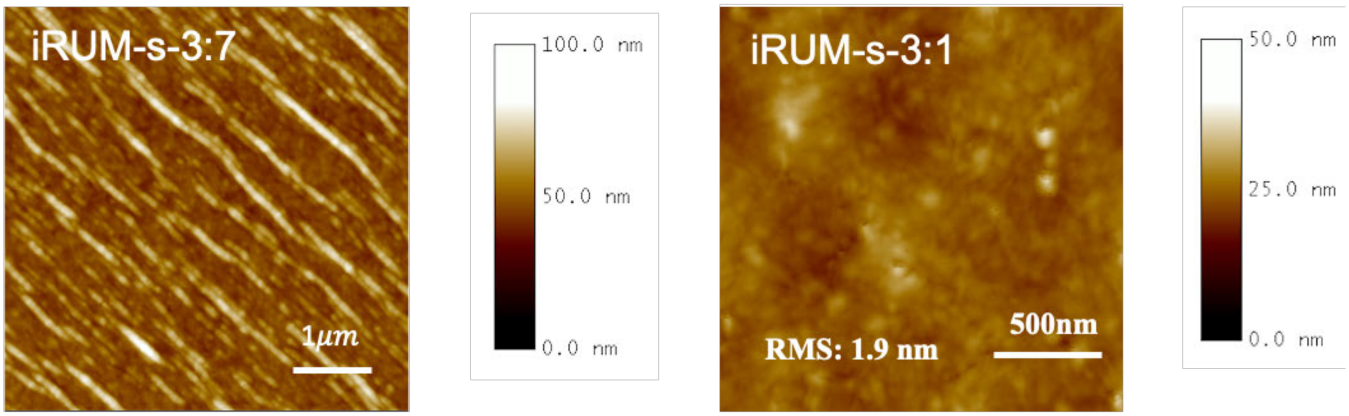

Supplementary Figure 39. AFM height images of iRUM-s-3:7 and iRUM-s-3:1 film under strain released state after 500 stretching-releasing cycles at 50% strain.

Note: iRUM-s-3:7 film still undergoes plastic deformation, thus exhibiting clear wrinkle patterns after strain released. The wrinkles cannot be observed under optical microscope; therefore, AFM is a more accurate tool for wrinkle characterization. iRUM-s-3:1 doesn't have wrinkle patterns, due to its elastic deformation behavior. Additionally, its surface roughness after cyclic stretching is similar as the spin-coated film.

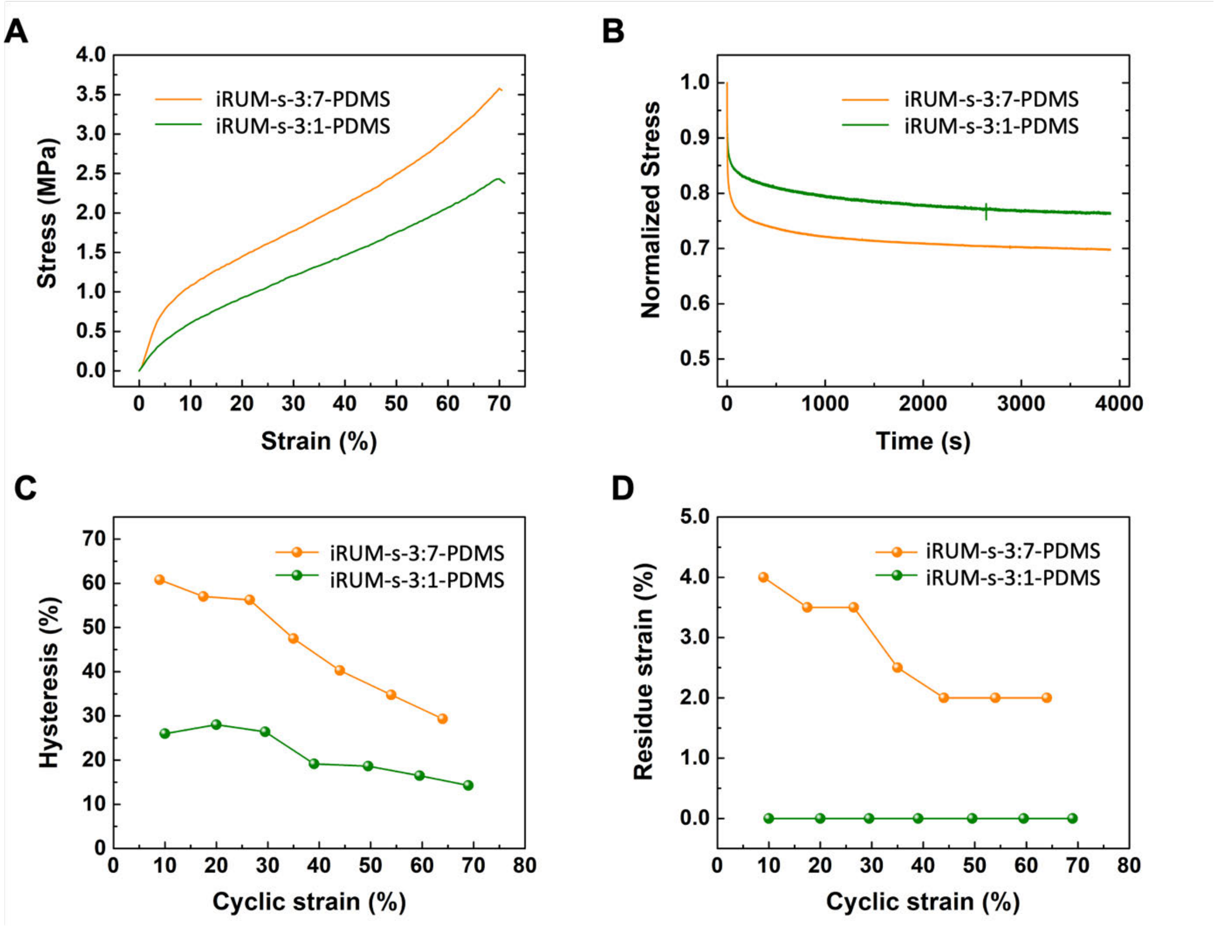

Supplementary Figure 40. (**A**) the representative stress-strain curves (**B**) stress relaxation and (**C**) hysteresis (**D**) residue strain as extracted from cyclic tests for iRUM-s-3:7 and iRUM-s-3:1 film supported by a thin PDMS layer. These were obtained from pseudo free-standing tensile tests. Water-soluble PSSNa sacrificial layer (thickness: ~30 nm) was firstly prepared on Si substrate, followed by directly spin-coating BA/DPPTT-x:y blend solutions and thermal crosslinking at 150 °C for 40 min in nitrogen atmosphere (thickness: ~40 nm). An additional thin layer of PDMS is directly prepared on top of the semiconductor film by spin-coating a dielectrics solution followed by annealing at 70 °C for 1h in air and annealing in glove box at 150 °C for 40 min (thickness ~2.4 μm). The multi-layer film was slowly dipped into deionized water to release and float the iRUM-s-x:y-PDMS bilayer film by dissolving the underneath PSSNa layer, then was hold with two aluminum tensile grips coated with a thin PDMS layer and performing tensile tests. For stress-strain measurements and cyclic tests, the strain rate is 0.05 s^{-1}. For stress relaxation, a strain rate of 0.03 s^{-1} is applied and the strain is kept within the elastic region (5%).

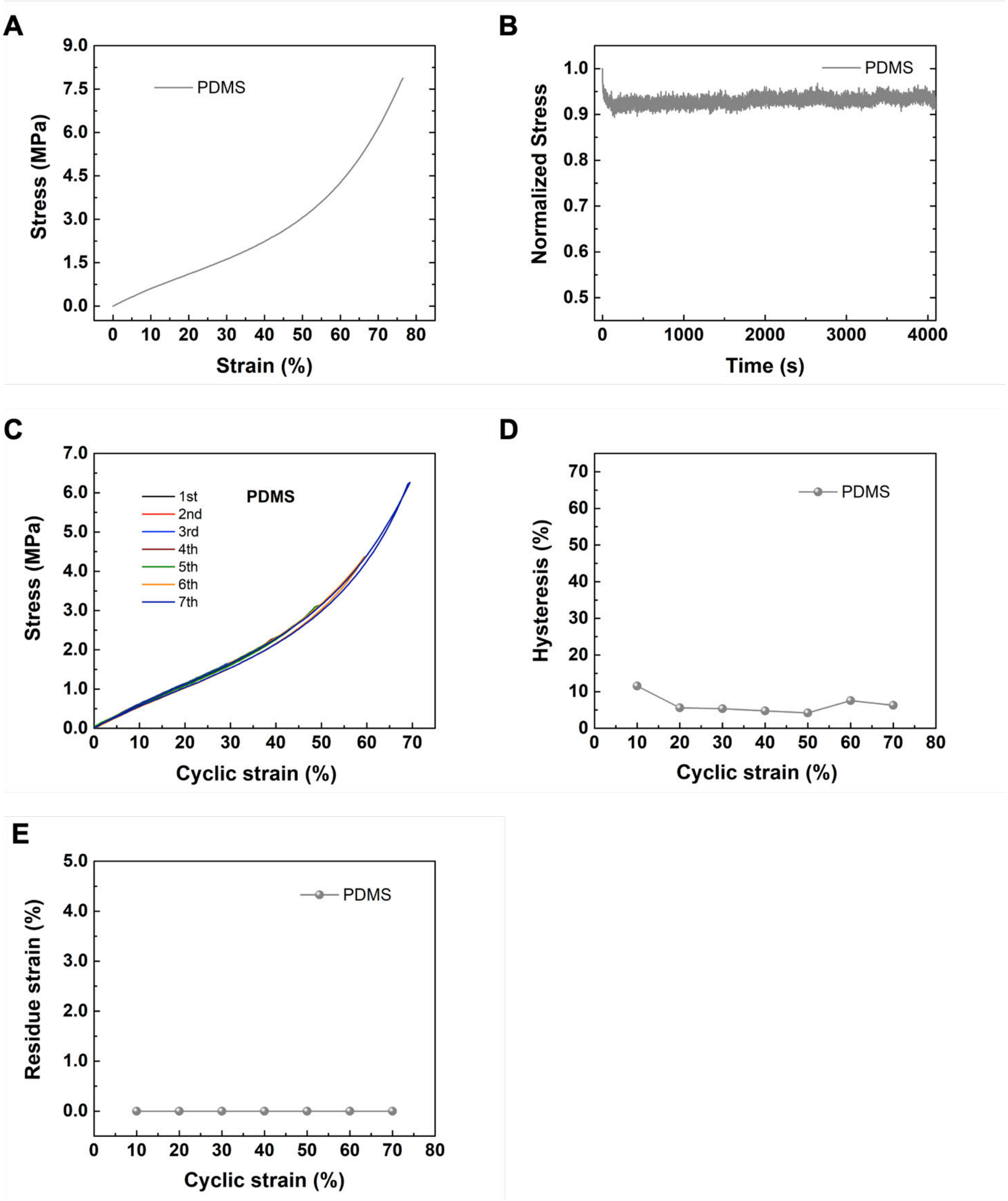

Supplementary Figure 41. Mechanical properties characterizations for PDMS thin layer (2.4 μm) (**A**) the stress-strain curve (**B**) stress relaxation (**C**) cyclic stress-strain curves and the extracted (**D**) hysteresis (**E**) residue strain, which were obtained from pseudo free-standing tensile tests.

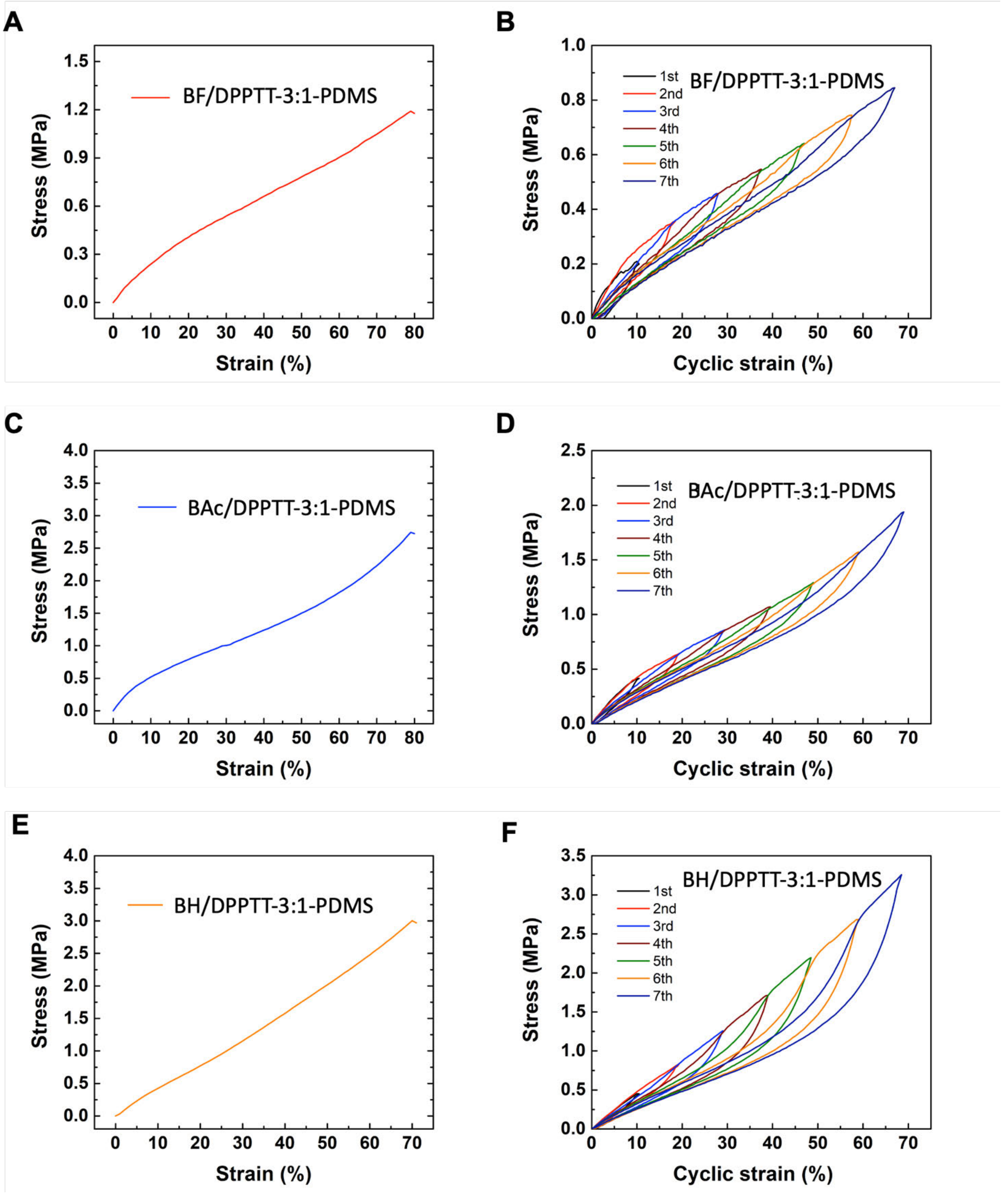

Supplementary Figure 42. The single and cyclic stress-strain curves for BF/DPPTT-3:1 blend film (**A**) and (**B**), BAc/DPPTT-3:1 crosslinked film (**C**) and (**D**), BH/DPPTT-3:1 crosslinked film (**E**) and (**F**), supported on a thin PDMS layer. These were obtained from pseudo free-standing tensile tests. The resultant thickness for semiconductor film (BF/DPPTT-3:1 blend film, BAc/DPPTT-3:1 crosslinked film and BH/DPPTT-3:1 crosslinked film) is around 40nm, while the thickness for dielectrics is around 2.4 μm.

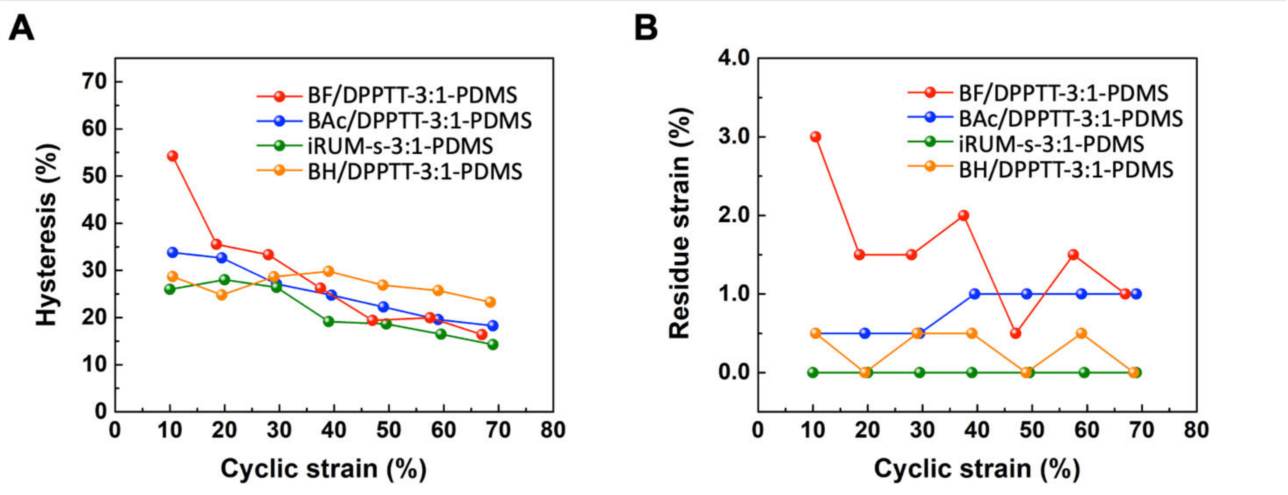

Supplementary Figure 43. Comparison of (**A**) hysteresis (**B**) residue strain as extracted from cyclic stress-strain curves for BF/DPPTT-3:1, BAc/DPPTT-3:1, iRUM-s-3:1 and BH/DPPTT-3:1 film supported on a thin PDMS layer. These were obtained from pseudo free-standing tensile tests. The resultant thickness for semiconductor film (BF/DPPTT-3:1 blend film, BAc/DPPTT-3:1 crosslinked film, iRUM-s-3:1 and BH/DPPTT-3:1 crosslinked film) is around 40nm, while the thickness for dielectrics is around 2.4 μm.

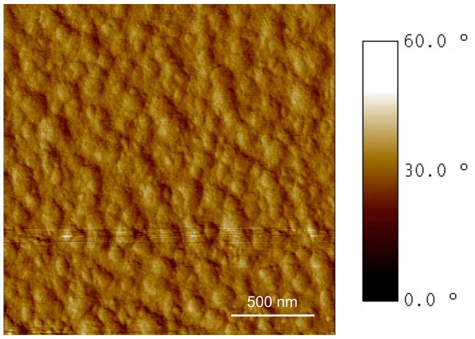

Supplementary Figure 44. AFM phase image of iRUM-s-IDTBT film.

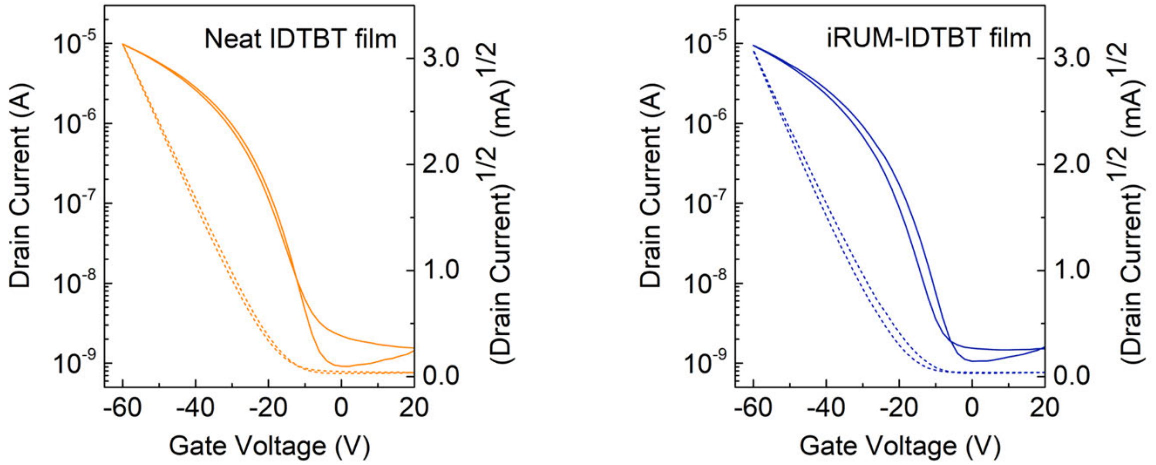

Supplementary Figure 45. Transfer curves from the bottom-gate top-contact fully stretchable transistors, with neat IDTBT film or iRUM-IDTBT film as semiconductor (BA/IDTBT=3:7). The extracted average mobility is 1.66 cm^2 V^{-1} s^{-1} for iRUM film and 1.64 cm^2 V^{-1} s^{-1} for neat film (averaged more than 10 devices). The electrical performance of IDTBT is well maintained after applying this iRUM approach. Carbon nanotube (CNT) serves as gate and source/drain electrodes (W= 1000 µm, L= 150 µm), and PDMS (2.4 µm) as dielectrics.

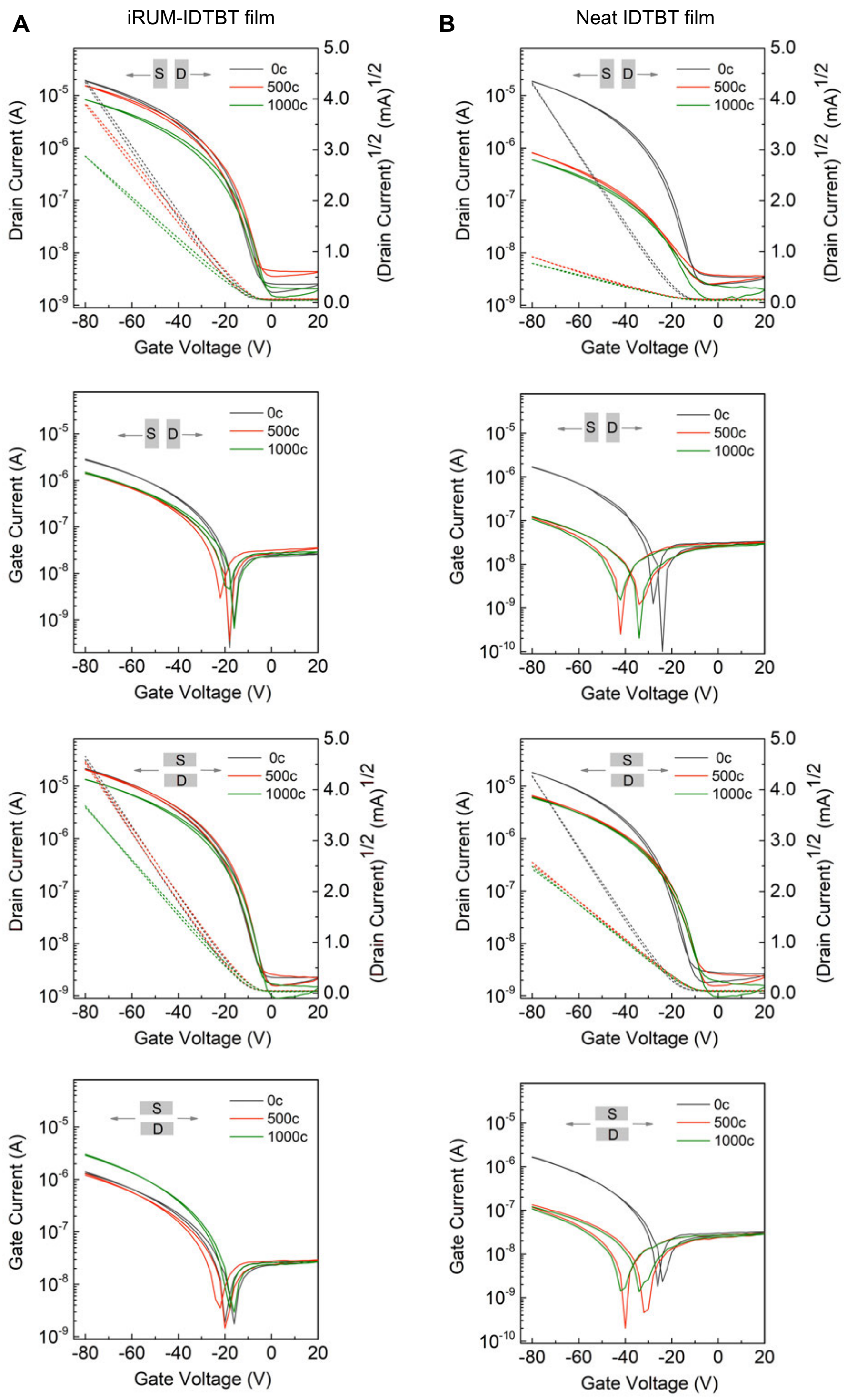

Supplementary Figure 46. Transfer curves from the bottom-gate top-contact fully stretchable transistors, with (**A**) iRUM-IDTBT film (BA/IDTBT=3:7) or (**B**) neat IDTBT film as semiconductor under strain released state after multiple stretching-releasing cycles at 50% strain, with charge transport parallel and perpendicular to stretching direction (solid lines: drain/gate current; dash lines: square root of the drain current). Carbon nanotube (CNT) serves as gate and source/drain electrodes (W= 1000 μm, L= 150 μm), and PDMS (2.4 μm) as dielectrics.

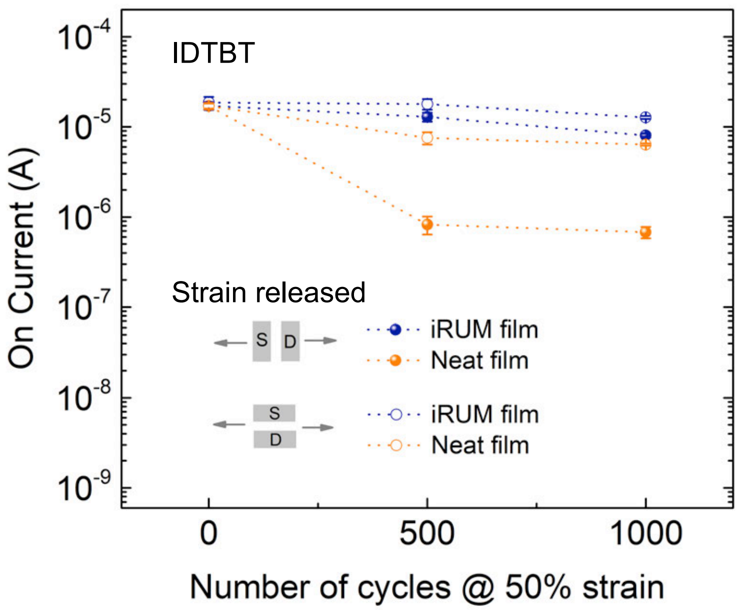

Supplementary Figure 47. Evolution in on current from the bottom-gate top-contact fully stretchable transistors, with iRUM film or neat IDTBT film as semiconductor under strain released state after multiple stretching-releasing cycles at 50% strain, with charge transport parallel and perpendicular to stretching direction. Carbon nanotube (CNT) serves as gate and source/drain electrodes (W= 1000 μm, L= 150 μm), and PDMS (2.4 μm) as dielectrics.

Note: the high gate leakage current of stretchable transistors results from the transistor structure and fabrication flow design (Supplementary Figure S34). The PDMS dielectric solution was spin-coated and cured directly on top of semiconductor. Therefore, the dielectric layer is of the same size as the semiconductor layer. After transferring semiconductor/dielectric together with CNT gate layer, there is a large overlapping area between the semiconductor and the CNT gate, thus resulting in high gate leakage current. However, this doesn't affect the extracted mobility of the semiconductor much. For future circuit fabrication, both the semiconductor and the gate will be patterned into small structures to minimize overlapping area to minimize leakage current.

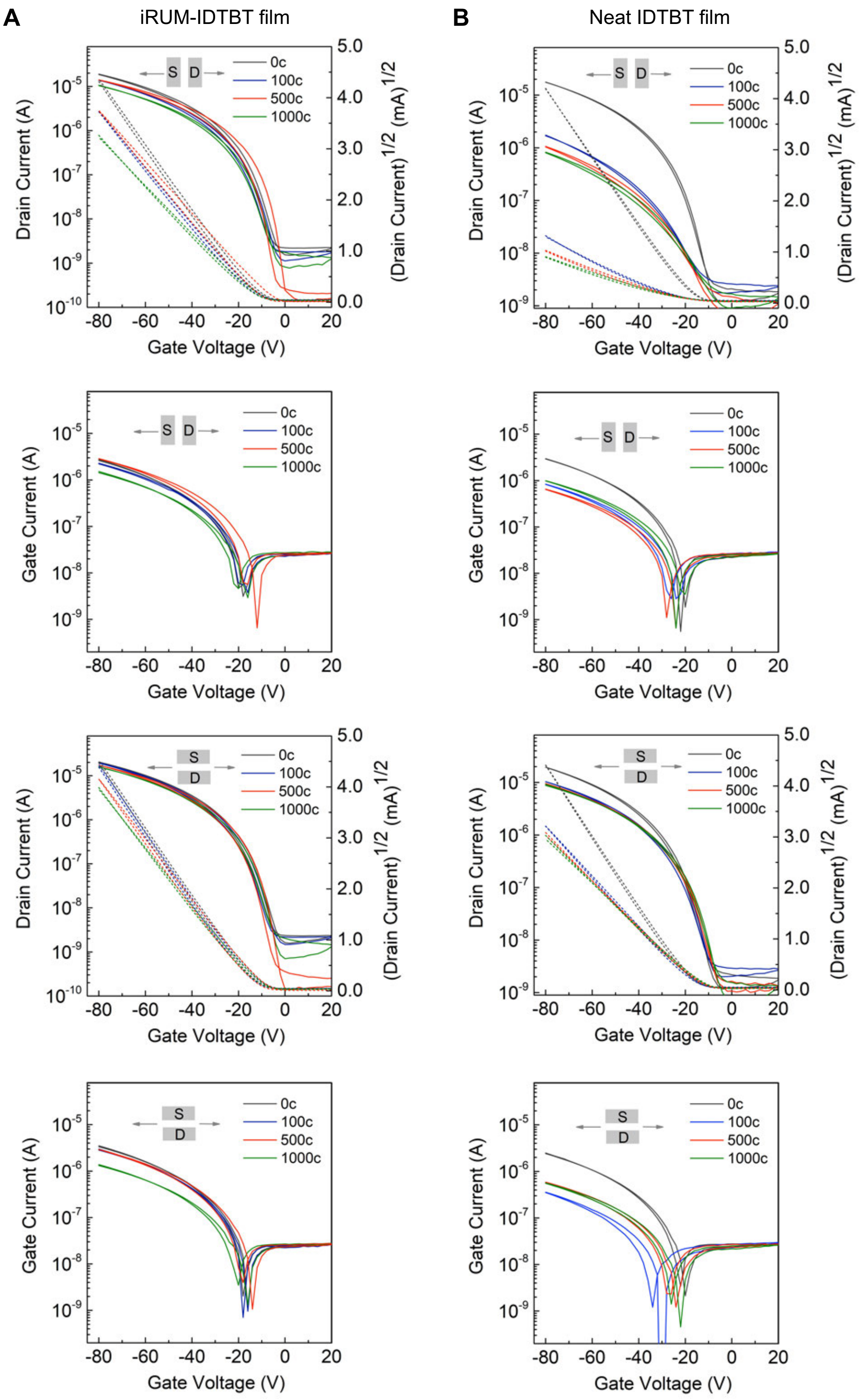

Supplementary Figure 48. Transfer curves from the bottom-gate top-contact fully stretchable transistors, with (**A**) iRUM-IDTBT film (BA/IDTBT=3:7) or (B) neat IDTBT film as semiconductor under strain released state after multiple stretching-releasing cycles at 25% strain, with charge transport parallel and perpendicular to stretching direction (solid lines: drain/gate current; dash lines: square root of the drain current). Carbon nanotube (CNT) serves as gate and source/drain electrodes (W= 1000 μm, L= 150 μm), and PDMS (2.4 μm) as dielectrics.

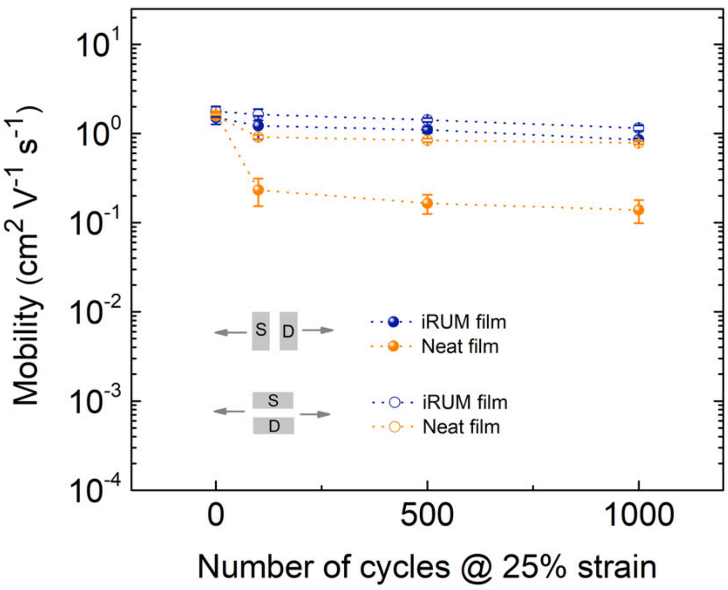

Supplementary Figure 49. Changes in mobility of a neat IDTBT film and an iRUM-IDTBT (BA/IDTBT=3:7) film after multiple stretching-releasing cycles at 25% strain under strain released state. IDTBT suffers from nearly one order drop in mobility even after cycling at 25% strain, which is due to plastic deformation.

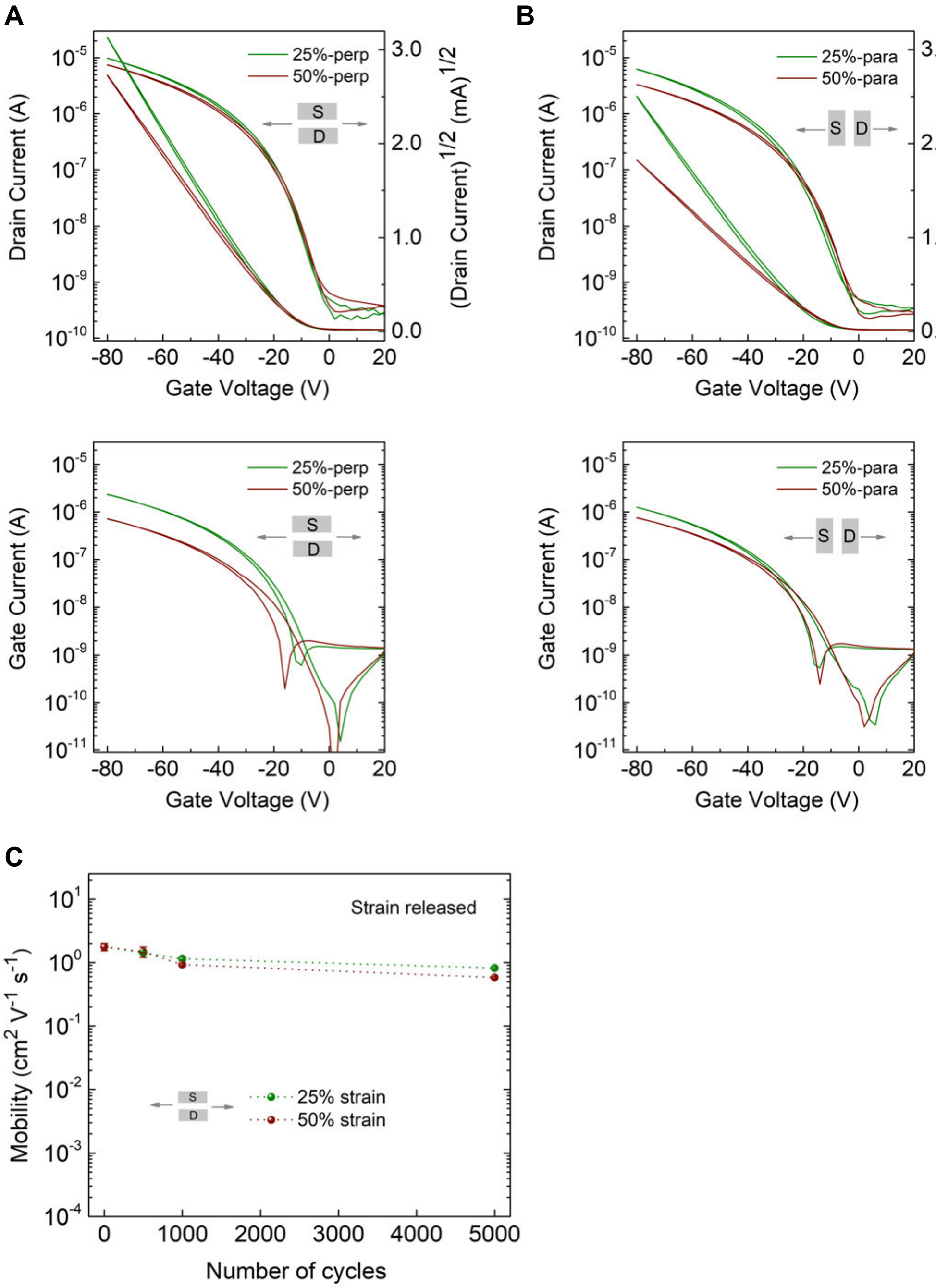

Supplementary Figure 50. Transfer curves from the bottom-gate top-contact fully stretchable transistors, with iRUM-IDTBT film (BA/IDTBT=3:7) as semiconductor under strain released state after multiple stretching-releasing cycles at 25% and 50% strain, with charge transport (**A**) perpendicular and (**B**) parallel to stretching direction (solid lines: drain/gate current; dash lines: square root of the drain current). (**C**) Changes in mobility of an iRUM-IDTBT (BA/IDTBT=3:7) film after multiple stretching-releasing cycles at 25% and 50% strain under strain released state. Carbon nanotube (CNT) serves as gate and source/drain electrodes (W= 1000 μm, L= 150 μm), and PDMS (2.4 μm) as dielectrics.

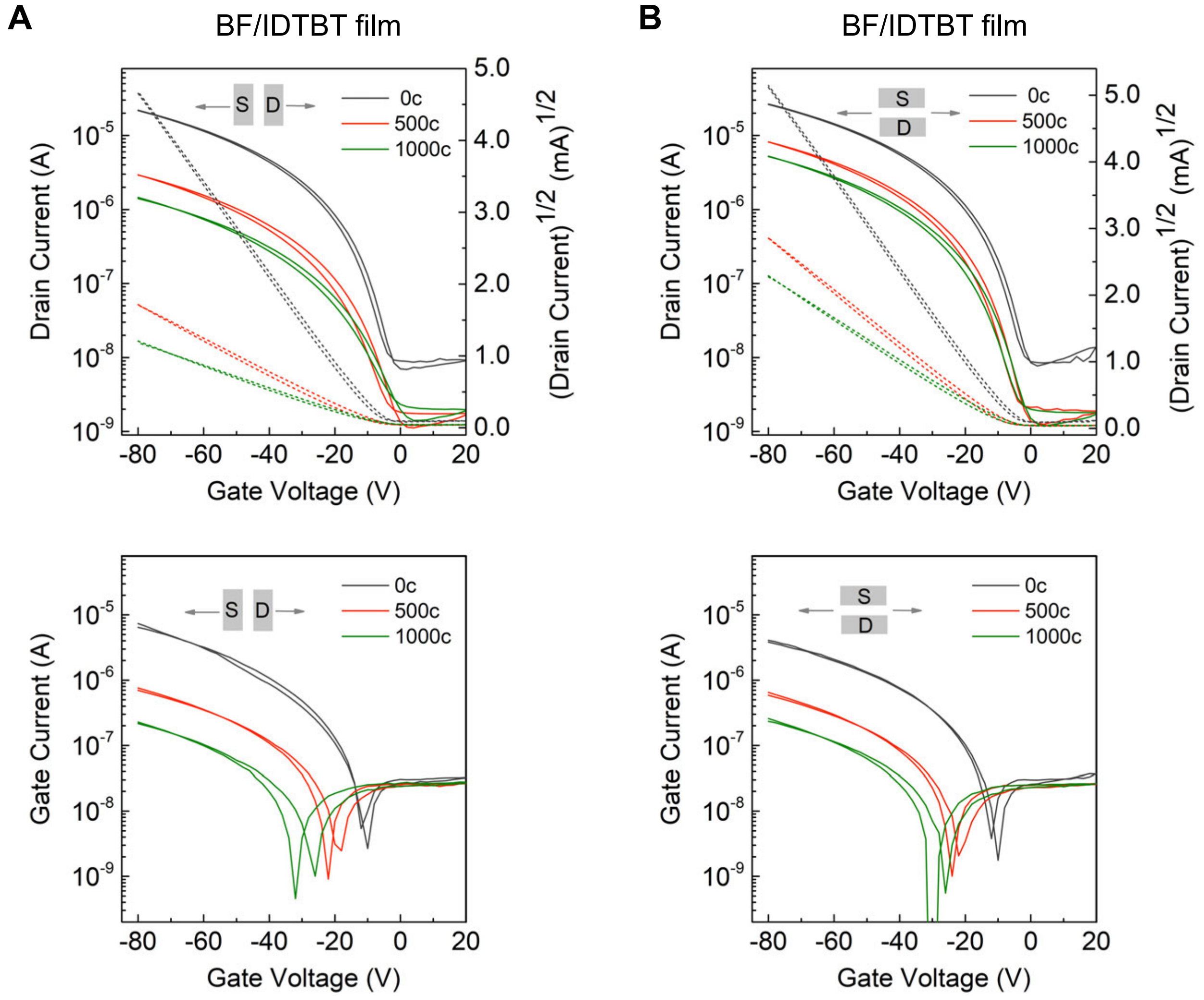

Supplementary Figure 51. Transfer curves from the bottom-gate top-contact fully stretchable transistors, with BF/IDTBT (3:7) blend film as semiconductor under strain released state after multiple stretching-releasing cycles at 50% strain, with charge transport (**A**) parallel and (**B**) perpendicular to stretching direction (solid lines: drain/gate current; dash lines: square root of the drain current). IDTBT/BF blend semiconductor film exhibits similar mobility as neat IDTBT film. Carbon nanotube (CNT) serves as gate and source/drain electrodes (W= 1000 μm, L= 150 μm), and PDMS (2.4 μm) as dielectrics.

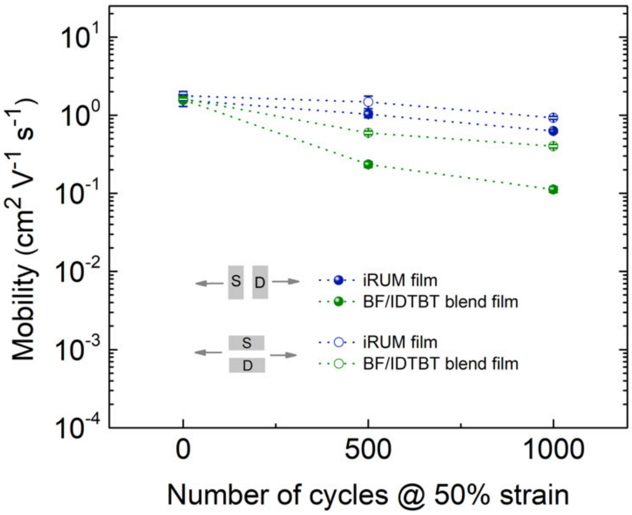

Supplementary Figure 52. Changes in mobility of a BF/DTBT blend film and an iRUM-IDTBT film after multiple stretching-releasing cycles at 50% strain under strain released state.

Note: Due to the polybutadiene backbone structure, interfacial crosslinking can be created between BF/IDTBT blend semiconductor film and PDMS dielectrics through hydrosilylation during Si–H/vinyl curing process. In addition, due to the plasticizing effect, BF/IDTBT exhibits reduced elastic modulus compared to IDTBT. However, BF/IDTBT blend film still suffers from mobility drop after multiple stretching-releasing cycles, which confirms that the cyclic durability cannot be improved through simply softening IDTBT with plasticizer or form interfacial crosslinking with PDMS dielectrics. BF/IDTBT blend film still undergoes plastic deformation under strain, which is similar as IDTBT film. Therefore, the improvement in cyclic durability of iRUM-IDTBT originates from its improved elasticity.

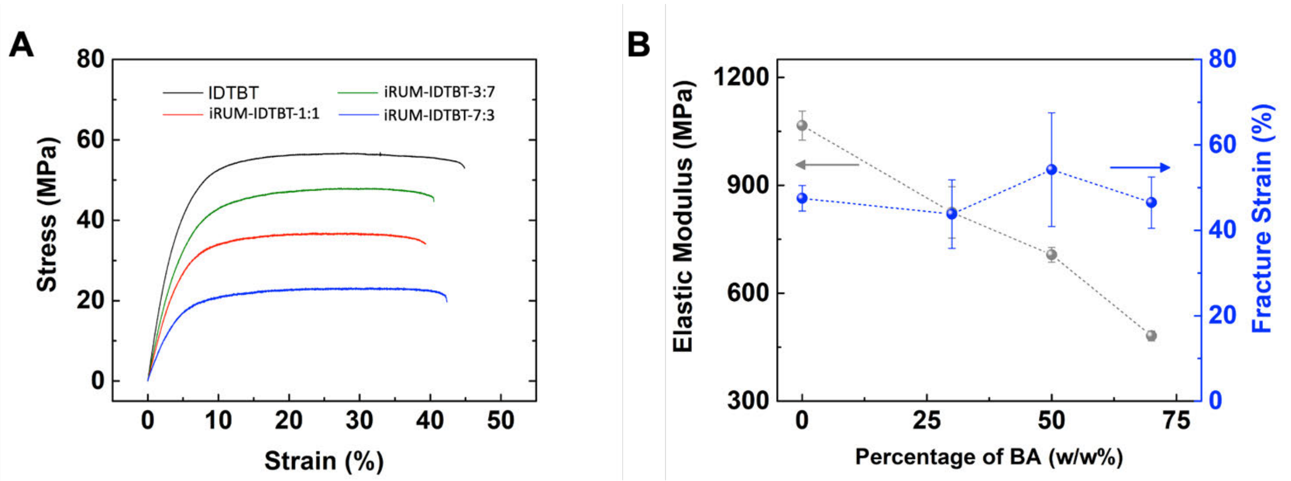

Supplementary Figure 53. The representative stress-strain curves and extracted mechanics parameters for IDTBT and iRUM-IDTBT-x:y films, which were obtained from pseudo free-standing tensile tests.

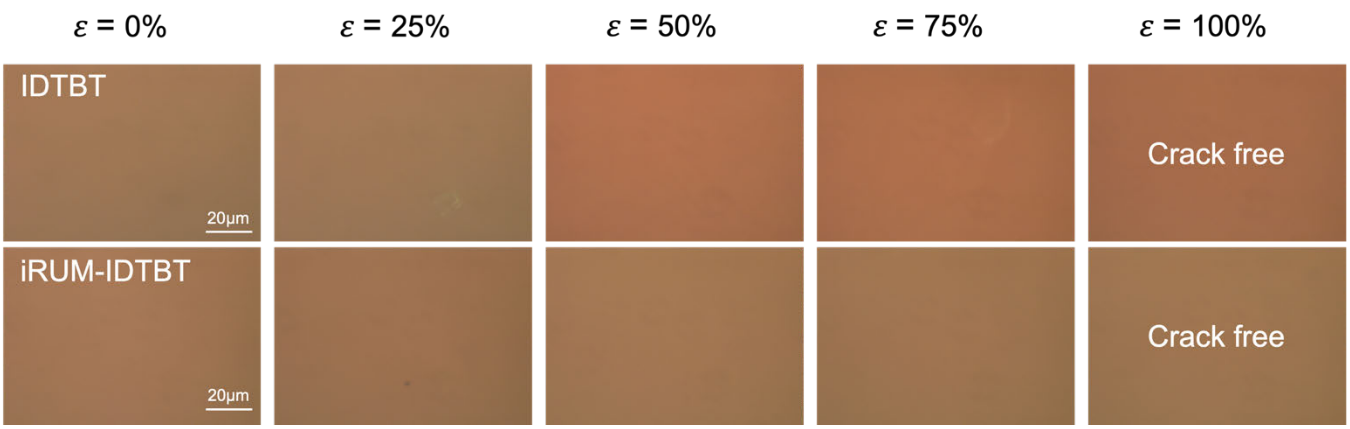

Supplementary Figure 54. Crack on-set strains of IDTBT and iRUM-IDTBT film, as measured by "film-on-elastomer". PDMS (base:crosslinker =12:1 in weight ratio).

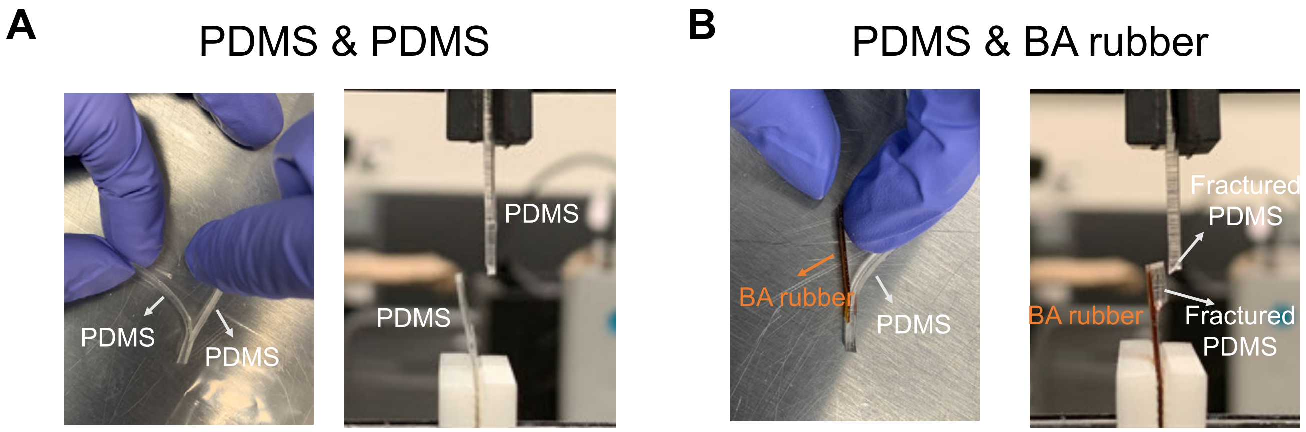

Supplementary Figure 55. 180º Peeling tests confirmed the existence of interfacial crosslinking between BA rubber and PDMS. The free-standing crosslinked BA rubber (thickness: 0.62 mm) and PDMS (thickness: 0.65 mm) are prepared first, followed by drop-casting PDMS (base/crosslinker-6:1 in weight) onto a designed region and curing at 70 ºC overnight (thickness: 1.1 mm). The interface of (**A**) PDMS & PDMS and (B) PDMS & BA crosslinked rubber. Two pieces of PDMS can be readily separated, with each piece remaining intact. For PDMS/BA rubber interface, the PDMS part even fractured by itself before the breakage of BA/PDMS interface. This indicates that the interfacial toughness between PDMS and BA crosslinked rubber is even higher than the toughness of PDMS, which is due to the formation of interfacial crosslinking. On the contrary, no interfacial crosslinking was created between two pieces of PDMS, thus low interfacial toughness.

Supplementary Movie 1. 180º Peeling test movie showing the existence of interfacial crosslinking between BA rubber and PDMS.

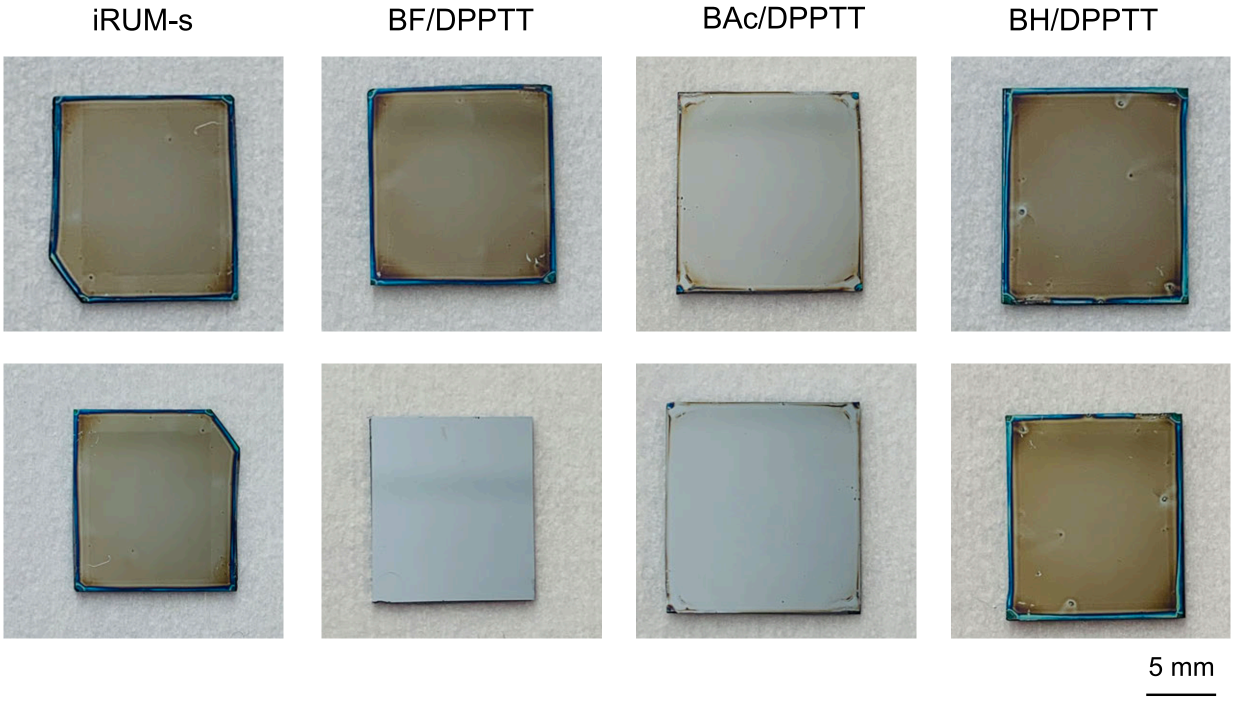

Supplementary Figure 56. Images of iRUM-s film, BF/DPPTT blend film, BAc/DPPTT and BH/DPPTT crosslinked film before (top line) and after (bottom line) soaking in chloroform for 30s.

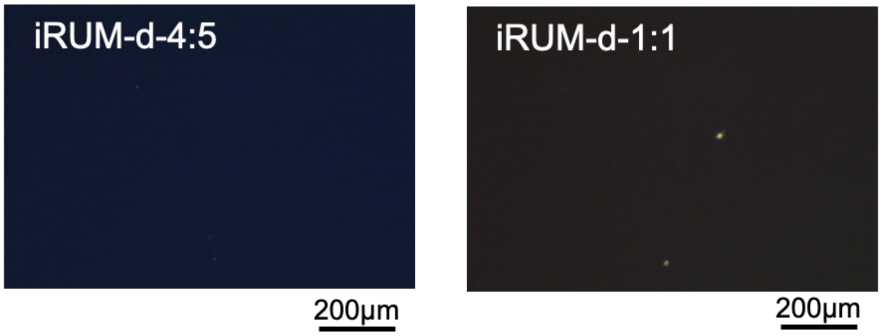

Supplementary Figure 57. Dark field optical microscope images of iRUM-d-4:5 and iRUM-d-1:1, with no large-domain phase separation observed under optical microscope, indicating that BH can mix well SEBS.

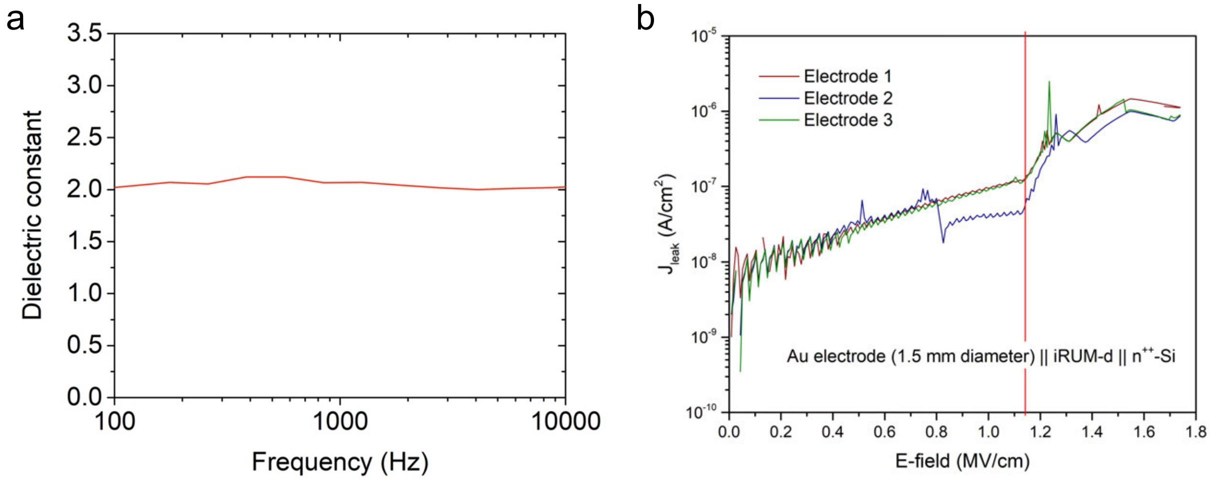

Supplementary Figure 58. (a) Dielectric constant of iRUM-d-4:5 (thickness: 1.2 μm) under the frequency between 100 and 10000 Hz. (b) Current density versus electric field (J−E) curves of iRUM-d-4:5 (thickness: 1.2 μm) showing its excellent insulating performance with low leakage current density even at 1 MV/cm.

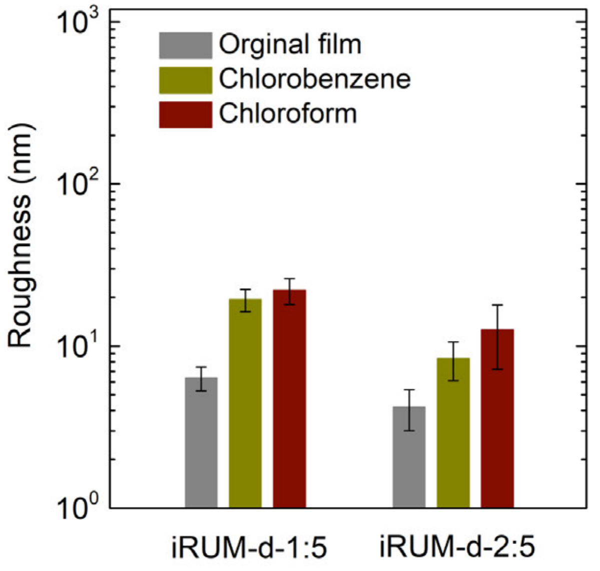

Supplementary Figure 59. Surface roughness characterization of iRUM-d-1:5 and iRUM-d-2:5 films by profilometer before- and after-solvent treatment (depositing chlorobenzene or chloroform on film surface and stay for 10s, followed by spin-coating at 1500 r.p.m. for 1 min). Compared to pristine SEBS film which showed substantial swelling after solvent treatment (roughness increased from 7 nm to 178 nm), iRUM-d-1:5 and iRUM-d-2:5 showed less swelling. However, the surface roughness still increased from 6 nm to 22 nm for iRUM-d-1:5 film, and 4 nm to 13 nm for iRUM-d-2:5 film. This explains the relatively low charge carrier mobility after depositing and photo-patterning of iRUM-s film on top of them compared to iRUM-d-4:5 film. Therefore, increasing the crosslinking density in SEBS dielectrics resulted in improved solvent-resistance and more smooth film surface after multiple solvent washing steps.

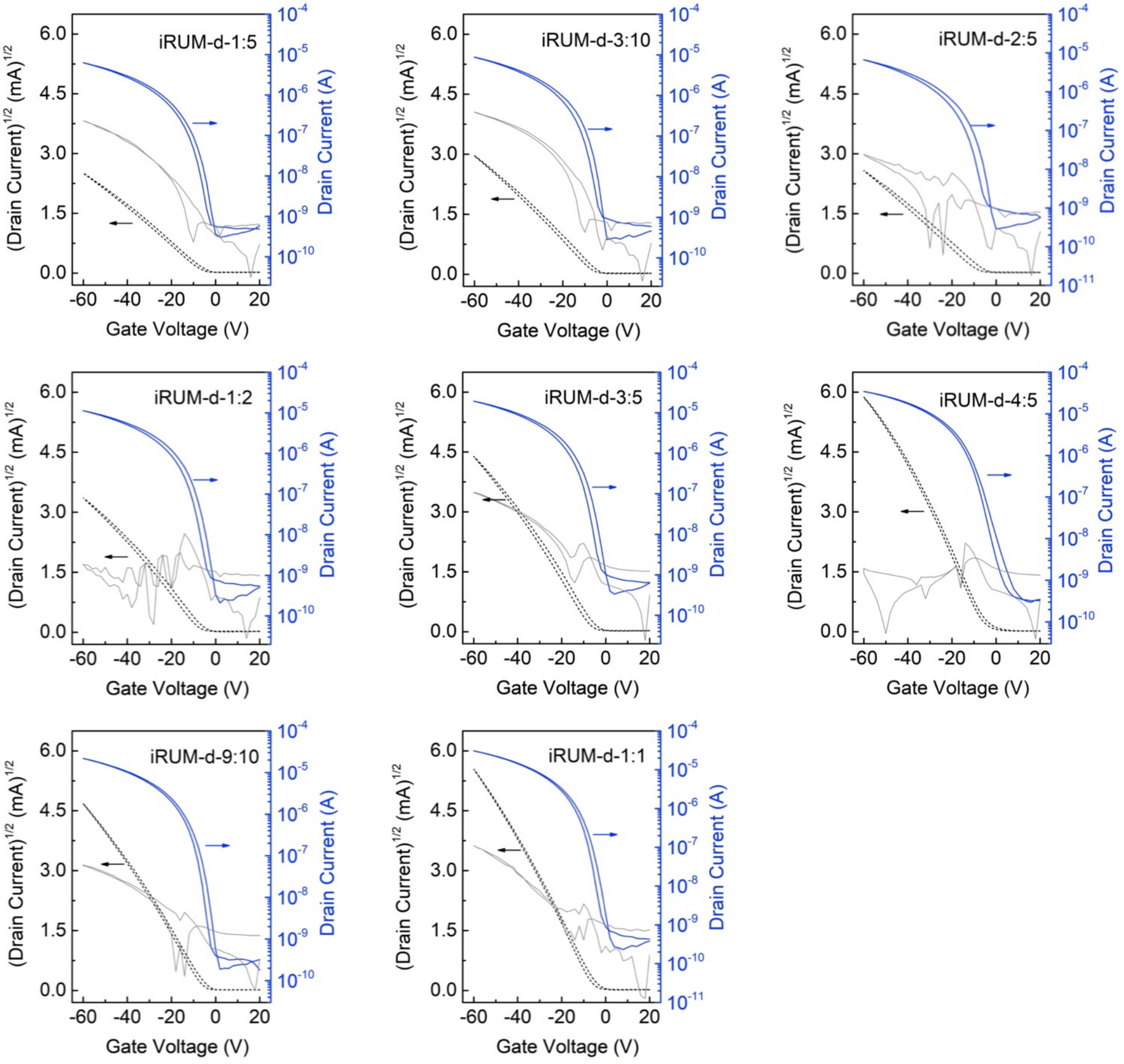

Supplementary Figure 60. Transfer curves from the bottom-gate top-contact transistors, with iRUM-s directly photo-patterned on iRUM-d-x:y, highly-doped Si as gate electrode and MoO_3/Au as source/drain electrodes (W= 1000 µm , L= 50 µm) (solid lines: drain current; dash lines: square root of the drain current).

Supplementary Table 6. Thickness and the calculated dielectric capacitance of iRUM-d-x:y.

iRUM-d-x:y	1:5	3:10	2:5	1:2	3:5	4:5	9:10	1:1
Thickness (µm)	1.5	1.4	1.3	1.2	1.1	1	1.15	1.1
Capacitance (nF/cm^2)	1.24	1.33	1.43	1.55	1.69	1.86	1.62	1.69

Note: dielectric constant is 2.1.

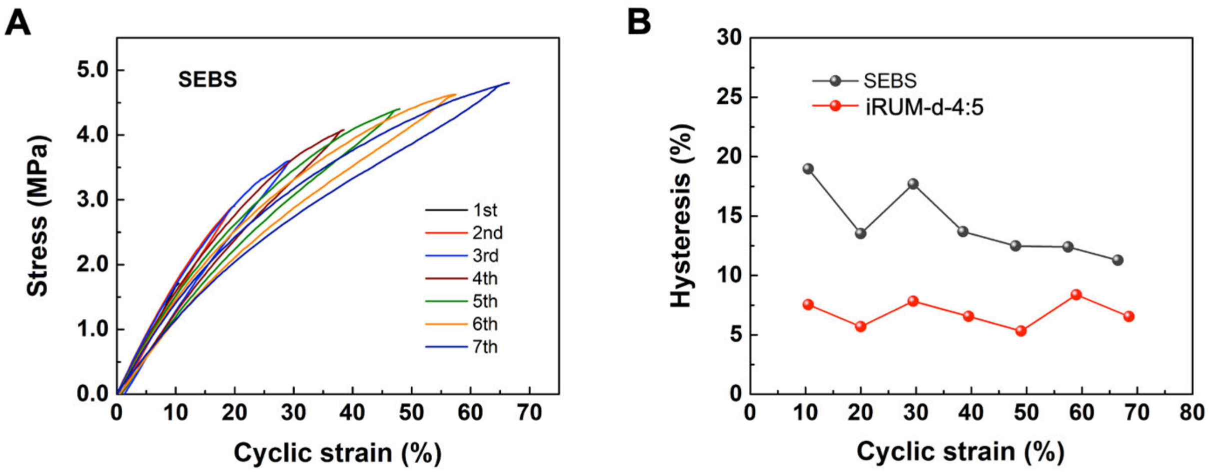

Supplementary Figure 61. (**A**) Cyclic stress-strain curves of SEBS (1.2 µm thickness). The comparison of hysteresis as extracted from cyclic stress-strain curves for SEBS and iRUM-d-4:5 (1.2 µm thickness). These were obtained from pseudo free-standing tensile tests.

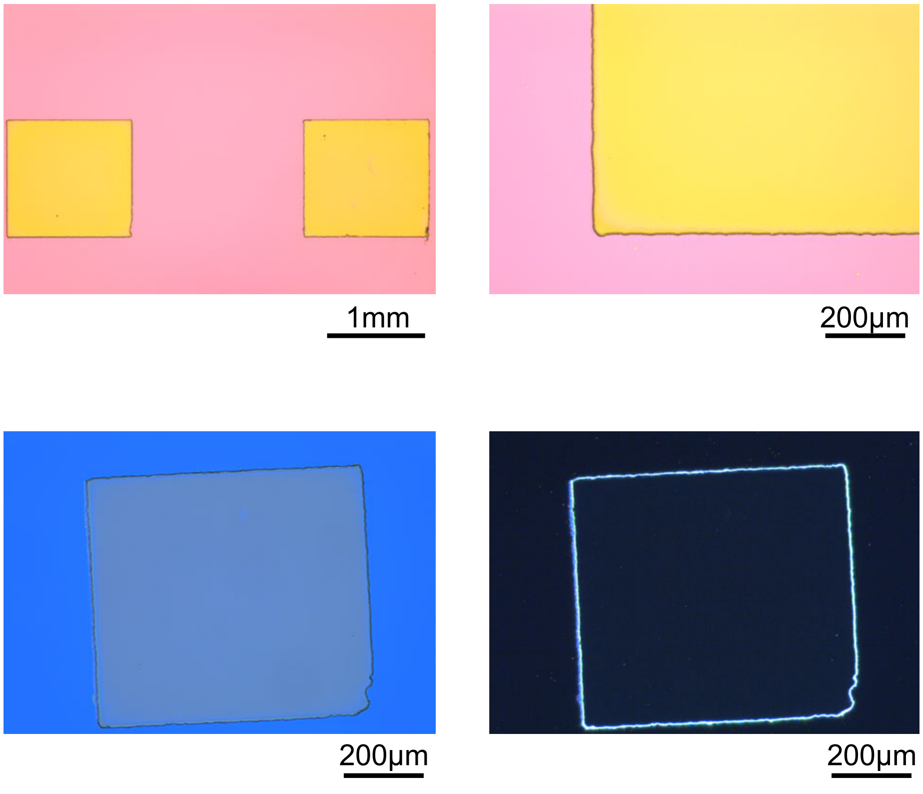

Supplementary Figure 62. Bright field and dark field optical microscope images of photo-patterned iRUM-d-4:5 using two kinds of shadow mask with different feature sizes. UV exposure for 30min, develop in dodecane and post annealing in glove box at 200 °C for 1 h. No swelling and phase separation can be observed under dark field.

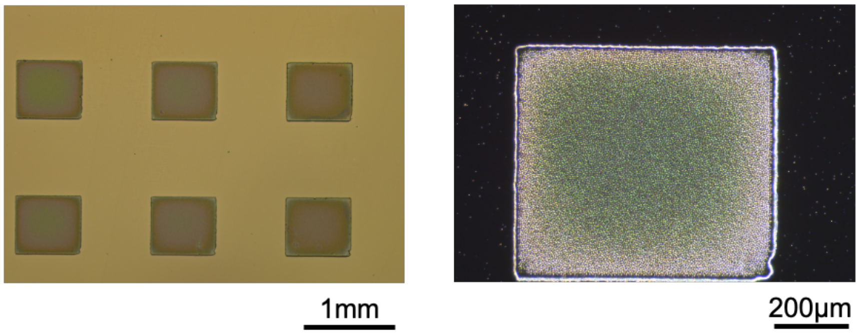

Supplementary Figure 63. Bright field and dark field optical microscope images of photo-patterned iRUM-d-4:5 film without sufficient UV exposure time (8 min). Clear swelling and phase separation can be observed under dark field.

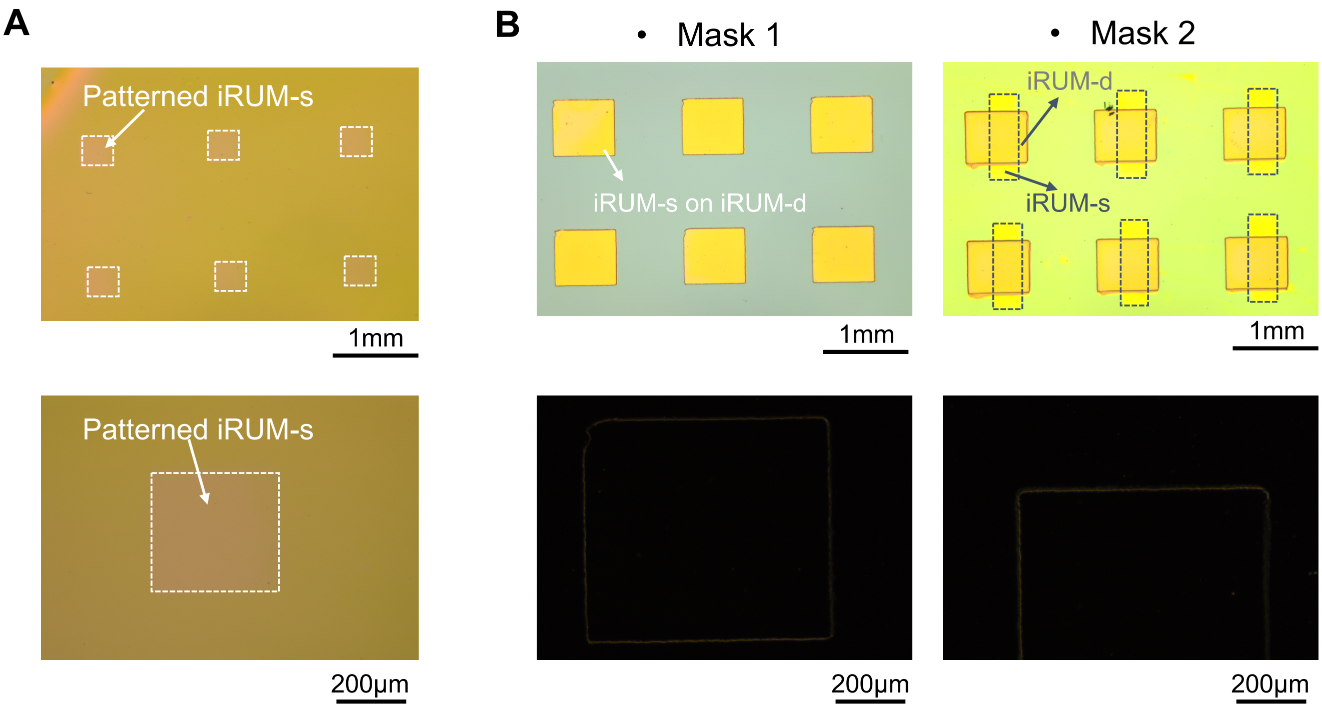

Supplementary Figure 64. Bright field and dark field optical microscope images of photo patterning iRUM-s directly on top of (**A**) unpatterned iRUM-d (**B**) photo-patterned iRUM-d. Mask 1 and 2 are the shadow masks for photo-patterning iRUM-s with different feature sizes. For mask 1, the size of patterned iRUM-s film is smaller than patterned iRUM-d film, cannot see clearly under optical microscope due to color similarity. For mask 2, the rectangle with dash edge represents photo-patterned iRUM-s, while the square represents photo-patterned iRUM-d. Therefore, iRUM-s can be successfully photo-patterned on top of iRUM-d (unpatterned and patterned) without observing swelling under dark field.

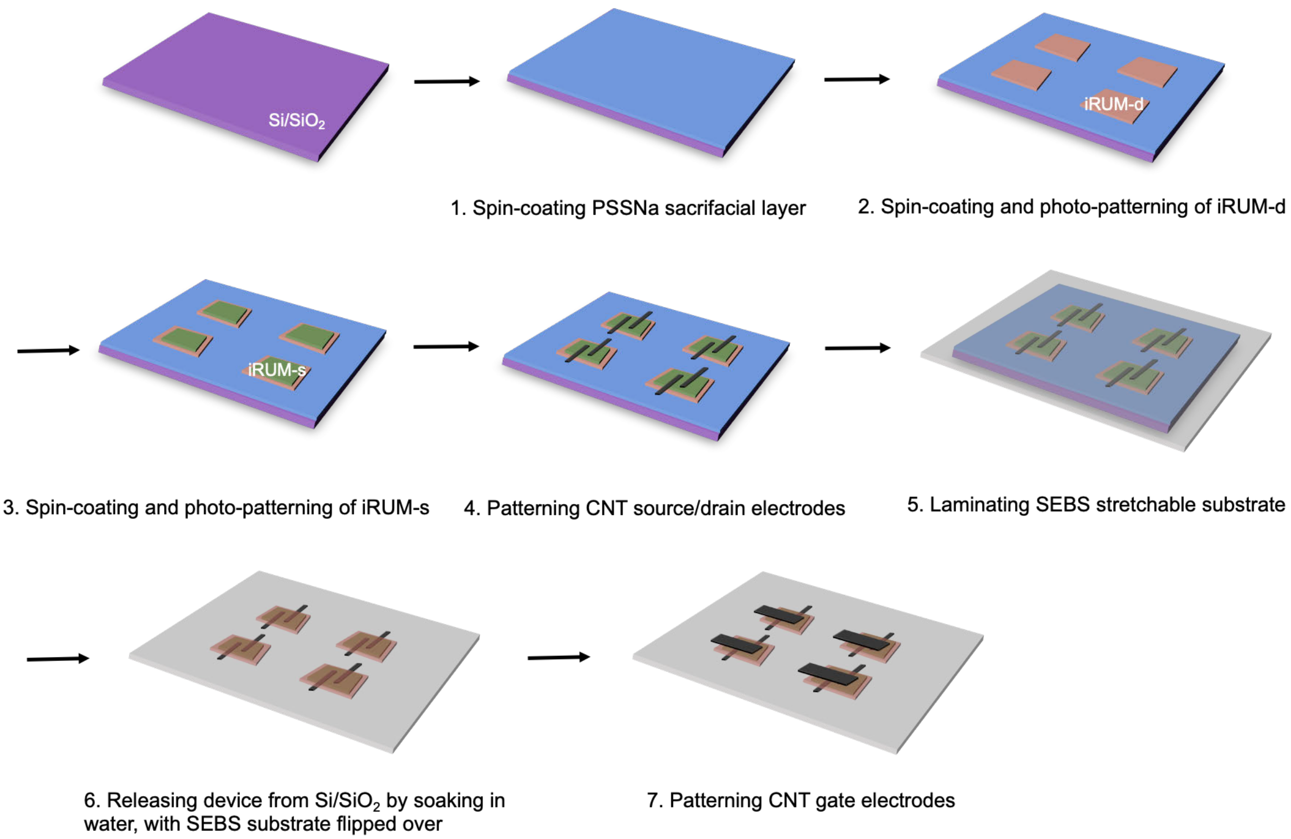

Supplementary Figure 65. Fabrication flow of a fully patterned transistor array, with iRUM-s and iRUM-d incorporated through consequently photo-patterning processes.

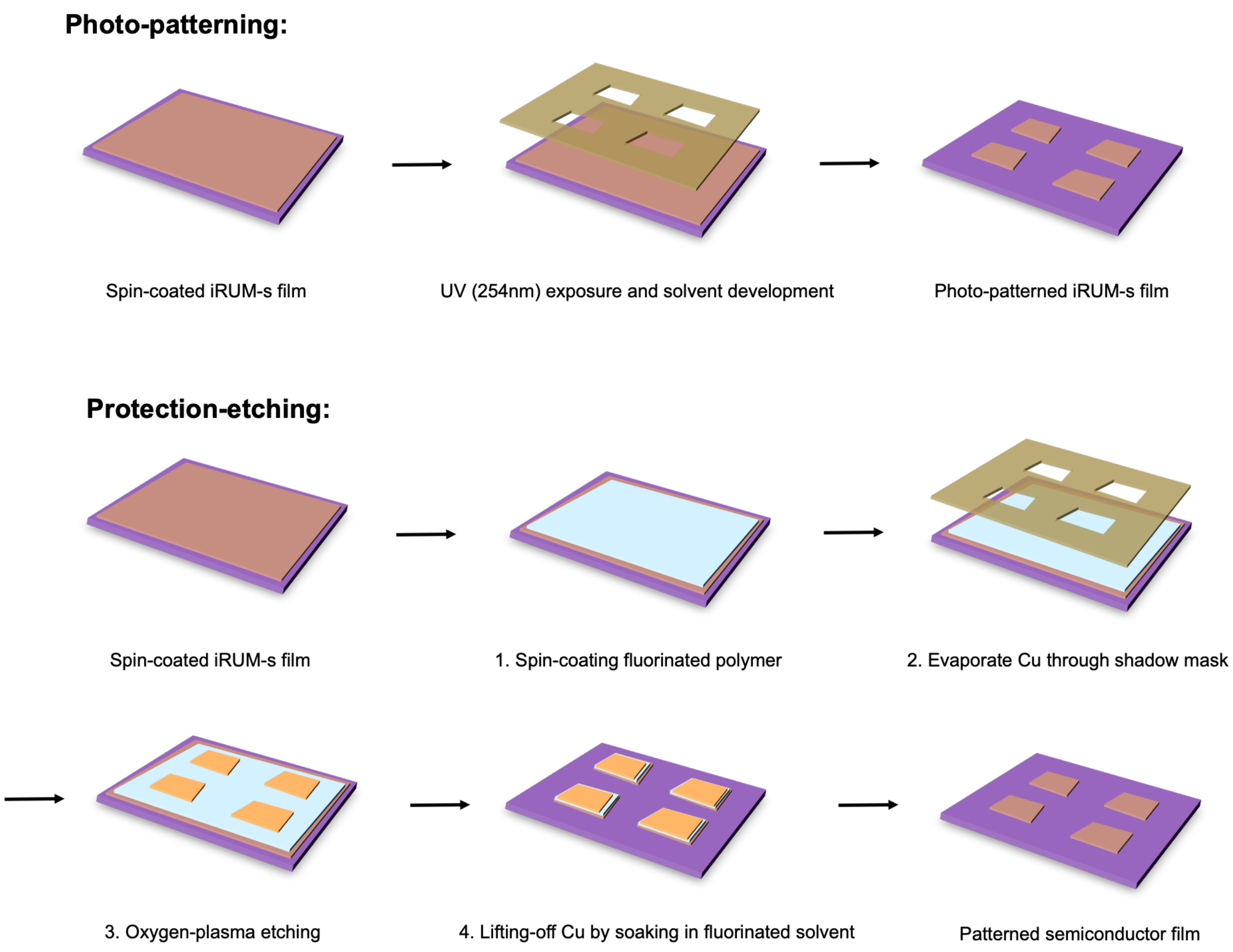

Supplementary Figure 66. Comparison of different patterning methods for stretchable semiconductor. Photo-patterning takes less steps than protection-etching patterning[12], which simplifies the fabrication process.

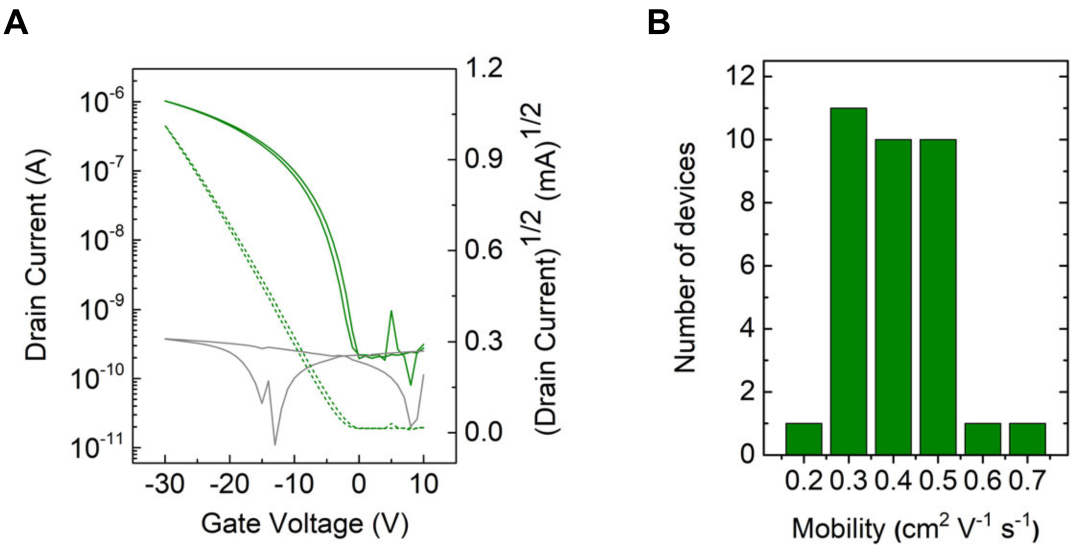

Supplementary Figure 67. (**A**) A representative transfer curve (**B**) mobility distribution from a fully-patterned elastic transistor array, with photo-patterned iRUM-s-1:1 as the semiconductor layer (40 nm thickness) and iRUM-d-4:5 as the dielectric layer (1.2 μm thickness). Carbon nanotube (CNT) serves as gate and source/drain electrodes (W= 400 μm, L= 120 μm). The transistor is in bottom-gate top-contact configuration. (Green solid lines: drain current; gray solid lines: gate current; dash lines: square root of the drain current).

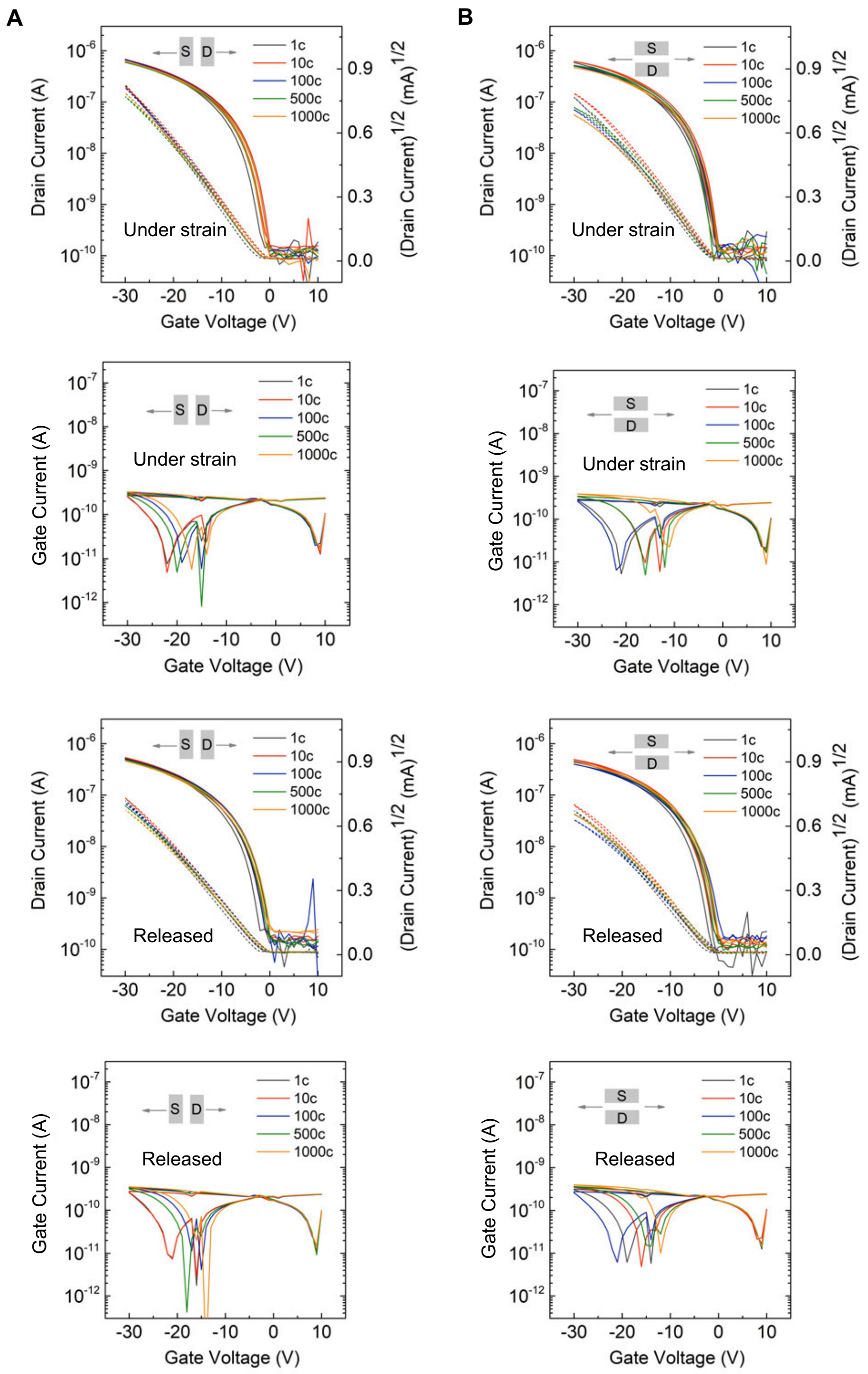

Supplementary Figure 68. Transfer curves from a fully-patterned elastic transistor array, under stretched or released state after multiple stretching-releasing cycles at 50% strain, with charge transport (**A**) parallel and (**B**) perpendicular to stretching direction (solid lines: drain/gate current; dash lines: square root of the drain current). The transistor is in bottom-gate top-contact configuration, with photo-patterned iRUM-s-1:1 as the semiconductor layer (40 nm thickness) and iRUM-d-4:5 as the dielectric layer (1.2 μm thickness). Carbon nanotube (CNT) serves as gate and source/drain electrodes (W= 400 μm, L= 120 μm).

3. Supplementary References

1. Wang, G. J. N. *et al.* Nonhalogenated Solvent Processable and Printable High-Performance Polymer Semiconductor Enabled by Isomeric Nonconjugated Flexible Linkers. *Macromolecules* **51**, 4976–4985 (2018).
2. Zhang, W. *et al.* Indacenodithiophene Semiconducting Polymers for High-Performance, Air-Stable Transistors. *J. Am. Chem. Soc.* **132**, 11437–11439 (2010).
3. Abraham, M. J. *et al.* GROMACS: High performance molecular simulations through multi-level parallelism from laptops to supercomputers. *SoftwareX* **1**–**2**, 19–25 (2015).
4. Jorgensen, W. L., Maxwell, D. S. & Tirado-Rives, J. Development and Testing of the OPLS All-Atom Force Field on Conformational Energetics and Properties of Organic Liquids. *J. Am. Chem. Soc.* **118**, 11225–11236 (1996).
5. Jiang, L., Rogers, D. M., Hirst, J. D. & Do, H. Force Fields for Macromolecular Assemblies Containing Diketopyrrolopyrrole and Thiophene. *J. Chem. Theory Comput.* **16**, 5150–5162 (2020).
6. Kim, J.-H. *et al.* Tensile testing of ultra-thin films on water surface. *Nat. Commun.* **4**, 2520 (2013).
7. Zhang, S. *et al.* Probing the Viscoelastic Property of Pseudo Free-Standing Conjugated Polymeric Thin Films. *Macromol. Rapid Commun.* **39**, 1800092 (2018).
8. Kang, J. *et al.* Tough and Water-Insensitive Self-Healing Elastomer for Robust Electronic Skin. *Adv. Mater.* **30**, 1706846 (2018).
9. Poe, R., Schnapp, K., Young, M. J. T., Grayzar, J. & Platz, M. S. Chemistry and Kinetics of Singlet (Pentafluorophenyl)nitrene. *J. Am. Chem. Soc.* **114**, 5054–5067 (1992).
10. Young, M. J. T. & Platz, M. S. Mechanistic analysis of the reactions of (pentafluorophenyl)nitrene in alkanes. *J. Org. Chem.* **56**, 6403–6406 (1991).
11. Kim, J.-S. *et al.* Tuning Mechanical and Optoelectrical Properties of Poly(3-hexylthiophene) through Systematic Regioregularity Control. *Macromolecules* **48**, 4339–4346 (2015).
12. Wang, S. *et al.* Skin electronics from scalable fabrication of an intrinsically stretchable transistor array. *Nature* **555**, 83–88 (2018).
