# Supplementary material for: A molecular design approach towards elastic and multifunctional polymer electronics
Source: Nat Commun. 2021 Sep 29;12:5701. doi: 10.1038/s41467-021-25719-9 (PMC8481247; doi:10.1038/s41467-021-25719-9)
Supplement: Supplementary file 2 — Description of Additional Supplementary Files [file 41467_2021_25719_MOESM2_ESM.pdf]

**Description of Additional Supplementary Files**

File Name: Supplementary Movie 1
Description: $180^{o}$ Peeling test movie showing the existence of interfacial crosslinking between BA rubber and PDMS
